# Gastric digestion-induced transformation of acorn phenolics from *Quercus rubra* and *Quercus robur*: antioxidant activity, multi-target enzyme inhibition, and molecular docking insights

**DOI:** 10.1039/d6ra08016b

**Published:** 2026-11-02

**Authors:** Aleksandra Popowska, Barbora Strasserova, Marcin Szustak, Gabriela Kowalska, Kamila Kulbat-Warycha, Aristeidis S. Tsagkaris, Joanna Oracz

**Affiliations:** a Institute of Food Technology and Analysis, Faculty of Biotechnology and Food Sciences, Lodz University of Technology 2/22 Stefanowskiego Street 90-537 Lodz Poland joanna.oracz@p.lodz.pl; b Department of Food Analysis and Nutrition, Faculty of Food and Biochemical Technology, University of Chemistry and Technology Technická 5 166 28 Prague Czech Republic Aristeidis.Tsagkaris@vscht.cz; c Institute of Molecular and Industrial Biotechnology, Faculty of Biotechnology and Food Sciences, Lodz University of Technology Lodz 90-537 Poland

## Abstract

Acorns, the fruits of *Quercus* spp., represent an underutilized source of bioactive phenolic compounds. In this study, phenolic-rich fractions isolated from *Quercus rubra* and *Quercus robur* acorns were evaluated to characterize their bioactive phenolic profiles, antioxidant properties, and inhibitory activity against multiple enzyme targets, and to determine how simulated gastric digestion affects these biological activities. UHPLC-DAD-ESI-HRMS/MS analysis revealed that the two *Quercus* fractions differed markedly in their individual phenolic profiles, especially in ellagitannin composition. Despite compositional changes, both native and gastric-digested fractions retained considerable antioxidant and multi-target enzyme-inhibitory activity. The *Q. rubra* fraction, rich in oenothein B and rugosin E isomers, showed stronger acetylcholinesterase (AChE) inhibition (IC_50_: 0.25 mg mL^−1^), whereas the *Q. robur* fraction, characterized by castalagin/vescalagin-type compounds and tellimagrandin II isomers, was more active against tyrosinase (TYR), α-amylase (AMY), α-glucosidase (GLU) (IC_50_: 0.080, 0.22, and 1.8, mg mL^−1^, respectively). Notably, simulated gastric digestion preserved or even enhanced the inhibitory activity of the *Q. rubra* fraction against pancreatic lipase (PNLIP), AMY, as well as α-GLU. Molecular docking analysis showed that the predominant ellagitannins identified in both oak fractions exhibited strong predicted affinities toward the multiple enzyme targets. Tellimagrandin II showed particularly high affinity for AChE and TYR (−10.6 and −8.6 kcal mol^−1^, respectively). Oenothein B showing the highest affinity for GLU (−11.3 kcal mol^−1^) and vescavaloninic acid for AMY (−10.2 kcal mol^−1^). Castalagin showed the strongest predicted interaction with PNLIP (−9.3 kcal mol^−1^). Interestingly, low-molecular-weight phenolics whose levels increased after gastric digestion, particularly ellagic acid, also exhibited strong predicted affinities for AChE (−10.0 kcal mol^−1^) and PNLIP (−9.2 kcal mol^−1^). These results suggest that *Q. rubra* and *Q. robur* acorns may represent valuable sources of bioactive compounds with antioxidant and enzyme-modulating potential, particularly toward enzymes associated with neurodegenerative processes, melanogenesis, carbohydrate digestion, and lipid metabolism.

## Abstract 2

Gastric digestion remodeled hydrolysable tannins in *Quercus* acorn fractions while preserving antioxidant and multi-enzyme inhibitory activity. Docking revealed key enzyme-interacting phenolics.

## Graphical abstract

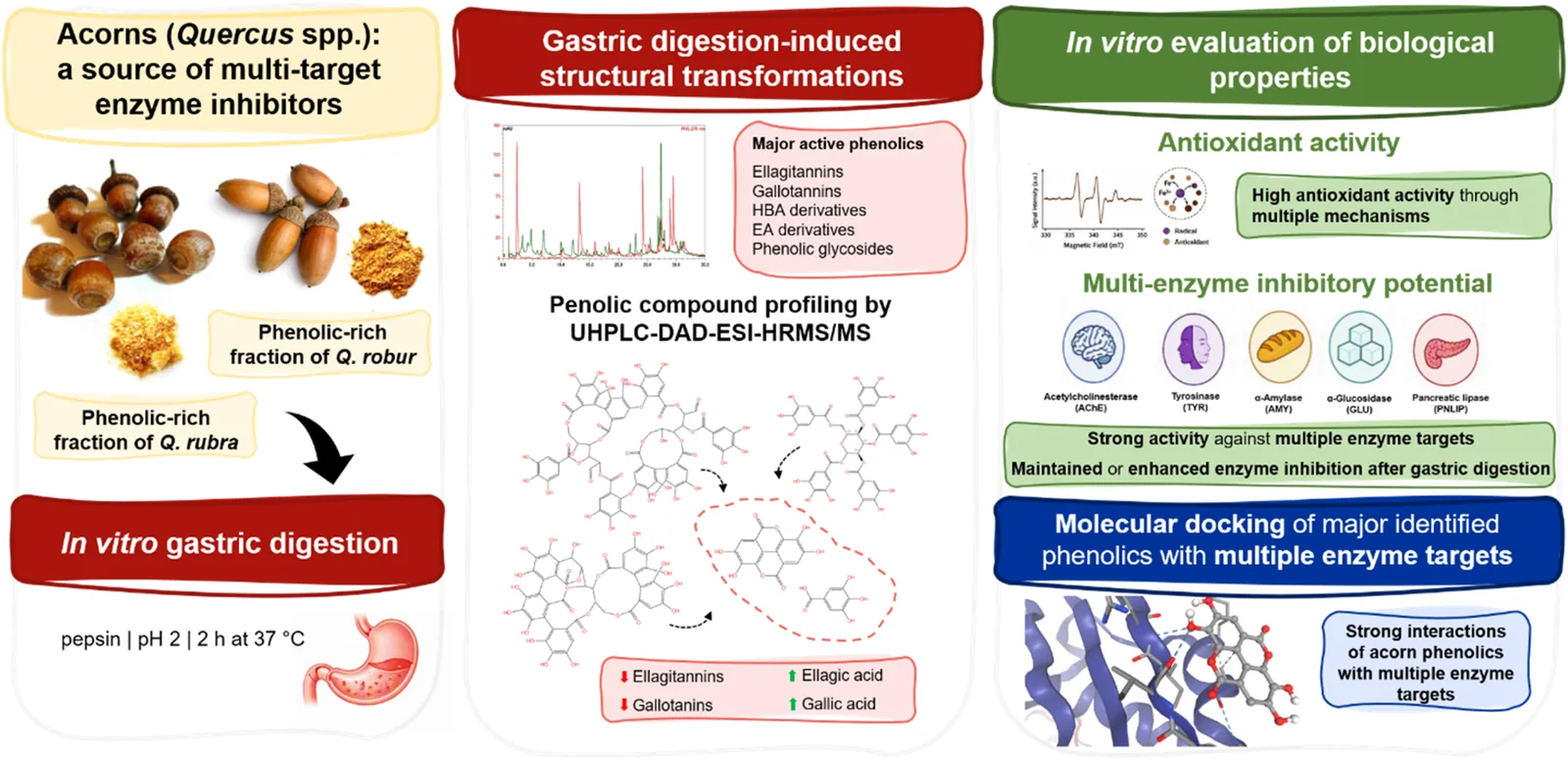

## 1 Introduction

In recent years, growing attention has been directed toward plant materials as sustainable and valuable sources of health-promoting compounds.^1^ Among them, acorns, the fruits of trees belonging to the genus *Quercus*, have attracted particular interest due to their high content of bioactive constituents, especially phenolic compounds, which exhibit diverse biological and pharmacological activities.^2–4^

Acorns contain a complex mixture of phenolic compounds, including gallic acid (GA) and ellagic acid (EA) derivatives, gallotannins (GTs), ellagitannins (ETs), flavan-3-ols, and other phenolic glycosides.^4–6^ Hydrolysable tannins are of particular interest due to their structure, which contains numerous hydroxyl groups and ester linkages. This enables them to act as radical scavengers, reducing agents, metal chelators and protein-binding compounds.^3^

In addition to their antioxidant properties, the ability to inhibit specific enzymes is an important indicator of the multifunctional biological potential of plant phenolic compounds. These compounds may interact with enzymes associated with neurodegenerative processes, melanogenesis, carbohydrate digestion, and lipid metabolism. Such activity may be relevant to the prevention or management of metabolic and neurodegenerative disorders, including type 2 diabetes, obesity, and Alzheimer's disease.^7,8^ Inhibition of α-amylase (AMY) and α-glucosidase (GLU) may be important for regulating postprandial glucose levels, while the inhibition of pancreatic lipase (PNLIP) may help to modulate lipid digestion and absorption.^7^ Tyrosinase (TYR) inhibition is associated with the control of enzymatic browning and melanogenesis, while acetylcholinesterase (AChE) inhibition is commonly investigated in relation to neuroprotective potential.^8^ Since these enzymes differ in structure and catalytic mechanism, broad enzyme-inhibitory screening may provide valuable insight into the multifunctionality of phenolic compounds.^7^

Recent studies on plants belonging to the genus *Quercus* have confirmed their significant potential as sources of enzyme inhibitors, particularly in relation to enzymes involved in carbohydrate metabolism. Extracts from several species, including *Q. suber*, *Q. coccifera*, *Q. ilex*, *Q. mongolica*, and *Q. robur*, have demonstrated strong inhibitory effects against α-amylase and α-glucosidase.^7,9,10^ Additionally, several *Quercus* extracts have been reported to exhibit AChE inhibitory activity, suggesting their potential application in the prevention and treatment of neurodegenerative disorders.^11^ Evidence regarding pancreatic lipase inhibition remains limited and inconsistent.^12^ The enzyme-inhibitory activity of *Quercus*-derived products is strongly influenced by the species, plant matrix, including acorns, bark, or leaves, and phenolic composition. In particular, tannin-rich fractions appear to play a key role in interactions with enzyme proteins, contributing to the observed inhibitory effects.^13,14^ However, under gastrointestinal conditions, these compounds may undergo hydrolysis, depolymerization, or other structural transformations, resulting in the release of smaller phenolic molecules with different solubility, stability, bioaccessibility, and biological activity than their precursors.^15,16^ Evaluating digestive stability and digestion-induced transformations is essential to understanding how acorn phenolic compounds retain their biological activity after gastric digestion.

Therefore, the aim of this study was to characterize the phenolic profiles and gastric digestion-induced structural transformations of phenolic-rich fractions obtained from *Q. rubra* and *Q. robur* acorns, and to determine the effect of these changes on their antioxidant and multi-target enzyme-inhibitory activities. The phenolic composition of the crude and gastric-digested fractions was analysed using UHPLC-DAD-ESI-HRMS/MS, and their antioxidant potential was evaluated using spectrophotometric *in vitro* assays and electron paramagnetic resonance (EPR) spectroscopy. The inhibitory activity of the phenolic acorn fractions was evaluated against AChE, TYR, PNLIP, AMY, and GLU. Special attention was given to the structural changes in phenolic compounds caused by the digestion process, as well as to how these changes affected the preservation or modulation of biological activity. Molecular docking, ADME (Absorption, Distribution, Metabolism, Elimination) prediction, and toxicity profiling were performed for the major identified phenolic compounds to provide further insight into structure-related enzyme interactions, pharmacokinetic behaviour, and safety profiles. Docking studies helped elucidate the interactions between the major phenolic constituents, including ellagitannins, gallotannins, ellagic acid, and gallic acid, and their target enzymes. These findings provide new insight into the multi-target enzyme-inhibitory potential of phenolic fractions from *Q. rubra* and *Q. robur* acorns, supporting their further investigation as bioactive plant-derived fractions with enzyme-modulating potential following simulated gastric digestion.

## 2 Results and discussion

### 2.1. Phenolic profile of *Quercus* fractions

The phenolic-rich fractions obtained from *Q. rubra* and *Q. robur* acorns were comprehensively characterized using UHPLC-DAD-ESI-HRMS/MS. Among the compounds identified in both acorn fractions, hydrolysable tannins predominated, including ellagitannins (ET) and gallotannins (GT), as well as gallic acid (GA), ellagic acid (EA), their derivatives, and phenolic glycosides. However, significant differences were observed in their individual phenolic profiles, particularly with regard to ellagitannin content. The *Q. rubra* fraction was mainly characterized by oenothein B, rugosin E isomers, and cretanin, together with galloylglucose, punicalagin, tellimagrandin, glansrin D/degalloylated rugosin F, eucalbanin, casuarictin isomers, and EA derivatives (Table 1 and Fig. S1A). In contrast, the *Q. robur* fraction showed a particularly complex profile of castalagin/vescalagin-type ETs, including vescalagin/castalagin isomers, vescavaloninic and castavescavaloninic acids, pedunculagin, galloyl-HHDP-glucose, geraniin, gemin D, casuarictin, chebulagic acid, galloylglucose, and EA derivatives (Table 2 and Fig. S1B).

**Table 1:** Phenolic compounds identified and quantified in *Q. rubra* fractions by UHPLC-DAD-ESI-HR-MS/MS^a^

No.	*R*_t_ (min)	Compound	*λ*_max_ (nm)	[M − H]^−^ observed (*m*/*z*)	Theoretical mass (*m*/*z*)	Mass error (ppm)	Fragment ions MS2 (*m*/*z*)	Concentration (mg g^−1^)	
								Crude fraction	Gastric fraction
1	2.40	Gallic acid	213.2, 270.7	169.0139	169.0142	−2.05	125.02, 97.03	15.69 ± 0.12a	24.94 ± 0.16b
2	3.37	Galloylglucose (1)	216.7, 276.1	331.0671	331.0671	0.09	271.05, 169.01, 125.02	0.01 ± 0.01a	3.33 ± 0.01b
3	4.05	Galloylglucose (2)	216.1, 275.8	331.0670	331.0671	−0.21	271.05, 169.01, 125.02	0.50 ± 0.05b	0.60 ± 0.08a
4	6.65	(−)-Gallocatechin	202.1, 269.9	305.0656	305.0667	−3.53	287.06, 179.03, 125.02	5.27 ± 0.17b	2.32 ± 0.03a
5	8.47	Gallic acid O-glucoside	204.3, 272.3	331.0665	331.0671	−1.73	169.01, 125.02	0.36 ± 0.06b	0.17 ± 0.01a
6	9.30	Punicalagin isomer (1)	258.5378.0	1083.0624	1083.0593	2.90	1065.02, 631.04, 450.98, 300.99	0.13 ± 0.02a	0.83 ± 0.06b
7	9.65	Digalloylglucose (1)	212.3, 275.7	483.0769	483.0780	−2.34	331.07, 313.06, 169.01	0.17 ± 0.03b	0.14 ± 0.01a
8	10.04	Methyl gallate	213.6, 270.9	183.0298	183.0299	−0.53	168.01, 141.00	3.06 ± 0.11a	3.64 ± 0.05b
9	12.68	Digalloylglucose (2)	212.4, 272.8	483.0772	483.0780	−1.72	331.07, 313.06, 169.01	0.30 ± 0.04b	0.17 ± 0.02a
10	13.21	Oenothein B (1)	214.1, 264.0	783.0673^b^	1567.1446	−1.72	935.08, 785.08, 765.07, 633.07, 615.07, 483.08, 301.00, 169.01	47.68 ± 0.28b	42.21 ± 0.23a
11	13.49	Digalloylglucose (3)	213.7, 275.7	483.0767	483.0780	−2.75	331.07, 313.06, 169.01	0.02 ± 0.01a	0.02 ± 0.01a
12	15.00	Digalloylglucose (4)	213.5, 276.3	483.0766	483.0780	−2.96	331.07, 313.06, 169.01	0.91 ± 0.05a	0.96 ± 0.10b
13	15.91	Oenothein B (2)	214.2, 264.7	783.0671^b^	1567.1446	−1.97	935.08, 785.08, 765.07, 633.07, 615.07, 483.08, 301.00, 169.01	8.55 ± 0.13b	1.36 ± 0.03a
14	18.36	Tellimagrandin I (1)	214.2, 273.0	785.0839	785.0843	−0.50	483.06, 300.99	6.55 ± 0.14b	3.92 ± 0.09a
15	18.88	Glansrin D/degalloyl rugosin F (1)	212.3, 266.9	859.0723^b^	1719.1555	−2.12	1087.09, 935.08, 917.07, 785.08, 783.07, 765.06, 749.06, 633.07, 615.06, 465.07, 450.99, 301.00, 275.02, 169.01	3.08 ± 0.08b	0.66 ± 0.03a
16	19.64	Trigalloylglucose (1)	215.3, 276.9	635.0891	635.0890	0.18	465.07, 313.06, 169.01	0.95 ± 0.05b	0.79 ± 0.08a
17	20.04	Glansrin D/degalloyl rugosin F (2)	213.4, 265.3	859.0725^b^	1719.1555	−1.89	1087.09, 935.08, 917.07, 785.08, 783.07, 765.06, 749.06, 633.07, 615.06, 465.07, 450.99, 301.00, 275.02, 169.01	6.13 ± 0.13b	3.45 ± 0.13a
18	20.50	Punicalagin isomer (2)	258.5, 378.0	1083.0622	1083.0593	2.72	1065.02, 631.04, 450.98, 300.99	1.23 ± 0.07b	0.47 ± 0.11a
19	21.42	Trigalloylglucose (2)	215.3, 276.9	635.0892	635.0890	0.34	465.07, 313.06, 169.01	0.24 ± 0.05a	0.61 ± 0.05b
20	22.00	Valoneic acid dilactone	254.9, 365.4	469.0037	469.0049	−2.48	425.02, 301.00, 299.99	0.34 ± 0.03b	0.01 ± 0.01a
21	22.52	Tellimagrandin I (2)	216.0, 270.0	785.0836	785.0843	−0.89	483.06, 300.99	1.55 ± 0.08b	0.86 ± 0.07a
22	22.95	Ellagic acid glucoside	252.8, 363.4	463.0509	463.0518	−1.95	300.99	2.07 ± 0.09b	0.01 ± 0.01a
23	23.22	Eucalbanin B/eucalbanin C (1)	214.2, 262.1	784.0750^b^	1569.1602	−1.88	785.08, 765.06, 633.07, 483.08, 450.99, 331.07, 301.00, 169.01	0.17 ± 0.04a	0.37 ± 0.17b
24	24.20	Cretanin	202.5, 274.4	469.0986	469.0987	−0.26	169.01	45.15 ± 0.19b	41.91 ± 0.15a
25	25.19	Eucalbanin B/eucalbanin C (2)	215.2, 261.4	784.0752^b^	1569.1602	−1.62	785.08, 765.06, 633.07, 483.08, 450.99, 331.07, 301.00, 169.01	6.60 ± 0.11s	7.82 ± 0.09b
26	25.66	Casuarictin (1)	219.0, 280.0	935.0787	935.0796	−1.50	917.04, 873.05, 783.05	2.08 ± 0.05b	0.58 ± 0.03a
27	26.09	Tetragalloylglucose (1)	213.9, 277.6	787.1002	787.0999	0.32	617.01, 465.07, 313.05169.01	0.61 ± 0.03b	0.28 ± 0.05a
28	26.26	Punicalagin isomer (3)	259.6, 369.5	1083.0620	1083.0593	2.54	1065.02, 631.04, 450.98, 300.99	0.41 ± 0.02b	0.34 ± 0.08a
29	26.46	Tetragalloylglucose (2)	213.9, 277.6	787.1006	787.0999	0.83	617.01, 465.07, 313.05, 169.01	0.40 ± 0.01b	0.33 ± 0.03a
30	26.83	Tellimagrandin II (1)	217.9, 277.9	937.0965	937.0953	1.33	785.07, 767.07, 465.06, 301.00	2.91 ± 0.11b	2.14 ± 0.12a
31	27.11	Punicalagin isomer (4)	259.6, 360.5	1083.0618	1083.0593	2.35	1065.02, 631.04, 450.98, 300.99	0.08 ± 0.01b	0.06 ± 0.01a
32	27.30	Ellagic acid	252.8, 356.4	300.9983	300.9990	−2.30	257.01	4.01 ± 0.09a	8.09 ± 0.09b
33	27.75	Punicalagin isomer (4)	258.6, 375.5	1083.0621	1083.0593	2.63	1065.02, 631.04, 450.98, 300.99	1.52 ± 0.07b	0.07 ± 0.02a
34	27.94	Tetragalloylglucose (3)	213.9, 277.6	787.1006	787.0999	0.83	617.01, 465.07, 313.05, 169.01	2.12 ± 0.05b	1.44 ± 0.02a
35	28.95	Rugosin E (1)	215.0, 274.9	860.0810^b^	1721.1712	−1.10	937.09, 917.07, 785.08, 765.06, 749.06, 633.07, 483.08, 465.07, 450.99, 301.00, 275.02, 169.01	20.12 ± 0.19b	15.91 ± 0.14a
36	29.51	Rugosin E (2)	214.7, 275.4	860.0809^b^	1721.1712	−1.22	1087.09, 937.09, 917.07, 785.08, 765.06, 749.06, 633.07, 483.08, 465.07, 450.99, 301.00, 275.02, 169.01	35.72 ± 0.21a	40.03 ± 0.28b
37	30.01	Rugosin D (1)	208.2, 274.1	936.0876^b^	1873.1821	0.18	1087.09, 937.09, 917.07, 785.08, 767.06, 749.06, 615.06, 465.07, 450.99, 301.00, 275.02, 169.01	2.73 ± 0.03b	1.08 ± 0.07a
38	30.72	Pentagalloylglucose (1)	215.5, 279.0	939.1108	939.1109	−0.11	787.08, 769.07, 617.06	0.85 ± 0.05b	0.18 ± 0.05a
39	31.11	Pentagalloylglucose (2)	214.3, 279.6	939.1102	939.1109	−0.75	787.08, 769.07, 617.06	2.36 ± 0.09b	0.90 ± 0.08a
40	31.40	Rugosin D (2)	205.0, 271.8	936.0851^b^	1873.1821	−2.49	1087.09, 937.09, 917.07, 785.08, 767.06, 749.06, 615.06, 465.07, 450.99, 301.00, 275.02, 169.01	4.03 ± 0.05b	0.51 ± 0.07a

a *R*_t_ – retention time; *λ*_max_ – maximum absorbance.

b Doubly charged ion [M − 2H]^2−^; the values are expressed as the mean ± SD (*n* = 3); different letters within the same row indicate statistically significant differences between the crude extract and the gastric fraction according to Tukey's test at *p* < 0.05.

**Table 2:** Phenolic compounds identified and quantified in *Q. robur* fractions by UHPLC-DAD-ESI-HR-MS/MS^a^

No.	*R*_t_ (min)	Compound	*λ*_max_ (nm)	[M − H]^−^ observed (*m*/*z*)	Theoretical mass (*m*/*z*)	Mass error (ppm)	Fragment ions MS2 (*m*/*z*)	Concentration (mg g^−1^)	
								Crude fraction	Gastric fraction
1	2.40	Gallic acid	213.0, 270.2	169.0138	169.0142	−2.64	125.02, 97.03	0.59 ± 0.02a	1.64 ± 0.07b
2	3.34	Vescavaloninic acid	223.8	1101.0674	1101.0698	−2.18	1083.03, 1039.04, 995.05, 915.03, 569.04, 425.00, 300.99	7.63 ± 0.15b	5.28 ± 0.02a
3	4.27	Vescalagin (1)	221.8	933.0651	933.0640	1.23	633.05, 613.03, 425.00, 300.99	4.58 ± 0.08b	4.20 ± 0.02a
4	4.82	Castavaloninic acid	223.8	1101.0665	1101.0698	−3.00	1083.03, 1039.04, 995.05, 915.03, 569.04, 425.00, 300.99	12.52 ± 0.16a	14.95 ± 0.10b
5	5.94	Vescalagin (2)	221.8	933.0652	933.0640	1.33	633.05, 613.03, 425.00, 300.99	1.30 ± 0.05a	1.80 ± 0.05b
6	7.02	Castalagin (1)	221.8	933.0653	933.0640	1.44	633.05, 613.03, 425.00, 300.99	14.09 ± 0.15b	12.56 ± 0.12a
7	8.17	Digalloylglucose (1)	213.7, 275.7	483.0783	483.0780	0.56	331.07, 313.06, 169.01	0.69 ± 0.05a	0.70 ± 0.07a
8	10.05	Methyl galate	214.0, 270.9	183.0296	183.0299	−1.62	168.01, 141.00	9.42 ± 0.09b	2.56 ± 0.04a
9	10.44	Di-HHDP-glucose (1)	228.7	783.0682	783.0686	−0.54	481.04, 300.99	3.05 ± 0.05a	5.10 ± 0.13b
10	12.10	Vescalagin/castalagin isomer (1)	197.0, 222.7	933.0655	933.0640	1.66	633.05, 613.03, 425.00, 300.99	7.56 ± 0.14b	3.42 ± 0.15a
11	13.54	Digalloylglucose (2)	213.9, 272.9	483.0785	483.0780	0.97	331.07, 313.06, 169.01	1.74 ± 0.05a	1.92 ± 0.09b
12	14.54	Vescalagin/castalagin isomer (2)	197.0, 222.7	933.0650	933.0640	1.12	633.05, 613.03, 425.00, 300.99	1.68 ± 0.09b	0.81 ± 0.04a
13	14.99	Digalloylglucose (3)	213.7, 275.2	483.0784	483.0780	0.77	331.07, 313.06, 169.01	0.52 ± 0.05a	0.81 ± 0.02b
14	15.78	Trigalloylglucose (1)	215.3, 276.9	635.0896	635.0890	0.97	465.07, 313.06, 169.01	2.50 ± 0.10a	2.76 ± 0.08b
15	16.25	Galloyl-HHDP-glucose (1)	215.8, 280.0	633.0739	633.0733	0.91	481.05, 463.04, 300.99	1.67 ± 0.04b	1.36 ± 0.05a
16	17.03	Galloyl-HHDP-glucose (2)	215.8, 279.7	633.0738	633.0733	0.75	481.05, 463.04, 300.99	1.49 ± 0.02a	1.92 ± 0.04b
17	17.76	Galloyl-HHDP-glucose (3)	215.8, 278.9	633.0736	633.0733	0.44	481.05, 463.04, 300.99	4.36 ± 0.09b	1.28 ± 0.03a
18	18.38	Tellimagrandin I (1)	214.2, 273.0	785.0852	785.0843	1.15	483.06, 300.99	3.71 ± 0.08b	3.21 ± 0.07a
19	18.62	Geraniin	216.4, 276.8	951.0734	951.0745	−1.18	907.06, 783.05, 605.06, 481.05, 300.99	2.93 ± 0.11a	4.17 ± 0.09b
20	19.42	Digalloylglucose (4)	214.1, 278.7	483.0786	483.0780	1.18	331.07, 313.06, 169.01	0.26 ± 0.01a	0.34 ± 0.06b
21	20.15	Punicalagin isomer (1)	254.8, 368.9	1083.0620	1083.0593	2.54	1065.02, 631.04, 450.98, 300.99	1.27 ± 0.02a	1.55 ± 0.05b
22	21.42	Trigalloylglucose (2)	215.3, 276.9	635.0894	635.0890	0.66	465.07, 313.06, 169.01	0.73 ± 0.03a	1.12 ± 0.04b
23	22.01	Valoneic acid dilactone	254.9, 365.4	469.0035	469.0049	−2.91	425.02, 301.00, 299.99	4.06 ± 0.12b	0.50 ± 0.05a
24	22.28	Gemin D	214.0, 267.1	633.0745	633.0733	1.86	481.05, 463.04, 300.99	0.45 ± 0.02a	1.75 ± 0.04b
25	22.93	Ellagic acid glucoside	255.8, 363.4	463.0507	463.0518	−2.38	300.99	3.85 ± 0.12b	0.17 ± 0.01a
26	23.91	Tellimagrandin I (2)	216.3, 275.8	785.0850	785.0843	0.90	483.06,300.99	1.77 ± 0.04b	1.62 ± 0.03a
27	24.77	Trigalloylglucose (3)	215.3, 276.9	635.0898	635.0890	1.28	465.07, 313.06, 169.01	1.05 ± 0.02a	1.15 ± 0.05
28	25.27	Tetragalloylglucose (1)	213.9, 277.6	787.1008	787.0999	1.08	617.01, 465.07, 313.05, 169.01	0.68 ± 0.03a	1.97 ± 0.02b
29	25.490	Casuarictin (1)	216.3, 276.9	935.0781	935.0796	−1.61	917.04, 873.05, 783.05	6.47 ± 0.12b	0.83 ± 0.03a
30	25.897	Casuarictin (2)	216.3, 277.6	935.0782	935.0796	−1.50	917.04, 873.05, 783.05	0.72 ± 0.05a	1.53 ± 0.05b
31	26.96	Tellimagrandin II	215.0, 277.9	937.0964	937.0953	1.22	785.07, 767.07, 465.06, 301.00	18.05 ± 0.19a	10.78 ± 0.14b
32	27.11	Punicalagin isomer (2)	254.8, 368.9	1083.0616	1083.0593	2.17	1065.02, 631.04, 450.98, 300.99	6.30 ± 0.07b	0.56 ± 0.05a
33	27.30	Ellagic acid	252.8, 356.4	300.9986	300.9990	−1.30	257.01	22.34 ± 0.25a	38.70 ± 0.28b
34	27.76	Tetragalloylglucose (2)	213.9, 277.6	787.1003	787.0999	0.45	617.01, 465.07, 313.05, 169.01	0.45 ± 0.05a	0.71 ± 0.05b
35	27.95	Tetragalloylglucose (3)	213.9, 277.6	787.1005	787.0999	0.70	617.01, 465.07, 313.05, 169.01	6.01 ± 0.04b	0.81 ± 0.02a
36	28.94	Chebulagic acid	214.0, 272.0	953.0912	953.0902	1.08	935.05, 783.05, 794.04	2.85 ± 0.07b	0.53 ± 0.04a
37	30.29	Casuarictin (3)	219.0, 285.0	935.0784	935.0796	−1.29	917.04, 765.04, 749.04, 300.99	2.01 ± 0.03a	2.16 ± 0.02b
38	30.59	Pentagalloylglucose (1)	215.5, 279.0	939.1108	939.1109	−0.11	787.08, 769.07, 617.06	3.73 ± 0.04a	7.01 ± 0.09b
39	31.01	Pentagalloylglucose (2)	214.2, 279.1	939.1102	939.1109	−0.75	787.08, 769.07, 617.06	5.20 ± 0.05b	0.21 ± 0.05a

a *R*_t_ – retention time; *λ*_max_ – maximum absorbance; The values are expressed as the mean ± SD (*n* = 3); different letters within the same row indicate statistically significant differences between the crude extract and the gastric fraction according to Tukey's test at *p* < 0.05.

Although several of these compounds have previously been reported in acorns and other *Quercus* tissue^5,6,17–19^ this study provides a comprehensive comparative characterization of phenolic-rich acorn fractions from *Q. rubra* and *Q. robur*, including detailed identification and quantification of hydrolysable tannins, phenolic acids, and their derivatives. The results confirm that both oak species are valuable sources of bioactive phenolics. These compounds may contribute to the biological activity of oak-derived products due to their numerous hydroxyl groups, redox properties, and ability to interact with proteins, including digestive and metabolic enzymes.^18,20,21^

The extraction yields of the freeze-dried phenolic-rich fractions were 34.68% and 36.54% for *Q. rubra* and *Q. robur* acorns, respectively. Based on the sum of individual phenolic compounds identified by UHPLC-DAD-ESI-HRMS/MS, the initial acorn powders contained 107.78 and 69.18 mg g^−1^ of identified phenolics for *Q. rubra* and *Q. robur*, respectively. The corresponding freeze-dried crude fractions contained 235.67 and 170.26 mg g^−1^ of identified phenolics, respectively. These values corresponded to enrichment factors of 2.19 for *Q. rubra* and 2.46 for *Q. robur*. The recoveries of identified phenolic compounds were 75.83% and 89.93%, respectively. These results confirm that the applied extraction and freeze-drying procedure effectively concentrated phenolic compounds from both acorn materials and support the practical feasibility of obtaining acorn-derived phenolic-rich fractions.^18^

Simulated gastric digestion substantially modified the qualitative and quantitative phenolic profiles of both acorn fractions (Fig. S1, Tables 1 and 2).

The total content of identified phenolics decreased, indicating partial degradation or transformation of hydrolysable tannins, especially high-molecular-weight and glycosylated forms, under gastric conditions. In the *Q. rubra* acorn phenolic fraction, simulated gastric digestion caused a marked qualitative rearrangement, accompanied by a moderate decrease in total identified phenolics from 235.67 to 213.49 mg g^−1^. This indicates that, despite partial degradation or transformation of selected compounds, a substantial part of the *Q. rubra* phenolic fraction remained stable under gastric conditions (Fig. S1A). Before digestion, the *Q. rubra* profile was dominated by ETs, mainly oenothein B and rugosin E isomers. After digestion, ETs remained the predominant group, but their composition was clearly modified, suggesting partial hydrolysis, isomerization, transformation of structurally related compounds, and release of EA and intermediate ET derivatives under acidic conditions.^22^

This is consistent with the structure of ETs (Fig. S2), which contain HHDP and galloyl units linked by ester bonds that may undergo cleavage, followed by spontaneous lactonization of HHDP units to EA.^22^ Among the major ETs, oenothein B isomers decreased from 56.23 to 43.57 mg g^−1^, whereas rugosin E isomers were largely preserved overall, although individual isomers showed opposite trends, suggesting possible interconversion or release from more complex precursors. Tellimagrandin I, glansrin D/degalloyl rugosin F, casuarictin, and rugosin D decreased markedly, indicating greater susceptibility to gastric degradation. In contrast, tellimagrandin II showed only a minor decrease, while eucalbanin B/C isomers slightly increased, possibly due to secondary formation or release under acidic conditions.

Considerable transformations were also observed among GTs. The increase in galloylglucose, together with the rise in free GA, indicates degalloylation of more complex tannins. Digalloylglucose remained relatively stable, whereas trigalloylglucose slightly increased, suggesting that these compounds may represent intermediate products of tannin hydrolysis. Tetragalloylglucose and especially pentagalloylglucose decreased, confirming the susceptibility of highly galloylated structures to acidic hydrolysis.

Phenolic glycosides showed compound-dependent stability. Cretanin decreased only slightly, from 45.15 to 41.91 mg g^−1^, whereas EA glucoside was almost completely degraded. This was accompanied by an increase in free EA, suggesting hydrolysis of glycosidic or ester-linked precursors. The increase in GA, galloylglucose, and methyl gallate further supports the acid-catalyzed cleavage of GTs and related derivatives.

A similar, although not identical, pattern was observed for the *Q. robur* phenolic fraction (Table 2 and Fig. S1B). The total content of identified phenolics decreased by around 15%, indicating partial sensitivity to gastric conditions. As in *Q. rubra*, digestion caused both degradation and qualitative restructuring of the phenolic profile. Initially, the *Q. robur* fraction was dominated by ETs and EA derivatives, including vescalagin and castalagin-type compounds, tellimagrandin, casuarictin, and punicalagin (Fig. S2). After digestion, the most pronounced change was the increase in free EA, accompanied by a sharp decrease in EA glucoside.

Changes in GA derivatives were less pronounced in *Q. robur* than in *Q. rubra*. Although GA increased slightly, the marked decrease in methyl gallate resulted in an overall reduction in this fraction, suggesting less efficient accumulation of simple hydroxybenzoic acids. GT transformations followed trends similar to those observed in *Q. rubra*. Highly galloylated structures, particularly tetra- and pentagalloylglucose, decreased, whereas compounds with lower or intermediate degrees of galloylation remained relatively stable or increased slightly.

For ETs present in both fractions, such as punicalagin, tellimagrandin, and casuarictin, the direction of changes was generally similar and involved partial degradation, contributing to the increased pool of free EA. However, vescalagin, castalagin, and their derivatives were more characteristic of the *Q. robur* profile and showed compound-specific behaviour. Vescavaloninic acid decreased from 7.63 to 5.28 mg g^−1^, whereas castavaloneic acid increased from 12.52 to 14.95 mg g^−1^. Vescalagin, calculated as the sum of two isomers, remained relatively stable, at approximately 5.88 mg g^−1^ before gastric digestion and 6.00 mg g^−1^ after digestion, while castalagin decreased slightly from 14.09 to 12.56 mg g^−1^. By contrast, vescalagin/castalagin isomers were less stable, with their total content decreasing from approximately 9.24 to 4.23 mg g^−1^.

These results indicate that *C*-glucoside-type ETs do not behave uniformly under gastric conditions and some remain relatively stable, whereas others undergo hydrolysis, isomerization, or conversion to simpler derivatives.^22,23^ The increase in geraniin and gemin D after gastric digestion may indicate their secondary formation during the conversion of more complex ETs or their enhanced release from the matrix under acidic conditions.^22,23^ Conversely, chebulagic acid was markedly less stable, decreasing from 2.85 to 0.53 mg g^−1^. This confirms the selective nature of ET transformations, which depends on the structural features of individual compounds.^24^

The different behavior of individual compounds likely reflects their structural diversity, including degree of galloylation, oligomerization, and the presence of more stable linkages.^15,16,24,25^ The persistence of certain ETs after gastric digestion may indicate partial resistance to hydrolysis and suggests that these compounds remain present after gastric digestion, potentially contributing to biological activity during subsequent stages of digestion, whereas smaller phenolic products may be more relevant for potential systemic effects.^23^

### 2.2. *In vitro* antioxidant activity

The observed changes in phenolic composition were reflected in the antioxidant properties of the *Q. rubra* and *Q. robur* phenolic fractions. In both oak species, simulated gastric digestion slightly reduced total phenolic content (TPC), determined by the Folin–Ciocalteu method, and decreased antioxidant activity, as reflected by lower DPPH and ABTS radical scavenging capacity and ferric reducing antioxidant power (FRAP) (Table 3). In *Q. rubra* phenolic fraction, TPC decreased from 283.52 to 242.70 mg GAE g^−1^, while in *Q. robur* it decreased from 327.52 to 276.81 mg GAE g^−1^. The strongest decrease was observed in the DPPH assay, where antioxidant activity declined by approximately 23–24% in both species. The reduction was less pronounced in the ABTS assay, whereas FRAP values were relatively well preserved, especially in *Q. rubra* phenolic fraction.

**Table 3:** Antioxidant properties of *Q. rubra* and *Q. robur* acorn fractions and their changes during simulated gastric digestion^a^

Activity	*Q. rubra* crude fraction	*Q. rubra* gastric fraction	*Q. robur* crude fraction	*Q. robur* gastric fraction
TPC (mg GAE g^−1^)	283.52 ± 2.76b	242.70 ± 3.09a	327.52 ± 1.08b	276.81 ± 2.23a
TEAC_DPPH_ (µmol TE g^−1^)	2878.61 ± 11.45b	2201.58 ± 9.05a	2886.63 ± 13.87b	2208.18 ± 11.67a
TEAC_ABTS_ (µmol TE g^−1^)	3179.02 ± 12.03b	2890.02 ± 12.08a	3184.71 ± 16.56b	2898.88 ± 12.39a
TEAC_FRAP_ (µmol TE g^−1^)	2190.77 ± 10.87b	2116.69 ± 11.06a	2272.05 ± 10.51b	2068.13 ± 11.57a
AA_ox_ DPPH (µmol TE g^−1^)	1501.23 ± 0.05b	917.83 ± 0.03a	1229.49 ± 0.05b	926.53 ± 0.03a
AA_ox_ TEMPO (µmol TE g^−1^)	1213.36 ± 0.04b	742.45 ± 0.03a	1196.43 ± 0.04b	750.81 ± 0.03a

a Values are expressed as mean ± SD (*n* = 3). Different lowercase letters within the same row indicate statistically significant differences according to Tukey's HSD test (*p* < 0.05). AA_ox_ – antioxidant activity expressed as Trolox equivalents. Antioxidant activity was determined by electron paramagnetic resonance (EPR) spectroscopy using DPPH˙ and TEMPO˙ radicals.

The results of this study suggest that simulated gastric digestion only partially reduced the antioxidant potential of acorn phenolic fractions. The decrease in antioxidant activity may be associated with the partial degradation of HMW hydrolysable tannins, which have been shown to strongly contribute to radical scavenging capacity due to their high number of hydroxyl groups and ability to donate electrons or hydrogen atoms.^26^

The decrease in antioxidant activity after simulated gastric digestion may also be partially explained by interactions between HMW hydrolysable tannins and pepsin present in the gastric fluid.^27^

Phenolic compounds, including tannins, can bind to and inhibit pepsin through hydrogen bonding and hydrophobic interactions. Hydrolysable tannins, which possess many hydroxyl groups, readily form stable tannin–protein complexes, reducing the pool of free phenolics available for radical scavenging. At the same time, antioxidant activity was retained, which can be attributed to an increased concentration of smaller phenolic compounds, particularly GA and EA.

The observed changes are generally consistent with previous studies on acorn flour and acorn-enriched products subjected to *in vitro* digestion, where digestion modified both phenolic composition and antiradical properties, and where phenolic acids increased during digestion.^28^

However, available literature mainly concerns acorn flour or food matrices enriched with acorn flour rather than purified acorn fractions. Therefore, the present results provide new insight into the gastric stability of antioxidant activity in *Q. rubra* and *Q. robur* acorn phenolic fractions and suggest that gastric digestion-induced transformation of tannins changes the compounds responsible for antioxidant activity rather than eliminating this activity.

The antioxidant activity of *Q. rubra* and *Q. robur* acorn extracts was additionally evaluated by EPR spectroscopy using DPPH˙ and TEMPO˙ radicals (Table 3). The antioxidant activity measured by EPR spectroscopy differed between the two oak species and was significantly affected by simulated gastric digestion. Before digestion, *Q. rubra* phenolic fraction exhibited higher DPPH radical scavenging activity than *Q. robur* fraction, whereas TEMPO scavenging activity was comparable in both samples. Following gastric digestion, the antioxidant activity of fractions of both species decreased and reached similar values, ranging from 917.83 to 926.53 µmol TE per g for DPPH and from 742.45 to 750.81 µmol TE per g for TEMPO, indicating that digestion largely eliminated the initial differences between the fractions. The reduced EPR-derived antioxidant activity after digestion may be attributed to the transformation of hydrolysable tannins demonstrated by UHPLC-DAD-ESI-HRMS/MS analysis. In *Q. rubra*, gastric digestion decreased the concentration of oenothein B, tellimagrandin I, glansrin D/degalloyl rugosin F, casuarictin and rugosin D by 22–72%, whereas in *Q. robur* substantial reductions were observed for vescalagin/castalagin isomers, casuarictin, tellimagrandin II, punicalagins and chebulagic acid, ranging from 40% to 87%.

These compounds represent highly galloylated and HHDP-containing ETs, which are recognized as particularly efficient radical scavengers due to their high density of phenolic hydroxyl groups and strong hydrogen- and electron-donating capabilities.^29^ Importantly, the EPR results followed the same trend as the spectrophotometric assays and TPC determination. In both oak species, gastric digestion reduced total phenolic content and antioxidant activity regardless of the analytical method applied. However, the magnitude of the decrease differed among assays. Total identified phenolics determined by UHPLC decreased by 9.4% in *Q. rubra* and 15.2% in *Q. robur*, while TPC decreased by 14.4% and 15.5%, respectively. Similarly, TEAC-DPPH decreased by approximately 23.5% in both species, TEAC-ABTS by 9.1–9.4%, and TEAC-FRAP by 3.4–9.0%. In contrast, EPR-measured antioxidant activity decreased by approximately 29–39%, indicating a greater sensitivity of this technique to gastric digestion-induced changes within the phenolic fraction.

A particularly interesting observation is that the decrease in EPR activity was greater than the reduction in the overall ET fraction. In *Q. rubra*, the total ET content decreased from 150.3 to 122.7 mg g^−1^ (−18.4%), whereas DPPH-EPR activity decreased by 39.3%. Similarly, in *Q. robur* fraction, ETs decreased from 106.5 to 81.4 mg g^−1^ (−23.6%), while DPPH-EPR and TEMPO-EPR activities decreased by 29.2% and 37.5%, respectively. This indicates that antioxidant activity depends not only on the abundance of ETs but also on their structural integrity. HMW ETs appear to contribute disproportionately to radical quenching because a single molecule contains multiple galloyl and HHDP groups capable of efficient hydrogen atom donation and stabilization of radical intermediates through resonance and electron delocalization mechanisms.^30^

Despite the decrease in EPR activity, the gastric fractions retained more than 60% of the initial radical-scavenging capacity. This remaining activity may be associated with the increased abundance of LMW hydrolysis products observed in both chromatographic profiles. According to Tables 1 and 2, gastric digestion increased the concentration of GA from 15.69 to 24.94 mg g^−1^ and EA from 4.01 to 8.09 mg g^−1^ in *Q. rubra*, while in *Q. robur* GA increased from 0.59 to 1.64 mg g^−1^ and EA from 22.34 to 38.70 mg g^−1^. Nevertheless, the accumulation of these low-molecular-weight antioxidants was insufficient to compensate fully for the loss of complex ETs, highlighting the dominant contribution of hydrolysable tannins to the radical-scavenging properties of acorn phenolic fractions.

The observed relationship between phenolic composition and EPR-derived antioxidant activity is consistent with previous studies on acorn extracts, which demonstrated strong associations between tannin-rich phenolic fractions and antioxidant performance measured by DPPH, ABTS and FRAP assays.^31–33^ Similarly, transformations of hydrolysable tannins have previously been linked to significant changes in antioxidant activity, emphasizing the importance of phenolic composition and molecular structure rather than total phenolic content alone.^32^ Although direct comparison with previous EPR studies is not possible due to methodological differences, the only available EPR investigation on *Q. robur* demonstrated that changes in the acorn redox system were closely associated with free-radical processes and oxidative transformations, further highlighting the value of EPR spectroscopy for monitoring alterations in oak-derived antioxidant systems.^34^

### 2.3. Multi-target enzyme inhibitory activities

The enzyme inhibition results further indicated the high biological potential of the acorn phenolic-rich fractions. Before gastric digestion, both *Q. rubra* and *Q. robur* fractions inhibited AChE, TYR, AMY, and GLU (Table 4 and Fig. S3).

**Table 4:** IC_50_ values of acorn phenolic fractions against acetylcholinesterase, pancreatic lipase, α-amylase, tyrosinase, and α-glucosidase^a^

Enzyme assay	*Q. rubra*		*Q. robur*	
	IC_50_ (mg mL^−1^)	95% interval	IC_50_ (mg mL^−1^)	95% interval
AChE	0.25	0.040–1.5	0.41	0.15–1.1
PNLIP	No dose–response noticed			
AMY	0.61	0.43–0.86	0.22	0.15–0.33
GLU	2.2	1.1–4.5	1.8	1.2–2.7
TYR	0.14	0.086–0.25	0.080	0.053–0.12

a AChE – acetylcholinesterase, PNLIP – pancreatic lipase, AMY – α-amylase, TYR – tyrosinase, GLU – α-glucosidase. Each concentration point was analyzed in triplicate (*n* = 3).

The strongest effects were observed against TYR and AChE, with low IC_50_ values. For *Q. rubra*, the IC_50_ values were 0.25 mg mL^−1^ for AChE and 0.14 mg mL^−1^ for TYR, while for *Q. robur* they were 0.41 mg mL^−1^ and 0.080 mg mL^−1^, respectively. The acorn fractions also effectively inhibited AMY, particularly *Q. robur*, which showed an IC_50_ value of 0.22 mg mL^−1^ compared with 0.61 mg mL^−1^ for *Q. rubra*. Inhibition of GLU was weaker, with IC_50_ values of 2.2 and 1.8 mg mL^−1^ for *Q. rubra* and *Q. robur*, respectively. In the case of PNLIP, the crude fractions showed inhibitory activity. However, a clear dose–response relationship was not observed. This could be because the crude acorn fractions did not inhibit the enzyme strongly enough, or because matrix effects altered the reaction dynamics, hindering the expression of inhibition.

The inhibitory activity against AMY and GLU is consistent with previous reports showing that different *Quercus* species are able to modulate carbohydrate-digesting enzymes.

Acorn extracts from *Q. suber*, *Q. canariensis*, *Q. coccifera* and *Q. ilex* were previously reported to inhibit α-amylase, while extracts from *Q. mongolica*, *Q. gilva*, *Q. convallata*, *Q. arizonica*, *Q. petraea*, *Q. pubescens* and *Q. robur* also showed inhibitory activity against α-amylase and/or α-glucosidase.^10,35^ Particularly strong effects were reported for *Q. coccifera* leaves and *Q. robur* bark or leaf extracts, which supports the assumption that the antidiabetic potential of oak-derived materials is closely associated with their high content of tannins and other phenolic compounds.^10,35^ In this context, the strong AMY inhibition observed for the *Q. robur* acorn fraction in the present study further confirms that acorns, similarly to leaves and bark, may be valuable sources of phenolics capable of modulating carbohydrate hydrolysis. The inhibition of acetylcholinesterase observed in the present study is also in line with previous evidence, although literature data on AChE inhibition by *Quercu*s extracts are less extensive than those concerning AMY and GLU. AChE inhibitory activity has been reported for *Q. suber* phenolic extracts and for bark extracts of *Q. petraea* and *Q. pubescens*.^14,36^

The relatively low IC_50_ values obtained for both *Q. rubra* and *Q. robur* acorn fractions indicate that acorn phenolics may also contribute to neuroprotective-related bioactivity. This effect may be associated with the presence of hydrolysable tannins and their transformation products, including gallic and ellagic acids, which can interact with enzyme proteins through hydrogen bonding, hydrophobic interactions and, in the case of larger tannins, protein-complex formation.^7,9,10,12–14^

The results obtained for TYR inhibition are particularly interesting. Previous studies on oak bark extracts reported weak or only moderate tyrosinase inhibition for some Quercus species, including *Q. cerris*, *Q. dalechampii*, *Q. robur*, *Q. petraea* and *Q. pubescens*.^14,37^ In contrast, the acorn fractions analyzed in the present study showed strong activity against TYR, especially *Q. robur*, with an IC_50_ of 0.080 mg mL^−1^. This suggests that acorns may contain a phenolic profile more favourable for TYR inhibition than some previously studied bark extracts. The high activity may be related to the abundance of hydrolysable tannins and LMW phenolic acids, which can inhibit TYR by interacting with the enzyme structure and possibly by chelating copper ions in the active site.^38^

Simulated gastric digestion modified the inhibitory activity of the extracts toward digestive enzymes (Fig. 1 and S4). A complete gastric digestion blank, processed identically to the samples but without acorn phenolic fractions, was tested in parallel in all enzyme-inhibition assays and did not show relevant inhibitory activity against AChE, TYR, PNLIP, AMY, or GLU. Under the applied experimental conditions, the observed inhibitory effects predominantly reflected the activity of compounds present in the gastric-digested acorn fractions, although the contribution of nonspecific polyphenol–protein interactions cannot be completely excluded. The most notable effect was observed for PNLIP. In the crude fractions, lipase inhibition was relatively low and did not follow a clear dose–response model. After gastric digestion, the inhibitory effect increased and became better described by a logistic dose–response relationship.

**Fig. 1:**
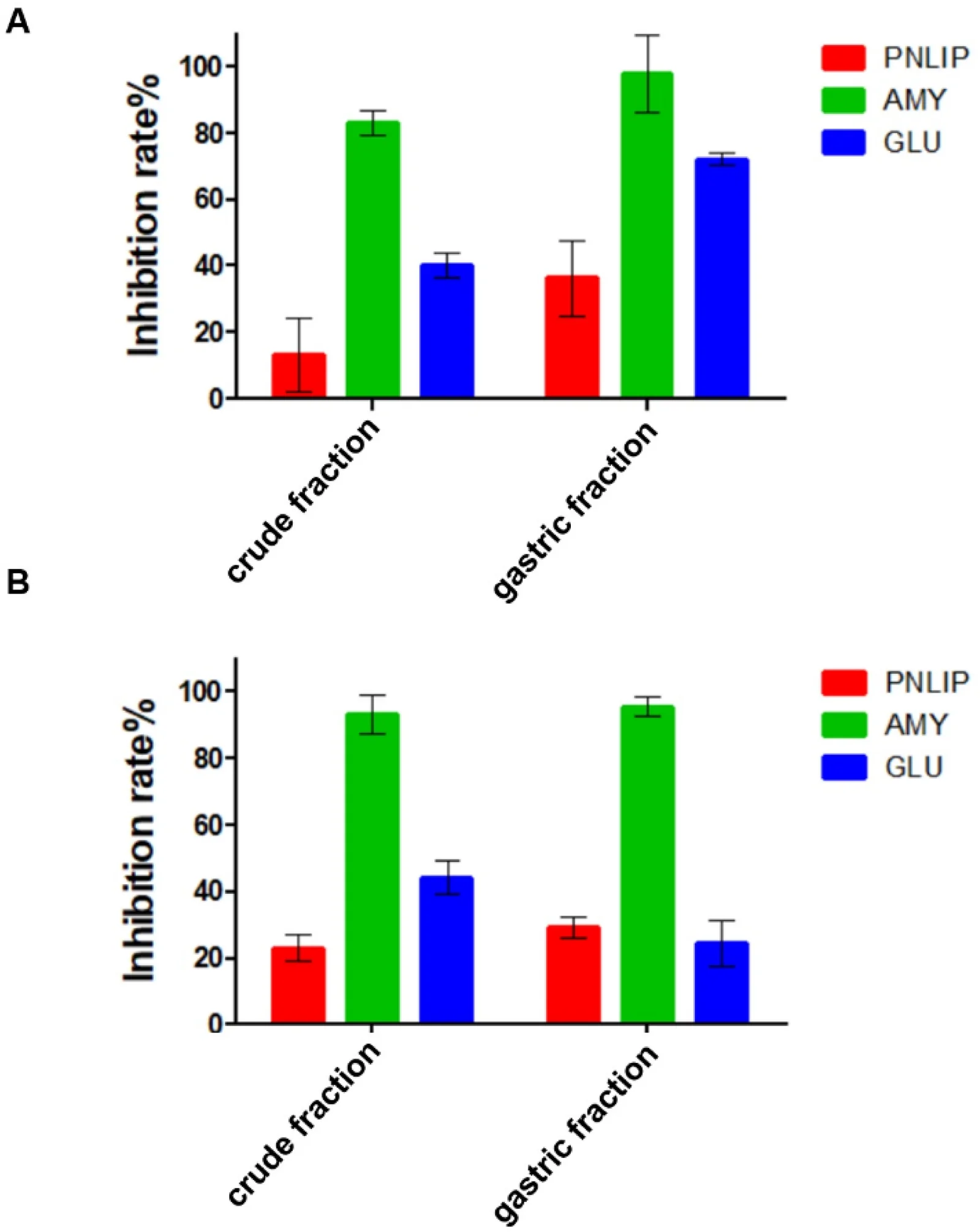
Effect of simulated gastric digestion on the inhibitory activity of *Q. rubra* (A) and *Q. robur* (B) phenolic fractions against PNLIP, AMY, and GLU. In all assays, the initial tested concentration of the acorn phenolic fractions was 4 mg mL^−1^ (*n* = 3).

This observation suggests an association between gastric digestion-induced changes in the phenolic profile and increased PNLIP inhibition under the applied assay conditions. However, the contribution of individual transformation products requires further verification. This result is especially important because, in contrast to carbohydrate-digesting enzymes, direct *in vitro* evidence for PNLIP inhibition by *Quercus* extracts is still limited. Available literature mainly provides general indications that oak-derived extracts may affect lipid metabolism or lipase-related pathways, but detailed enzyme-assay-based evidence remains scarce.^12^ Therefore, the present results provide new insight into the potential of acorn phenolics to inhibit PNLIP under *in vitro* assay conditions, particularly after gastric transformation.

In *Q. rubra*, the gastric-digested fraction showed higher inhibitory activity against PNLIP, AMY, and GLU than the corresponding crude fraction (Fig. 1). This effect may be associated with the increased content of GA, EA and selected galloylated derivatives, which can interact with enzyme proteins through hydrogen bonding and hydrophobic interactions.^39,40^ Such behaviour is consistent with the general view that gastric digestion-induced hydrolysis of tannins may convert HMW phenolics into smaller compounds that are more accessible to enzyme binding sites.^39^ At the same time, remaining GTs and ETs may still contribute to enzyme inhibition through non-specific but biologically relevant protein-binding interactions.^40^

In *Q. robur*, the influence of gastric digestion on enzyme inhibition was more selective. The inhibitory activity against AMY remained very high after gastric digestion, while PNLIP inhibition slightly increased. In contrast, inhibition of GLU decreased in the gastric fraction. This suggests that the compounds responsible for α-glucosidase inhibition in *Q. robur* may be more sensitive to gastric conditions or that their transformation products are less active toward this enzyme.

The different response of the two oak species indicates that enzyme inhibition depends not only on the total phenolic content but also on the detailed structural profile of the phenolic fraction. This is consistent with previous reports showing that the biological activity of *Quercus* samples varies depending on species, plant organ, extract composition and tannin profile.^9,10,14^

Overall, the results demonstrate that simulated gastric digestion significantly remodels the phenolic profile of *Q. rubra* and *Q. robur* acorn fractions. Although the total content of identified phenolic compounds and antioxidant activity decreased after gastric digestion, the acorn fractions retained strong biological potential. The partial degradation of ETs and GTs was accompanied by an increase in smaller phenolic acids, particularly GA and EA, which may remain active under gastric conditions. Importantly, gastric digestion enhanced or maintained the inhibitory potential of the fractions toward selected digestive enzymes, especially PNLIP and AMY. These findings suggest that acorn phenolic fractions are a promising source of bioactive phenolic compounds, which may retain or modify their antioxidant and enzyme-inhibitory activities during the initial stages of gastric digestion. However, it should be emphasized that this study focused exclusively on the simulated gastric phase of digestion. Therefore, the observed changes in phenolic composition and enzyme-inhibitory activity should be interpreted as gastric digestion-induced effects and should not be considered representative of the complete gastrointestinal fate of acorn phenolics. Subsequent exposure to intestinal pH, bile salts, and pancreatic enzymes may further transform hydrolysable tannins and low-molecular-weight phenolic compounds, potentially modifying their activity toward intestinal enzymes such as pancreatic lipase, α-amylase, and α-glucosidase. Thus, the results obtained for the gastric-digested fractions indicate potential enzyme-modulating properties under the applied *in vitro* assay conditions, but do not allow conclusions regarding their final intestinal bioactivity. Another important limitation is related to the protein-binding properties of hydrolysable tannins. These highly hydroxylated polyphenols can interact with proteins through hydrogen bonding, hydrophobic interactions, and possible precipitation or aggregation phenomena. Therefore, the inhibitory effects observed for acorn phenolic fractions may result from both specific interactions with enzyme binding sites and non-specific tannin–protein interactions. Finally, the present study demonstrates associations between gastric digestion-induced changes in the phenolic profile and altered enzyme-inhibitory activity. Further studies using purified compounds and defined mixtures at concentrations corresponding to those present in gastric-digested fractions are needed to confirm the specific roles of gallic acid, ellagic acid, and representative hydrolysable tannins in the observed inhibitory effects.

Taken together, the present results expand the existing knowledge on the enzyme-inhibitory activity of *Quercus*-derived products. Furthermore, to the best of our knowledge, this is the first study to demonstrate the inhibitory activity of the *Q. rubra* phenolic fraction against the tested enzymes, particularly AChE and TYR. Previous studies have mainly focused on bark, leaves or acorns of other oak species and have most consistently reported inhibition of AMY and GLU.^7,9,10,14,35^ The current study shows that acorns of *Q. rubra* and *Q. robur* also exhibit multifunctional activity, including inhibition of AChE, TYR, AMY, GLU, and PNLIP. Moreover, the results highlight that the biological activity of oak acorn phenolic fractions appears to be associated not only with their native composition but also with gastric digestion-induced changes in their phenolic profile.

### 2.4. Molecular docking analysis

Molecular docking analysis was performed to gain preliminary insight into the possible interactions between the major phenolic compounds identified in phenolic-rich fractions of *Q. rubra* and *Q. robur* acorns and the enzymes evaluated in the experimental assays. This approach was used to predict possible ligand–enzyme interaction patterns within enzyme binding pockets, including hydrogen bonding, hydrophobic contacts, and π-related interactions. In this study, docking scores were calculated for selected acorn phenolics against AChE (4EY6), TYR (2Y9W), PNLIP (1ETH), AMY (3BAY), and GLU (5NN8), as summarized in Table 5.

**Table 5:** *In silico* docking scores of selected phenolic compounds from *Q. rubra* and *Q. robur* acorns with acetylcholinesterase, pancreatic lipase, α-amylase, tyrosinase, and glucosidase^a^

No.	Compound name	Pubchem ID	Affinity (kcal mol^−1^)				
			AChE (4EY6)	TYR (2Y9W)	PNLIP (1ETH)	AMY (3BAY)	GLU (5NN8)
1	Ellagic acid	CID 5281855	−10.0	−6.7	−9.2	−8.5	−6.2
2	Gallic acid	CID 370	−6.4	−5.5	−6.3	−6.2	−5.5
3	Cretanin	Not available in PubChem (N/A)	−9.4	−7,1	−8.4	−8.5	−7.4
4	Oenothein B	CID 16129800	−7.2	−7,2	−7.5	−8.6	−11.3
5	Vascalagin	CID 168165	−8.1	−8.1	−8.7	−7.4	−4.9
6	Castalagin	CID 12302513	−7.3	−8.3	−9.3	−8.1	−1.8
7	Rugosin E	CID 16129738	−7.4	−7.8	−7.5	−9.0	−7.6
8	Tellimagrandin II	CID 151590	−10.6	−8.6	−9.1	−9.4	−7.3
9	Galloylglucose	CID 124375	−8.9	−6,8	−8.3	−7.6	−7.0
10	Vescavaloneinic acid	SID 319498283	−8.5	−8.0	−7.1	−10.2	−8.1
11	Pentagalloylglucose	CID 65238	−9.7	−7.5	−8.2	−9.6	−9.1
12	Tyrosine (control)	CID 6057	—	−5.4	—	—	—
13	Acarbose (control)	3BAY form PDB	—	—	—	−8.4	−7.4
14	Orlistat (control)	CID 303401	—	—	−7.8	—	—
15	Acetylcholine (control)	CID 187	−6.6	—	—	—	—

a —: no data; AChE – acetylcholine esterase, PLIP – pancreatic lipase, AMY – α-amylase, TYR – tyrosinase, GLU – glucosidase.

The AChE active pocket has two main regions (Fig. 2). Outer with a narrow cavity – gorge – which leads to the catalytic centre. The entrance of the gorge is formed by the peripheral anionic site (PAS), which comprises aromatic residues including Tyr72, Asp74, Tyr124, Trp286, and Tyr341.^41^

**Fig. 2:**
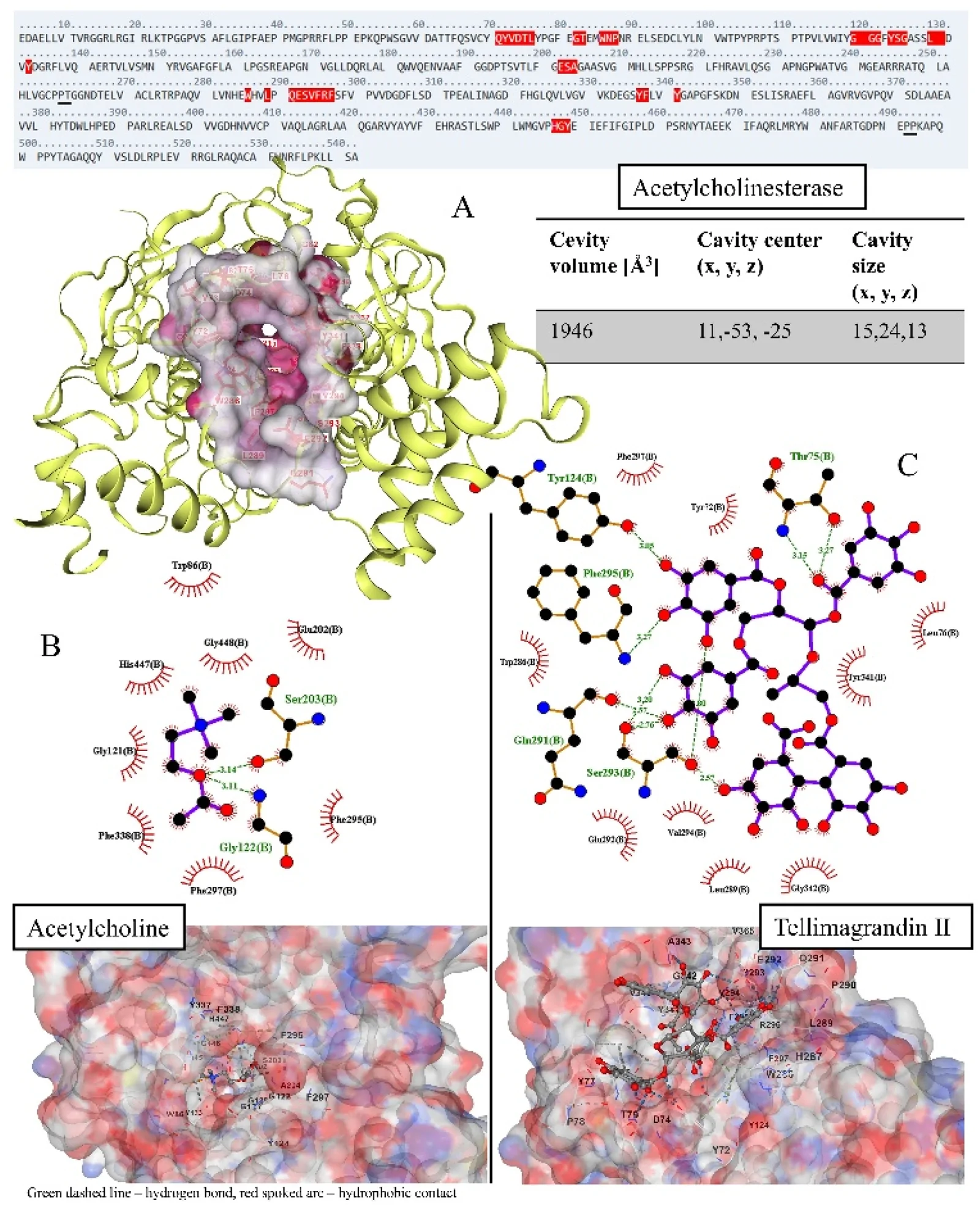
Acetylcholinesterase (4EY6) catalytic cavity, cavity volume, and key amino acid residues forming the catalytic pocket (A), along with docking results for the natural substrate, acetylcholine (B), and the compound with the highest predicted affinity for AChE, tellimagrandin II (C), presented as a 2D interaction scheme and 3D visualization.

These residues are responsible for the initial recognition and orientation of ligands through hydrogen bonding, π–π stacking, and hydrophobic interactions. Deeper within the gorge lies the catalytic active site (CAS), consisting of the catalytic triad Ser203–Glu334–His447 and the catalytic anionic subsite centred on Trp86 (Fig. 2A). Molecular docking analysis against AChE yielded docking scores ranging from −6.4 to −10.6 kcal mol^−1^ (Table 5). Tellimagrandin II yielded the most favourable docking score (−10.6 kcal mol^−1^), followed by EA (−10.0 kcal mol^−1^), pentagalloylglucose (−9.7 kcal mol^−1^), and cretanin (−9.4 kcal mol^−1^), whereas GA displayed the least favourable score (−6.4 kcal mol^−1^). The obtained docking scores were within a range similar to values reported for established AChE inhibitors, including quercetin (−10.7 kcal mol^−1^), donepezil (−10.5 kcal mol^−1^), galantamine (−10.2 kcal mol^−1^),^42^ and GA (−6.5 kcal mol^−1^). However, these values should not be interpreted as quantitatively comparable experimental binding affinities. Analysis of ligand–protein interactions revealed a conserved binding pattern within the AChE active-site gorge. The most frequently appear residues included Tyr72, Asp74, Tyr124, Trp286, Tyr341, Trp86, Phe295, Phe297, Tyr337, Phe338, and His447 (Table S1).

These residues constitute the PAS and the CAS region of AChE, both of which play essential roles in substrate recognition and enzyme inhibition. Structural studies have demonstrated that simultaneous interactions with PAS- and CAS-associated residues increase ligand residence time and contribute to effective enzyme inhibition.^42^ Tellimagrandin II formed the most extensive interaction network (Fig. 2C), establishing multiple hydrogen-bonding and aromatic interactions distributed throughout the active-site gorge, particularly with Tyr124, Trp286, Tyr341, and the Phe295–Phe297 aromatic cluster. Such multipoint interactions enable simultaneous occupation of both peripheral and deeper regions of the binding pocket, a mechanism described for high-affinity AChE inhibitors such as quercetin.^42^

The catalytic binding pocket of TYR is located within a relatively compact and well-defined active-site cavity that accommodates both monophenolic substrates and larger polyphenolic inhibitors through a combination of polar and hydrophobic interactions (Fig. 3A). Structurally, the pocket is organized around a conserved set of residues, including His178, Glu173, Gln159, and Gln196, which contribute to ligand stabilization through hydrogen bonding and electrostatic interactions, while His178 often plays a central role in anchoring ligands within the catalytic region. This arrangement is consistent with the binuclear copper active-site architecture characteristic of TYRs, where substrate binding occurs in proximity to the copper-coordinating histidine environment.^43,44^ Surrounding this catalytic core, additional residues such as Arg38, Gln41, Gln44, Ala45, Tyr179, and Phe181 form a secondary interaction shell that contributes to ligand positioning and stabilization through van der Waals contacts, hydrogen bonding, and π-related interactions. Molecular docking analysis revealed docking scores ranging from −5.5 to −8.6 kcal mol^−1^, using tyrosine as the reference ligand (−5.4 kcal mol^−1^). Overall, phenolic compounds yielded more favourable docking scores compared to the reference substrate, with a relatively narrower score distribution than observed for other enzymes analysed in this study. The most favourable predicted score was observed for tellimagrandin II (−8.6 kcal mol^−1^), followed by castalagin (−8.3 kcal mol^−1^), vescalagin (−8.1 kcal mol^−1^), and oenothein B (−7.2 kcal mol^−1^), indicating that ET-type structures provide the most favourable predicted interaction profiles within the tyrosinase binding pocket.

**Fig. 3:**
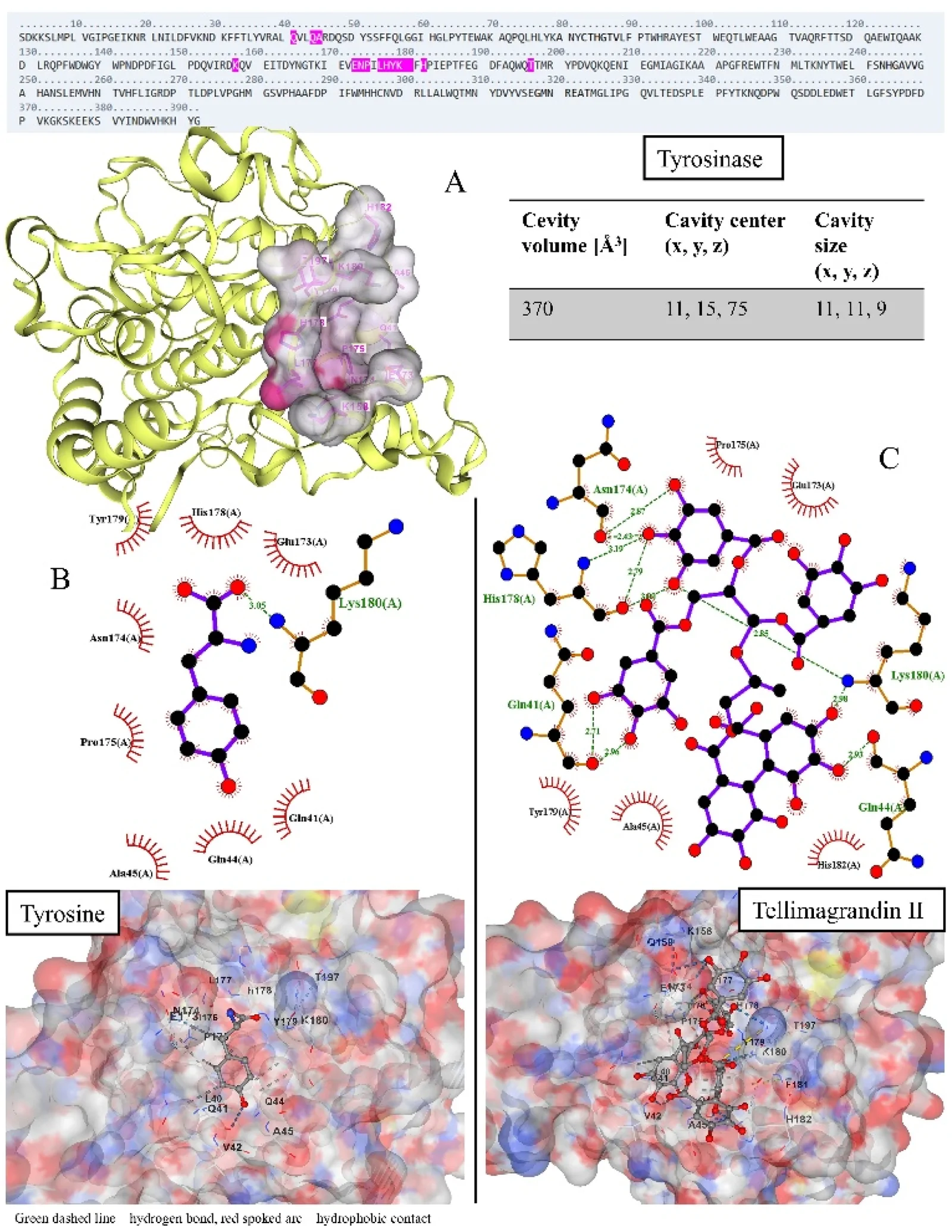
Tyrosinase (2Y9W) catalytic cavity, cavity volume, and key amino acid residues forming the catalytic pocket (A), along with docking results for the natural substrate, tyrosine (B), and the compound with the highest predicted affinity for TYR, tellimagrandin II (C), presented as a 2D interaction scheme and 3D visualization.

In contrast, GA showed the least favourable docking score (−5.5 kcal mol^−1^), only marginally stronger than tyrosine (Table 5). Across all complexes in the present study, a conserved interaction region was identified within the TYR binding pocket, primarily involving residues Gln159, Glu173, His178, and Gln196.

Among these, His178 consistently acted as a central anchoring residue, while Gln159 and Gln196 contributed to hydrogen bond stabilization, and Glu173 provided electrostatic complementarity. This interaction pattern is consistent with reported binding modes, where TYR inhibition is governed by cooperative interactions within a defined catalytic pocket rather than single-residue binding events.^45^ In addition to this catalytic core, larger polyphenolic structures engaged in an extended interaction network involving residues such as Arg38, Gln41, Val42, Gln44, Ala45, and the flexible loop region spanning residues 170–184. Aromatic residues, including Tyr179 and Phe181, further stabilized ligand binding through π-related interactions and van der Waals forces, particularly in HMW tannins. Tellimagrandin II exhibited the most extensive interaction profile, engaging in key catalytic residues while simultaneously forming multiple contacts within the surrounding loop and entrance regions of the binding pocket (Fig. 3C). Similarly, castalagin and vescalagin demonstrate broad interaction networks characterized by dense hydrogen bonding and auxiliary hydrophobic stabilization. Oenothein B displayed a slightly less compact but still extensive binding mode, relying on conformational flexibility to maintain multi-point interactions across the catalytic and peripheral regions. In contrast, GA exhibited a restricted binding mode, primarily involving surface-exposed residues such as Gln41, Gln44, and His178, without deep penetration into the catalytic pocket. This behaviour is not new, because small phenolic acids predominantly form limited hydrogen bond networks and exhibit weaker overall stabilization within tyrosinase.^45^ reference ligand tyrosine showed minimal interaction within the binding pocket (Fig. 3B), engaging mainly peripheral residues and lacking extensive stabilization compared to polyphenolic compounds.

The binding pocket of PNLIP is characterized by a large, shallow, and amphipathic cavity lined with both hydrophobic and polar residues, enabling accommodation of structurally diverse ligands.^46^ The active-site region forms a broad interfacial binding surface rather than a narrow catalytic channel, with key residues such as His152, Leu154, Ala179, Asp206, Ala207, Ala208, Phe216, Cys238, and Gln239 (Fig. 4A) contributing to ligand stabilization through combined hydrophobic interactions, hydrogen bonding, and electrostatic contacts.^47^ The entrance of the pocket is relatively flexible and allows partial adaptation to ligand size and geometry, while the deeper region provides a more hydrophobic environment that supports stable anchoring of bulky inhibitors. This structural organization enables multi-point binding across the cavity and is a key determinant of ligand interactions in pancreatic lipase inhibition studies.^48^ Molecular docking analysis against PNLIP yielded docking scores ranging from −6.3 to −9.3 kcal mol^−1^, using orlistat as the reference inhibitor (−7.8 kcal mol^−1^) (Table 5). Overall, most phenolic structures exhibited docking scores comparable to or exceeding the reference compound, with castalagin (−9.3 kcal mol^−1^) and EA (−9.2 kcal mol^−1^) showing the most favourable predicted scores, followed by tellimagrandin II (−9.1 kcal mol^−1^) and vescalagin (−8.7 kcal mol^−1^). In contrast, GA displayed the least favourable docking score (−6.3 kcal mol^−1^), indicating the predicted interaction profile was influenced by ligand size, conformation, and the spatial arrangement of functional groups within the binding cavity. A common mechanistic feature observed for compounds yielding more favourable docking scores was their ability to establish multi-point interactions spanning both the entrance and the deep catalytic cavity. Castalagin, which exhibited the most favourable docking score (Fig. 4C), formed extensive interactions with His152, Ala179, Asp206, Phe216, Cys238, and Gln239.

**Fig. 4:**
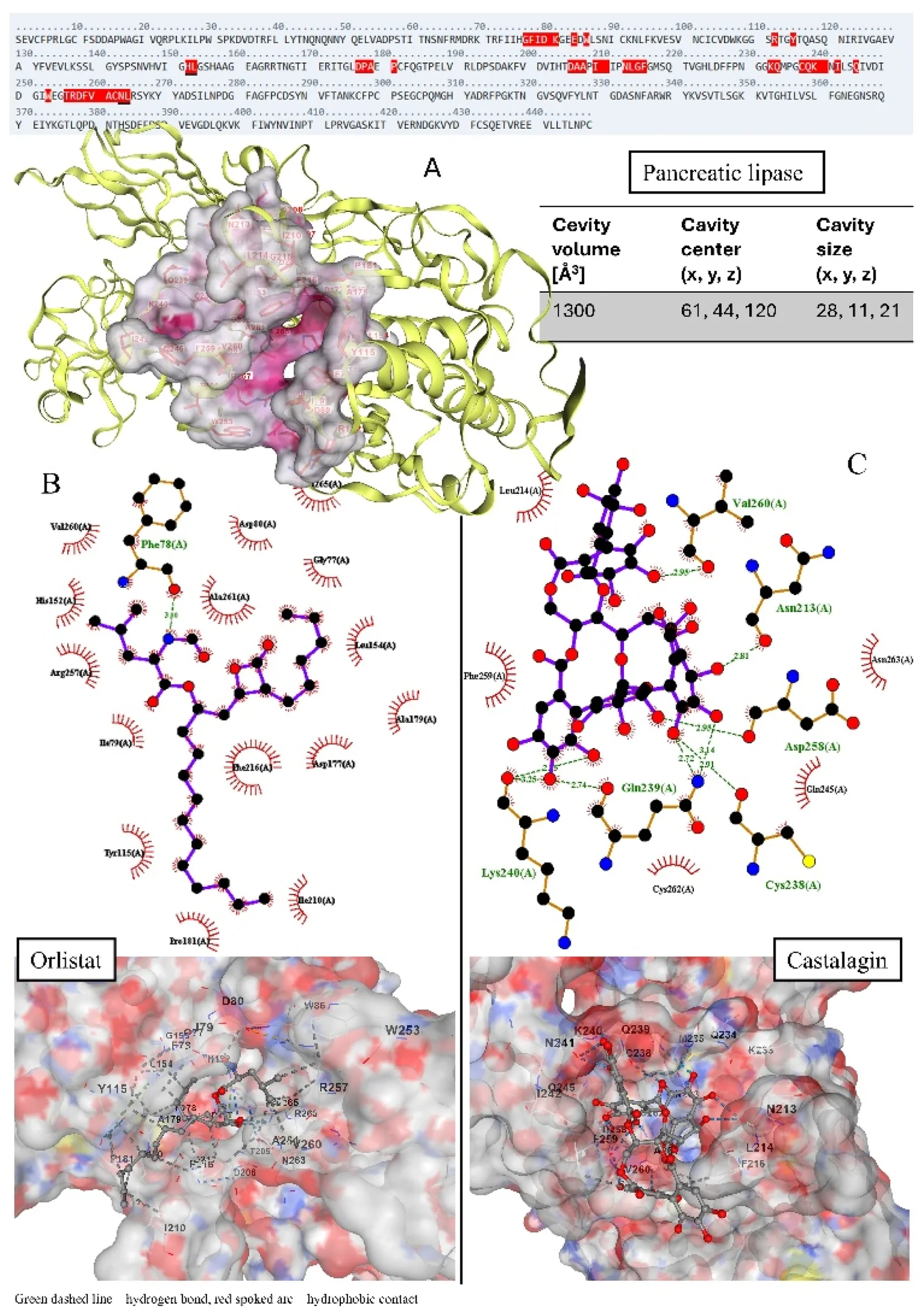
Pancreatic lipase (1ETH) catalytic cavity, cavity volume, and key amino acid residues forming the catalytic pocket (A), along with docking results for the reference substrate, orlistat (B), and the compound with the highest predicted affinity for PLIP, castalagin (C), presented as a 2D interaction scheme and 3D visualization.

This broad interaction profile indicates effective occupation of the central cavity and stabilization across both hydrophobic and polar regions of the active site. Such binding behaviour is consistent with reports indicating that this tannin exert lipase inhibition through distributed surface anchoring and multi-site interaction networks rather than single-point binding.^49^ EA and tellimagrandin II displayed similar binding behaviour, engaging key residues within the same central cavity, including Asp206, Ala207, Ala208, and Phe216, suggesting flexible accommodation within the active site. Vescalagin showed a comparable but slightly less extensive interaction pattern, while still maintaining stable contacts with the catalytic cavity residues. In contrast, GA exhibited limited penetration into the catalytic cavity and primarily interacted with residues located at or near the entrance region, including Phe78, Ile79, Tyr115, His152, and Ala179. The reduced number of contacts and lack of deep cavity engagement likely explain its significantly lower docking score compared to larger polyphenolic structures.

Smaller phenolic acids possess restricted spatial adaptability, limiting their ability to form stable multi-point interactions within pancreatic lipase.^49^ The reference inhibitor orlistat reproduced the expected binding profile within the PNLIP active site, engaging key residues including His152, Asp206, and the hydrophobic pocket formed by Ala207, Ala208, Phe216, and Cys238 (Fig. 4B). However, several phenolic compounds demonstrated comparable or even more extensive residue coverage, suggesting that non-covalent multi-point interactions may contribute significantly to inhibitory potential.

The catalytic binding region of AMY is organized as a well-defined and elongated active-site cleft that accommodates carbohydrate substrates through multiple cooperative interactions. This cleft is centred around the conserved catalytic triad Asp197, Glu233, and Asp300, which are essential for substrate recognition and stabilization of the transition state of α-amylase.^50^ Surrounding the catalytic core, a network of polar and hydrophobic residues, including Tyr62, Gln63, His101, Tyr151, Leu162, Arg195, Ala198, Ser199, Lys200, and His201, contributes to substrate positioning through hydrogen bonding and van der Waals interactions, forming an extended carbohydrate-binding groove (Fig. 5A). Molecular docking analysis revealed docking scores ranging from −6.2 to −10.2 kcal mol^−1^, using acarbose as the reference inhibitor (−8.4 kcal mol^−1^) (Table 5). The most favourable docking score was observed for vescavaloninic acid (−10.2 kcal mol^−1^) followed by pentagalloylglucose (−9.6 kcal mol^−1^) and tellimagrandin II (−9.4 kcal mol^−1^), all exceeding the docking score of acarbose. It is important, to not take these differences as evidence of higher experimental inhibitory potency or binding affinity. EA and cretanin also showed high docking scores (both −8.5 kcal mol^−1^), whereas GA exhibited the lowest (−6.2 kcal mol^−1^). These findings align with reports demonstrating that phenolic constituents and galloylglucose derivatives can effectively interact with carbohydrate-active enzymes through non-covalent mechanisms, including hydrogen bonding, hydrophobic interactions, and π-related contacts, resulting in stable enzyme–ligand complexes.^51^ A common mechanistic feature observed for compounds yielding more favourable docking scores was their ability to establish extensive multi-point interactions spanning both the catalytic core and peripheral regions of the binding cleft. Acarbose engaged all three catalytic residues as well as key substrate-binding residues, confirming its role as a competitive inhibitor occupying the catalytic channel^51^ (Fig. 5B).

**Fig. 5:**
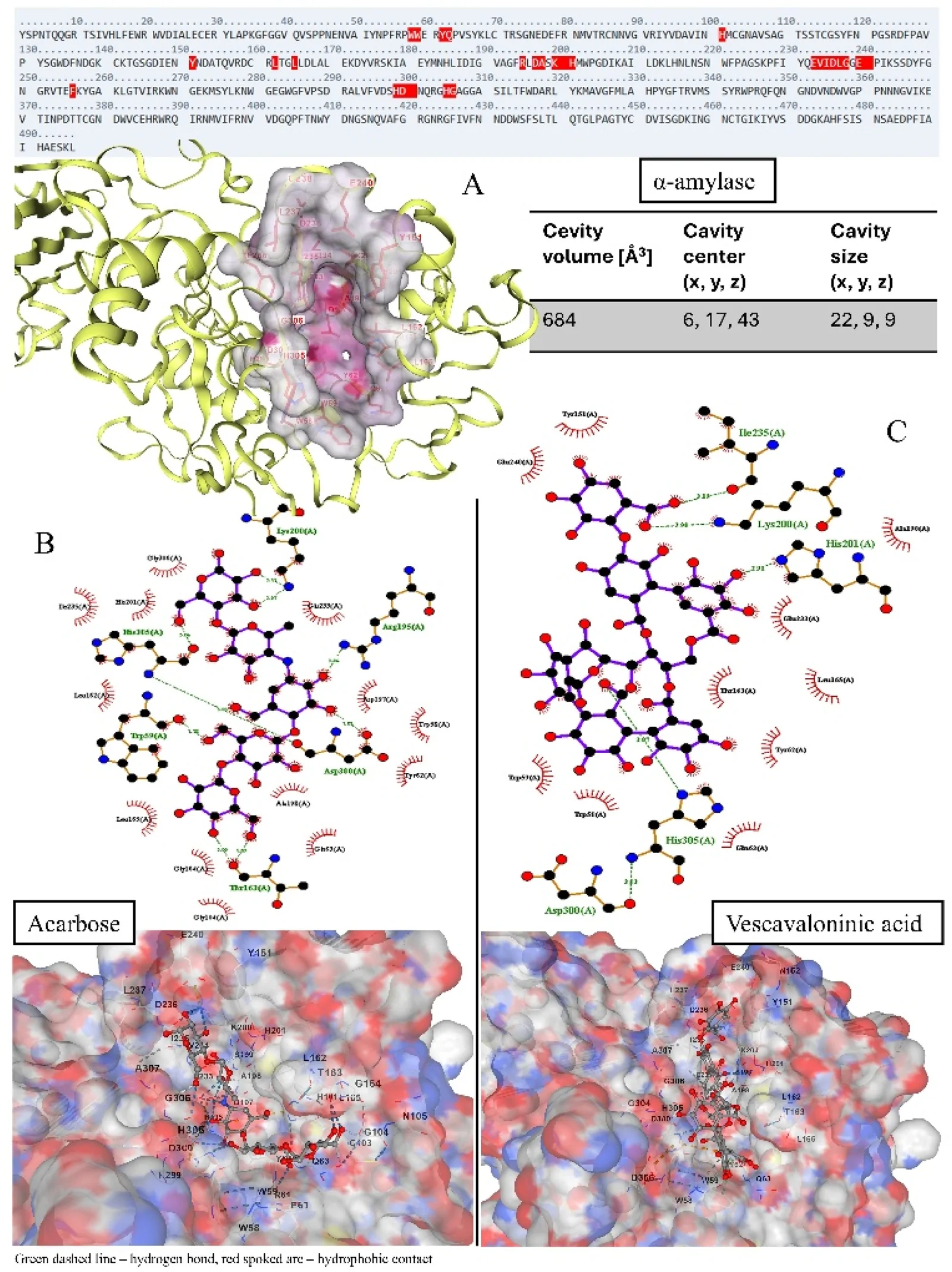
α-Amylase (3BAY) catalytic cavity, cavity volume, and key amino acid residues forming the catalytic pocket (A), along with docking results for the reference substrate, acarbose (B), and the compound with the highest predicted affinity for AMY, vescavaloninic acid (C), presented as a 2D interaction scheme and 3D visualization.

Notably, several phenolic compounds in the present study, particularly vescavaloninic acid (Fig. 5C), pentagalloylglucose, and tellimagrandin II, exhibited comparable or even more extensive residue coverage than acarbose (Table S1), suggesting that non-covalent multi-site binding may effectively compete with classical carbohydrate-mimetic inhibition mechanisms. In contrast, GA displayed a restricted binding mode, mainly involving entrance-region residues such as Trp58, Trp59, and Tyr62, with limited penetration into the catalytic core. Similar observations have been reported in previous studies, where small phenolic acids demonstrated weaker stabilization within AMY due to their limited spatial adaptability and reduced capacity for multi-point interactions.^51^

The GLU active site forms a narrow substrate-binding cavity within the catalytic (β/α)8 domain, shaped by conserved residues involved in substrate and inhibitor recognition. The pocket contains catalytic acidic residues (Asp518 and Asp616) and multiple aromatic residues, including Trp376, Trp481, Trp516, Trp613, and Phe649, which contribute to ligand stabilization through hydrogen bonding, hydrophobic contacts, and aromatic interactions (Fig. 6A). This extended interaction network enables accommodation of structurally diverse inhibitors, including carbohydrate mimetics and polyphenolic compounds.^52^ Molecular docking analysis revealed docking scores ranging from −1.8 to −11.3 kcal mol^−1^, with acarbose used as the reference inhibitor (−7.4 kcal mol^−1^) (Table 5). Among all tested compounds, oenothein B exhibit highest docking score (−11.3 kcal mol^−1^), followed by pentagalloylglucose (−9.1 kcal mol^−1^) and vescavaloninic acid (−8.1 kcal mol^−1^). Several ETs, including rugosin E, tellimagrandin II, and cretanin, showed docking scores comparable to acarbose (Fig. 6B), whereas castalagin and vescalagin displayed lower score, indicating that binding efficiency depends on ligand orientation and accessibility of functional groups rather than molecular size alone. Oenothein B showed the most favourable interaction profile within the GLU binding pocket, engaging residues involved in substrate and inhibitor recognition, including Asp282, Trp376, Trp481, Met519, Ser523, Phe525, Asp616, Phe649, and the His674–Leu678 region (Fig. 6C). The ligand was positioned within the extended binding cavity and stabilized by multiple aromatic, hydrophobic, and hydrogen-bond interactions. The involvement of conserved aromatic residues, particularly Trp376, Trp481, and Phe649, suggests that π-related and hydrophobic interactions play an important role in accommodating the highly galloylated structure of oenothein B. Interaction with Asp616, a residue associated with catalytic proton transfer in GH31 α-glucosidases, further indicates that the ligand occupies a functionally relevant region of the active-site cavity. Pentagalloylglucose also demonstrated high docking score, mainly through interactions with conserved aromatic residues (Trp376, Trp481, Trp516, and Trp613), supporting the importance of extensive aromatic contacts for stabilization of polyphenolic ligands. Other ETs exhibited similar interaction patterns involving key residues of the binding cavity, suggesting that these compounds can adapt to the GLU active site through multi-point anchoring.

**Fig. 6:**
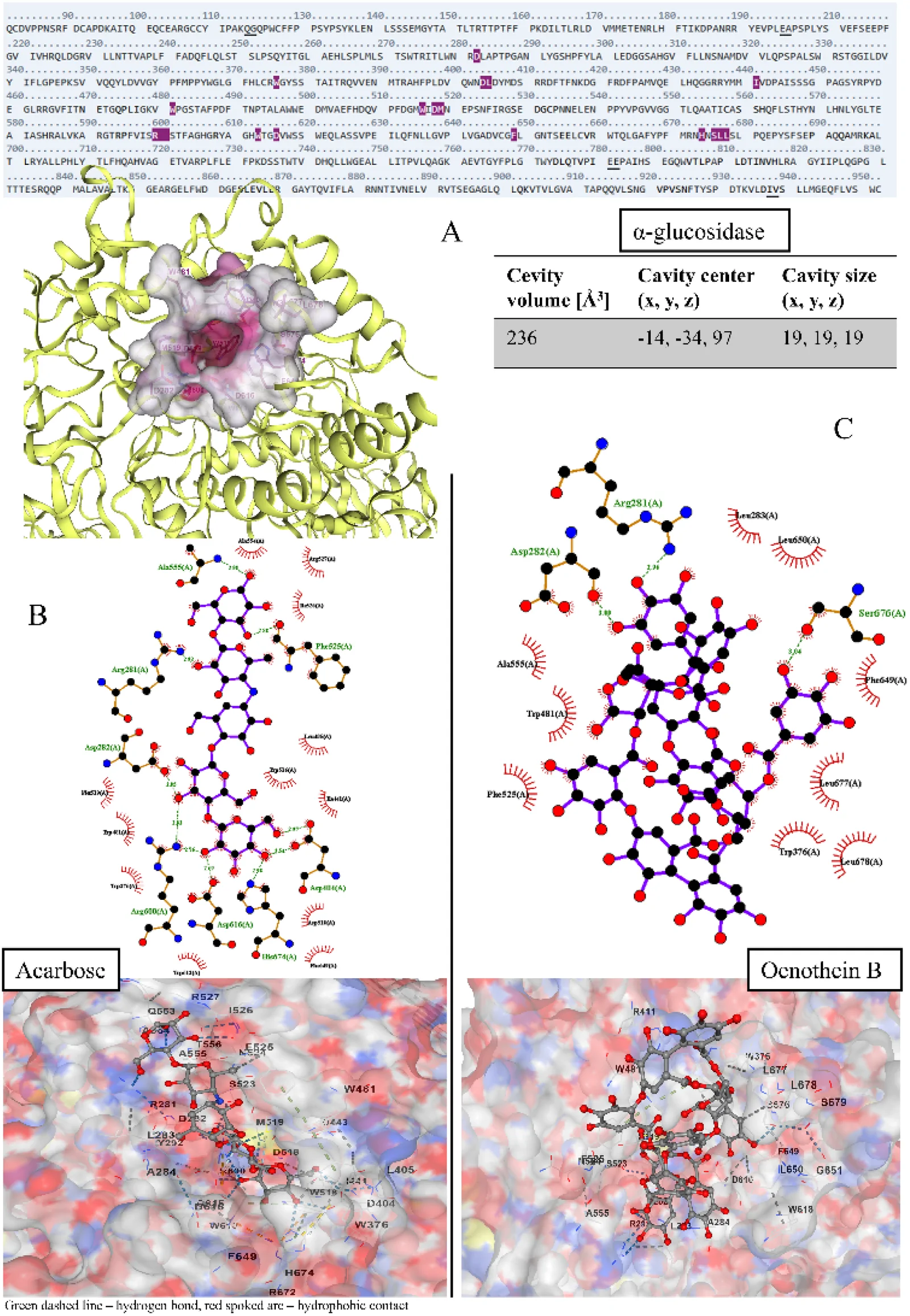
α-Glucosidase (5NN8) catalytic cavity, cavity volume, and key amino acid residues forming the catalytic pocket (A), along with docking results for the natural substrate, acarbose (B), and the compound with the highest predicted affinity for GLU, oenothein B (C), presented as a 2D interaction scheme and 3D visualization.

In contrast, smaller phenolic compounds showed more limited stabilization within the pocket. GA and EA displayed less favourable docking scores (−5.5 and −6.2 kcal mol^−1^, respectively), likely due to fewer simultaneous contacts compared with larger galloylated structures. Notably, castalagin showed a very unfavourable docking score despite its high molecular size, further demonstrating that effective occupation of the GLU cavity requires appropriate ligand conformation and positioning of interacting groups.

Across all enzymes studied, docking revealed a general tendency for more favourable docking scores to be associated with the ability of ligands to establish multiple interactions within enzyme-specific binding pockets, including hydrogen bonds, hydrophobic contacts, and π-interactions. Although HMW phenolic compounds often showed high docking score, this relationship was not universal. Several larger molecules exhibited less favourable scores, indicating that molecular mass alone is not the main determinant of binding. Instead, ligand conformation, spatial arrangement of functional groups, and accessibility of interacting moieties appear to play key roles in complex stabilization. Despite its usefulness, molecular docking has important limitations. It represents a simplified model that usually treats the receptor as a relatively rigid structure, whereas proteins are dynamic and may undergo conformational changes during ligand binding. Docking simulations also do not fully account for solvent effects or other physiological factors. These limitations are particularly relevant for the larger tannins investigated in the present study, which possess high conformational complexity and multiple rotatable or constrained structural elements. Therefore, the predicted binding modes and scores should be interpreted as indicative of potential interactions rather than definitive evidence of binding mechanisms or quantitative binding affinities. Accordingly, docking scores were used primarily to compare predicted interaction patterns within the applied computational workflow and were not interpreted as quantitative measures of binding affinity. The obtained *in silico* results should be validated by complementary experimental studies, including targeted *in vitro* or *in vivo* assays, to confirm the proposed mechanisms and biological activity.

### 2.5. *In silico* prediction of ADME parameters and toxicity profiles

*In silico* prediction of ADME parameters and toxicity profiles was performed to preliminarily assess the potential bioavailability, pharmacokinetic behaviour, and safety of the major phenolic compounds identified in the acorns of *Q. rubra* and *Q. robur*. The analysis covered both LMW products of tannin hydrolysis, such as GA and EA, as well as compounds with significantly higher molecular weights, representing GTs and ETs, including pentagalloylglucose, tetragalloylglucose, punicalagin, casuarictin, tellimagrandin II, vescalagin/castalagin, oenothein B, and rugosin E (Table S2).

The most favourable absorption profile was predicted for smaller phenolic compounds, particularly EA and GA. EA, despite its moderately low water solubility, was characterized by a positive Caco-2 permeability value and high predicted intestinal absorption. Although *in silico* analyses suggested favourable intestinal permeability and high predicted absorption of EA, these predictions should be interpreted with caution, as numerous experimental and clinical studies indicate that its bioavailability following oral administration is very low due to poor water solubility, limited intestinal absorption, and rapid metabolism.^53,54^ After ingestion, EA is rapidly converted into glucuronidated and methylated metabolites, while a significant portion reaches the colon and is converted by the intestinal microbiota into urolithins, which exhibit significantly higher bioavailability and may be the primary factor responsible for the observed biological effects. Nevertheless, it can be supposed that EA remains in the gastrointestinal tract long enough to interact directly with digestive enzymes, such as GLU, AMY and PNLIP,^53^ before being converted by microbes into urolithins, due to its poor solubility, limited permeability and low intestinal absorption.^53^ GA, on the other hand, exhibited moderate predicted intestinal absorption and a relatively simple transport profile, with no indication of acting as a substrate or inhibitor of *P*-glycoprotein. This implies that its release during the hydrolysis of GTs may increase the pool of biologically more accessible compounds compared to their HMW precursors.

For compounds with higher molecular weights, such as ETs and GTs, the predicted absorption profile was less favourable (Table S2). They were characterized by high molecular weight, a large number of hydrogen-bond donors and acceptors, and an extensive polar surface area,^55^ which limits passive diffusion through biological membranes.^56^ The predicted Caco-2 permeability values for HMW tannins, including oenothein B, rugosin E, punicalagin, casuarictin, tellimagrandin II, and vescalagin/castalagin, were low or very low, suggesting limited direct epithelial passage in their unchanged form. This is consistent with previous Caco-2 studies indicating that ETs and EA derivatives may show low transcellular transport and strong cellular or macromolecular binding, which can limit their apparent permeability.^57^ Thus, the biological activity of these compounds may occur mainly locally in the gastrointestinal lumen, through interactions with digestive enzymes, proteins, metal ions, or food matrix components. At the same time, some *in silico* studies have predicted moderate to high intestinal absorption for selected ETs, including castalagin and vescalagin, despite only medium Caco-2 permeability and limited or moderate BBB penetration.^58^ This discrepancy highlights the need for cautious interpretation of pkCSM predictions for large phenolic compounds, which may fall outside the optimal applicability domain of predictive models. From a biological perspective, it is more likely that a substantial part of these compounds undergoes hydrolysis, chemical transformation, or microbial metabolism in the gastrointestinal tract, while their systemic effects are mainly mediated by smaller metabolites, such as GA, EA, and urolithin derivatives.^53^

Distribution analysis showed that the tested compounds have limited ability to cross the blood–brain barrier. Particularly low values of BBB permeability and CNS permeability were predicted for HMW tannins, such as oenothein B and rugosin E. This suggests that these compounds likely do not penetrate the central nervous system to a significant extent in their unchanged form. The potential neuroprotective effects of acorn phenolic-rich fractions may instead result from indirect mechanisms, such as reducing oxidative stress and inflammation or the activity of smaller metabolites formed during digestion.^59,60^ EA and GA also showed limited predicted penetration into the CNS, although their parameters were more favourable than those of major tannins.

The metabolic profile indicated that most of the analysed compounds were not predicted to be substrates of CYP2D6 or CYP3A4 and did not exhibit the ability to inhibit the major cytochrome P450 isoenzymes. This is favourable from a safety perspective, as it suggests a low risk of metabolic interactions.^61,62^

In terms of excretion, clear differences were observed between small phenolic compounds and large tannins. Gallic and ellagic acids exhibited positive predicted clearance values, which may suggest their more efficient elimination from the body. Many HMW tannins, however, were characterized by low or negative predicted clearance and were identified as substrates of the OCT2 transporter.^63^ It should be noted, however, that for large phenolic compounds these predictions are indicative only.

The toxicity profile assessed using the pkCSM method was generally favourable. Most of the analysed compounds showed no predicted AMES toxicity or hepatotoxicity, nor did they exhibit skin sensitization.^61^

A preliminary safety assessment of the major phenolic compounds identified in *Q. rubra* and *Q. robur* acorn fractions was performed using an additional *in silico* toxicity analysis on the ProTox-3.0 platform. From the perspective of the scope of this study, the most relevant predictions concerned acute oral toxicity, hepatotoxicity, mutagenicity, cytotoxicity, potential organ effects, and possible metabolic interactions. Most of the analysed ETs and GTs, including vescavaloninic acid, casuarictin, oenothein B, pentagalloylglucose, punicalagin, rugosin E, tellimagrandin II, tetragalloylglucose, and vescalagin/castalagin, were classified as toxicity class 5 (Table S2). The predicted LD_50_ values were mostly in the range of approximately 2170 to 3700 mg kg^−1^. These results indicate a relatively low acute oral toxicity potential for the tested HMW tannins. Cretanin was also classified as Class 5, with the highest predicted LD_50_ value of 5000 mg kg^−1^, suggesting a favourable acute toxicity profile for this compound. A slightly different profile was obtained for the smaller hydrolysis products of tannins, *i.e.*, GA and EA. Both compounds were assigned to toxicity class 4, with predicted LD_50_ values of 2000 mg kg^−1^ for GA and 2991 mg kg^−1^ for ellagic acid, respectively. These data do not necessarily indicate high toxicity, but they do suggest that, in the ProTox-3.0 model, these compounds were assessed as slightly less favourable in terms of acute oral toxicity than most HMW ETs and GTs. The most significant finding from the perspective of the potential use of phenolic-rich acorn fractions as functional food ingredients is that most of the analysed compounds were predicted to lack hepatotoxicity, neurotoxicity, mutagenicity, and cytotoxicity. This is particularly important because acorns are rich in phenolic compounds with high biological activity, and their potential use requires demonstrating that this activity is not associated with general cellular toxicity. However, certain findings were observed that require cautious interpretation, in particular the potential nephrotoxicity and immunotoxicity of selected HMW ETs, as well as their effects on certain cellular stress pathways or CYP isoenzymes.

Therefore, the results of these *in silico* analyses should be considered preliminary and in need of validation through appropriate biological models. This is consistent with the findings of other researchers,^64^ who emphasized that tools such as ProTox 3.0 and ADMET-AI are useful for rapid toxicity screening. However, they noted that the predictions of these tools may differ from experimental results due to model limitations, training dataset bias, and insufficient consideration of the complex metabolic transformations of plant phenolic compounds.

## 3 Conclusions

This study demonstrated that phenolic-rich fractions from *Q. rubra* and *Q. robur* acorns are valuable sources of bioactive polyphenols, dominated by hydrolysable tannins, mainly ETs and GTs, together with gallic and ellagic acid derivatives. Simulated gastric digestion remodelled the phenolic profile by reducing selected HMW tannins and increasing low-molecular-weight phenolics, particularly gallic and ellagic acids. Although antioxidant activity decreased after digestion, especially in the EPR assay, the fractions retained considerable antioxidant potential, indicating the important contribution of structurally intact ETs to radical-scavenging activity.

Both the crude fractions and those subjected to gastric digestion exhibited multifunctional enzyme-inhibitory activity, particularly against AChE, TYR, and AMY, while inhibition of GLU was weaker. Following simulated gastric digestion, the resulting fractions maintained or even enhanced *in vitro* inhibitory activity against PNLIP and AMY, suggesting that gastric-stage changes in phenolic composition may modify enzyme interactions. These results indicate that the biological activity of phenolic fractions from acorns depends not only on the total phenolic content but also on the structural composition and digestion-induced transformations of individual phenolic compounds.

*In silico* analyses further confirmed the biological significance of selected phenolic compounds from acorns. Molecular docking revealed that tellimagrandin II, oenothein B, pentagalloylglucose, vescavaloninic acid, castalagin, and ellagic acid may contribute to the observed enzyme-inhibitory effects, with predicted affinities in several cases being comparable to or higher than those of reference ligands.

Overall, these findings support further investigation of *Q. rubra* and *Q. robur* acorn phenolic fractions as promising sources of bioactive compounds exhibiting antioxidant and enzyme-modulating properties under simulated gastric digestion conditions. However, further *in vivo* studies are needed to validate their biological activity, bioavailability, metabolism, and safety.

## 4 Materials and methods

### 4.1. Chemical reagents and materials

Gallic acid (GA), ellagic acid (EA), oenothein B, pentagalloylglucose, punicalagin, 6-hydroxy-2,5,7,8-tetramethylchroman-2-carboxylic acid (Trolox), 2,2-azino-bis-3-ethylbenzothiazoline-6-sulfonic acid (ABTS), 2,2-diphenyl-1-picrylhydrazyl (DPPH), 2,2,6,6-tetramethyl-1-piperidinyloxy (TEMPO), 2,4,6-tri(2-pyridyl)-*s*-triazine (TPTZ), sodium acetate, iron(iii) chloride hexahydrate, acetonitrile of HPLC grade (≥99.9%), formic acid for LC-MS (∼98%), pepsin from porcine gastric mucosa were all obtained from Sigma-Aldrich (St. Louis, MO, USA). Folin–Ciocalteu reagent, methanol, and ethanol were obtained from Chempur (Piekary Slaskie, Poland). Water was purified by a Milli-Q water purification system (Millipore Corp., Bedford, MA, USA). All reagents employed in the enzyme assays, including AChE from electrophorus electricus, acetylthiocholine iodide (AThI, >99%), 5,5′-dithiobis-(2-nitrobenzoic acid) (DTNB, >99%), and donepezil (>99%, used as positive control), type II porcine lipase, *p*-nitrophenyl acetate (NPA, >98%), orlistat (>98%, used as positive control), l-3,4-dihydroxyphenylalanine (l-DOPA, >98%), MBTH (>99%) and kojic acid (analytical standard, used as the positive control), rat intestinal acetone powder as the GLU source, *p*-nitrophenyl-α-d-glucopyranoside (NPG, >99%), acarbose (>95%, used as the positive control), porcine pancreatic AMY, PNM (>95%), phosphate-buffered saline (PBS), dimethyl sulfoxide (DMSO, 99%), and ethanol (EtOH, 99%) were obtained from Sigma-Aldrich (Prague, Czech Republic). TYR from mushroom was used and purchased from Medchem express (NJ, USA). All other chemicals were analytical grade. All assays were conducted in 96-well microplates obtained from Gama Group (České Budějovice, Czech Republic).

### 4.2. Plant materials

In October 2024, fully ripe acorns of *Q. rubra* L. were collected from a forest in southern Poland (GPS location: 50° 04′18.5″ N, 20°35′09.0″ E), and fully ripe acorns of *Q. robur* L. were collected from a forest in northern Poland (GPS location: 53°35′53.074″ N, 21°20′26.673″ E). After harvesting, the acorns were cleaned and manually deshelled, then immediately frozen and freeze-dried at −50 °C under a vacuum of 0.7 mbar for 48–72 h using a DELTA 1-24LSC freeze dryer (Martin Christ, Osterode am Harz, Germany). The freeze-dried acorns were subsequently vacuum-packed in plastic bags and stored at −20 °C until further experiments.

### 4.3. Preparation of the phenolic-rich fractions

The phenolic-rich fractions from *Q. rubra* and *Q. robur* acorns were prepared according to the method described by ref. 6, with some modifications. Prior to extraction, freeze-dried acorns were ground into powder (<0.50 mm) using a laboratory mill. Each sample was extracted with 70% aqueous ethanol using sonication for 30 min at 40 °C. The plant material-to-solvent ratio was 1 : 20 (w/v). The ultrasonic frequency and power were set at 37 kHz and 150 W, respectively. The extraction conditions and solvent composition were selected based on preliminary experiments, which indicated that these parameters were optimal for the efficient extraction of phenolic compounds from acorns. Following extraction, the samples were centrifuged at 4800×*g* for 10 min at 20 °C, and the residue was re-extracted twice under identical conditions. Supernatants obtained from the three extraction steps were combined and filtered through filter paper. Ethanol was subsequently removed under a stream of nitrogen, and the remaining aqueous extract was frozen and freeze-dried using a DELTA 1-24LSC freeze dryer (Martin Christ, Osterode am Harz, Germany) at −50 °C under a vacuum of 0.7 mbar for 48–72 h. The dried phenolic-rich acorn fractions were then ground and stored in sealed containers at −20 °C until further analysis.

The extraction yield of the freeze-dried phenolic-rich fractions was calculated as the ratio of the mass of the obtained freeze-dried fraction to the mass of the initial acorn powder and expressed as a weight percentage, according to eqn (1).1
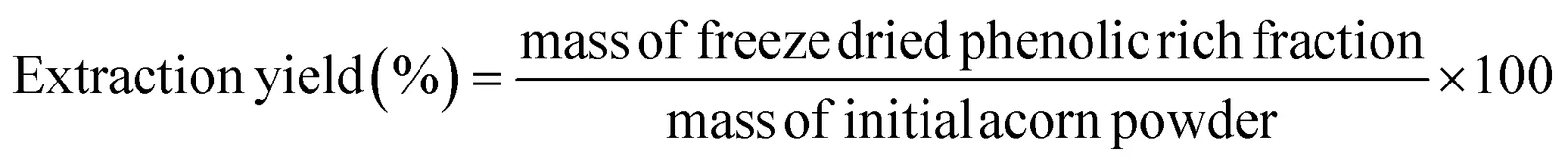


The enrichment factor was calculated based on the sum of individual phenolic compounds identified by UHPLC-DAD-ESI-HRMS/MS, as the ratio of the total content of identified phenolics in the freeze-dried fraction to their total content in the corresponding acorn powder, according to eqn (2).2
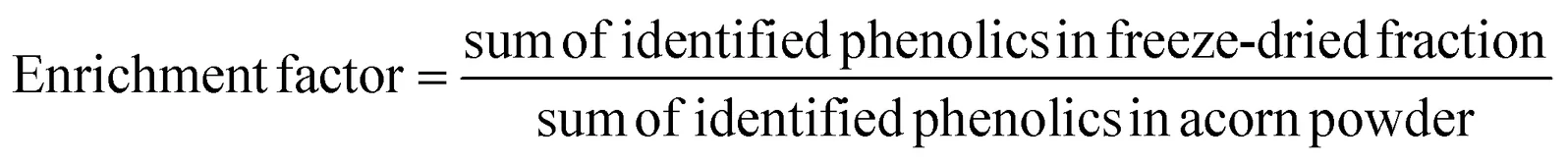


The recovery of identified phenolic compounds was calculated by taking into account both the extraction yield and the total content of identified phenolics in the obtained fraction, according to eqn (3).3
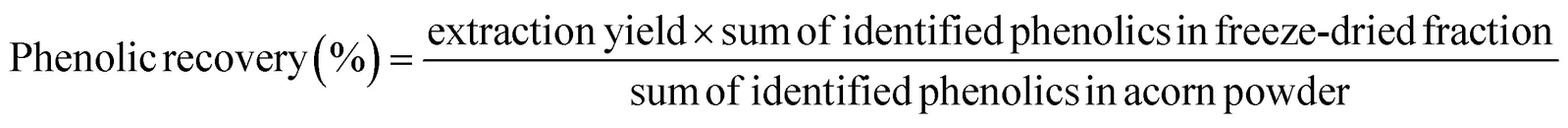


### 4.4. *In vitro* gastric digestion of the phenolic-rich fractions

Freeze-dried acorn phenolic fractions were subjected to simulated gastric digestion using a static *in vitro* model according to the procedure described by,^65^ with modifications. Only the gastric phase was applied to avoid potential interference from pancreatin and bile salts used during the intestinal phase, which could interact with phenolic compounds and complicate the interpretation of subsequent enzyme-inhibition assays. In brief, 15 mL of 0.1 M HCl (pH 2.0) was added to 1 g of dried phenolic-rich acorn fractions, followed by 15 mL of a pepsin solution prepared in 0.1 M HCl (55 mg of pepsin with an activity of 1200–1500 U mg^−1^). After thorough mixing, the samples were incubated for 2 h at 37 °C in a shaking water bath (SWB 22 N, LaboPlay, Bytom, Poland). After gastric digestion, the pH of the mixture was adjusted to 7.0 using 1 M NaOH. The samples were then centrifuged at 6000 rpm for 10 minutes at 20 °C, frozen and freeze-dried using a Delta 1-24 LSC freeze dryer (Martin Christ, Osterode am Harz, Germany) at −50 °C under a vacuum of 0.7 mbar for 48–72 h. The dried, digested fractions were ground and stored in sealed containers at −20 °C until further analysis.

### 4.5. Identification and quantitative analysis of phenolic compounds using UHPLC-DAD-ESI-HR-MS/MS

The phenolic compounds in the phenolic-rich acorn fractions and gastric-digested fractions were analysed using the method described by ref. 6, with slight modifications. Prior to analysis, 10 mg of each fraction was dissolved in 70% methanol, mixed and centrifuged at 6000 rpm for 10 minutes at 4 °C.

The resulting supernatant was then analysed using UHPLC-ESI-HR-MS/MS and UHPLC-DAD systems, both equipped with an Accucore™ C18 column (2.1 × 150 mm, 2.6 µm particle size; Thermo Scientific, PA, USA). The chromatographic separation conditions, including the injection volume, column temperature, flow rate, mobile phase composition, and gradient elution program, were applied as previously described by ref. 6.

Phenolic compounds were identified by UHPLC-ESI-HRMS/MS using a Transcend™ TLX-2 multiplexed LC system coupled to a Q Exactive hybrid quadrupole-Orbitrap mass spectrometer equipped with a heated electrospray ionization source (HESI-II) (Thermo Scientific, Hudson, NH, USA). The HR-MS/MS analysis parameters were as follows: negative ionization mode; full MS, parallel reaction monitoring (PRM), and data-dependent MS/MS acquisition modes; capillary temperature, 275 °C; heater gas temperature, 320 °C; electrospray capillary voltage, 4.5 kV; and S-lens radio-frequency (RF) level, 50 V. Nitrogen was used as both the sheath gas and auxiliary gas at flow rates of 35 and 15 arbitrary units, respectively. The full mass spectra were recorded in the 100–1500 *m*/*z* (mass/charge) at a mass resolution of 70 000 full width at half maximum (FWHM) at *m*/*z* 200, with an automatic gain control (AGC) target of 1.0 × 10^6^ and a maximum injection time (IT) of 100 ms. PRM and data-dependent MS/MS analyses were performed at a mass resolution of 35 000 FWHM at *m*/*z* 100, with an AGC target of 2.0 × 10^5^ and a maximum IT of 50 ms. The mass accuracy was below 4 ppm for all acquisition modes.

Quantitative analysis of phenolic compounds by UHPLC-DAD was performed using a Dionex UltiMate 3000 UHPLC system (Thermo Fisher Scientific Inc., Waltham, MA, USA) equipped with a multi-wavelength diode array detector. UV-vis absorption spectra were recorded over the wavelength range of 190–600 nm. Chromatograms were monitored at two wavelengths, 254 and 270 nm. Quantification was performed using the external standard method based on calibration curves prepared for available standards or structurally similar reference compounds.^6^ The results were expressed as mg g^−1^ of fraction dry weight.

### 4.6. Determination of total phenolic content (TPC) using the Folin–Ciocalteu method

Total phenolic content was determined spectrophotometrically using the Folin–Ciocalteu reagent, in accordance with the procedure described by ref. 65. The results were expressed in milligrams of gallic acid (GAE) per gram of fraction dry weight (mg GAE g^−1^).

### 4.7. Determination of *in vitro* antioxidant activity using spectrophotometric assays

The antioxidant activity of acorn extracts and gastric-digested fractions was determined using spectrophotometric DPPH, ABTS and FRAP assays according to the procedures previously described by ref. 65.

The results of all assays were calculated from calibration curves prepared with Trolox (TE) and expressed as µmol TE equivalents per gram of fraction dry weight (µmol TE g^−1^).

### 4.8. Determination of *in vitro* antioxidant activity using EPR spectroscopic assays

The Electron Paramagnetic Resonance (EPR) spectroscopic measurements were performed with the stabilized radicals of DPPH and TEMPO, according to the procedures previously described by ref. 66, with slight modifications. Aliquots (100 µL) of the extract solutions were mixed with an equal volume (100 µL) of a 1 mmol DPPH ethanolic solution or TEMPO solution prepared in buffer (pH 6.8).

A Trolox standard (1 mM) was used as a reference standard. For determination of antioxidant activity, the relative radical concentration was evaluated after 1 min of reaction. The EPR spectroscopic measurements of both stable radicals reduction were recorded using a Bruker EMXplus X-band spectrometer (Bruker BioSpin GmbH, Germany) with the following parameters: microwave attenuation 10 and 17 dB for DPPH and TEMPO, respectively; modulation amplitude 0.10 and 0.05 mT; center field 339.5 and 354.4 mT; scan range 10 mT for both radicals; and scan time 20 s for both measurements. The results of both assays were expressed as µmol Trolox equivalents per gram of fraction dry weight (µmol TE g^−1^).

### 4.9. Enzyme inhibitory effect using in-house assays

The acorn phenolic-rich fractions were tested against a panel of in-house developed enzyme inhibition assays according to the procedures previously described.^67,68^ In detail, the inhibitory assays targeted AChE (EC 3.1.1.7), PNLIP (EC 3.1.1.3), GLU (EC 3.2.1.20), AMY (EC 3.2.1.1), and TYR (EC 1.14.18.1) were applied to explore potential biological activities.

Specifically, AChE inhibition was examined in relation to cognitive function due to its key involvement in cholinergic signalling. The PNLIP assay was employed to assess anti-obesity potential *via* interference with lipid digestion, while GLU and AMY inhibition was used as an indicator of possible antidiabetic activity through modulation of carbohydrate metabolism.

TYR inhibition was investigated in relation to melanin synthesis and potential skin health promoting effects. For the TYR assay, the protocol described in ref. 68 using MBTH (10 mM) as a coupling agent was used. using an Epoch BioTek microplate reader (Vermont, USA). The AMY assay was based on a protocol from ref. 69 with slight modifications. Briefly, 50 µL of a sample were incubated with 50 µL of AMY (5 mg mL^−1^ in PBS) at 37 °C for 5 min.

The sample could be (a) an acorn fraction, (b) negative control (70% EtOH in PBS) or (c) an acarbose solution (50 mg L^−1^ in PBS) as a positive control. Following incubation of the sample with the enzyme, the substrate NPM was added (50 µL 1 mM) and the absorbance at 405 nm was measured after 15 min at 37 °C. For each sample, all measurements were performed in triplicate. A complete gastric digestion blank, containing simulated gastric fluid with pepsin but without acorn phenolic-rich fractions, was processed identically to the samples, including incubation, neutralization, centrifugation, freeze-drying, and reconstitution. The blank was tested in parallel in all enzyme inhibition assays to assess possible interference from residual pepsin-derived fragments and neutralization salts.

### 4.10. Molecular docking studies

The binding affinities of the selected acorn phenolic compounds were investigated using molecular docking with the template-based (if possible) or structure-based docking workflow implemented in the CB-Dock2 server.^70,71^ CB-Dock2 combines automatic binding-cavity detection using the CurPocket algorithm with molecular docking using AutoDock Vina version 1.1.2.^70,71^ The structure-based workflow identifies potential ligand-binding cavities on the protein surface and subsequently performs docking within the selected cavity. For each docking calculation, CB-Dock2 provides the predicted cavity volume, cavity center coordinates, and grid-box dimensions defining the docking search space; these parameters are reported directly in the corresponding docking-result figures.

Ligand structures were primarily obtained from the PubChem database (https://pubchem.ncbi.nlm.nih.gov/): ellagic acid (CID 5281855), gallic acid (CID 370), cretanin (PubChem N/A), oenothein B (CID 16129800), vascalagin (CID 168165), castalagin (CID 12302513), rugosin E (CID 16129738), tellimagrandin II (CID 151590), galloylglucose (CID 124375), vescavaloninic acid (SID 319498283), pentagalloylglucose (CID 65238), tyrosine (CID 6057), orlistat (CID 303401), and acetylcholine (CID 187). The structure of acarbose was extracted from the crystal structure of human pancreatic α-amylase (PDB ID: 3BAY). As cretanin was not available in the PubChem database, its structure was manually prepared using ACD/ChemSketch software (version 2021.1.0, Advanced Chemistry Development, Inc. (ACD/Labs), Toronto, ON, Canada, https://www.acdlabs.com/). Before docking, all ligands were subjected to geometry optimization using Avogadro2 (Hanwell, M. D.; Hutchison, G. R. Avogadro2 (Version 2.0). Zenodo, 2026. https://doi.org/10.5281/zenodo.19369545).

Spatial structures of the target enzymes were obtained from the Protein Data Bank (https://www.rcsb.org/): acetylcholinesterase (PDB ID: 4EY6), pancreatic lipase (PDB ID: 1ETH), pancreatic α-amylase (PDB ID: 3BAY), tyrosinase (PDB ID: 2Y9W), and α-glucosidase (PDB ID: 5NN8). The protein structures were submitted to the structure-based docking module of CB-Dock2, and the docking cavities were identified automatically by CurPocket. Docking was subsequently performed using AutoDock Vina 1.1.2 within the CB-Dock2 workflow. The resulting docking scores and predicted ligand poses were used for comparative analysis of ligand–protein interactions. Because the calculations were performed through the web-based CB-Dock2 workflow, parameters not exposed or independently adjustable through the server interface were not modified by the authors. Three-dimensional protein–ligand interaction visualizations were generated using the CB-Dock2 server, whereas two-dimensional interaction diagrams were prepared with LigPlot+ (v2.3.1).^72^

### 4.11. *In silico* prediction of ADME parameters and toxicity

*In silico* analyses were performed to predict the ADME parameters and toxicity profiles of the main phenolic compounds identified in acorn native and gastric-digested fractions. The chemical structures of the selected compounds were entered into the prediction platforms as canonical SMILES strings. The pkCSM online platform was used to evaluate the pharmacokinetic properties, including the absorption, distribution, metabolism and excretion parameters, according to the procedure described by ref. 61.

Toxicity profiles, including acute toxicity, predicted LD_50_ values, toxicity class, and selected toxicological endpoints such as hepatotoxicity, carcinogenicity, immunotoxicity, mutagenicity, and cytotoxicity, were predicted using the ProTox-3.0 platform.

All predictions were performed using the default settings on 23 September 2026. Since the outputs of online prediction tools may change over time due to updates of algorithms, databases, and model versions, the date of analysis was recorded to ensure reproducibility. The obtained results obtained were used to complement the data supporting the assessment of the potential bioactivity and safety of the analysed phenolic compounds.

### 4.12. Statistical analysis

The results are expressed as the mean ± standard deviation (SD) from three independent replicates. Statistical significance was assessed using a one-way analysis of variance (ANOVA), followed by a Tukey post hoc test. Differences were considered statistically significant at *p* < 0.05. All statistical analyses were performed using Statistica 13.0 software (StatSoft, Inc., Palo Alto, CA).

## Author contributions

Conceptualization: J. O.; data curation: A. P., M. S., G. K., A. S. T., J. O.; formal analysis: A. P., M. S., G. K., A. S. T., J. O.; funding acquisition: A. S. T, J. O.; investigation: A. P., B. S., M. S., G. K., K. K.-W., A. S. T., J. O.; methodology: M. S., G. K., A. S. T., J. O.; project administration: J. O.; resources: M. S., G. K., A. S. T., J. O.; supervision: J. O.; validation: M. S., G. K., A. S. T., J. O.; visualization: M. S., G. K., A. S. T., J. O.; writing – original draft: A. P., M. S., G. K., K. K.-W., A. S. T., J. O.; writing – review and editing: A. P., M. S., G. K., K. K.-W., A. S. T., J. O.

## Conflicts of interest

There are no conflicts to declare.

## Supplementary Material

RA-016-D6RA08016B-s001

## Data Availability

The datasets used and/or analysed during the current study are available from the corresponding author upon request. Supplementary information (SI): additional data on changes in phenolic composition following simulated gastric digestion, chemical structures of selected phenolic compounds, enzyme-inhibition heatmaps and dose–response curves, detailed molecular docking results, and *in silico* ADME and toxicity predictions. See DOI: https://doi.org/10.1039/d6ra08016b.
