# Supplementary material for: Gastric digestion-induced transformation of acorn phenolics from Quercus rubra and Quercus robur: antioxidant activity, multi-target enzyme inhibition, and molecular docking insights
Source: RSC Adv. 2026 Nov 2;16(49):67808–27. doi: 10.1039/d6ra08016b (PMC13725865; doi:10.1039/d6ra08016b)
Supplement: Supplementary file 1 [file RA-016-D6RA08016B-s001.pdf]

Supplementary Information (SI) for RSC Advances.
This journal is © The Royal Society of Chemistry

SUPPLEMENTARY MATERIAL

Gastric digestion-induced transformation of acorn phenolics from *Quercus rubra* and *Quercus robur*: antioxidant activity, multi-target enzyme inhibition, and molecular docking insights

Aleksandra Popowska[a], Barbora Strasserova[b], Marcin Szustak[c], Gabriela Kowalska[a], Kamila Kulbat-Warycha[a], Aristeidis S. Tsagkaris *[b] and Joanna Oracz*[a]

[a].Institute of Food Technology and Analysis, Faculty of Biotechnology and Food Sciences, Lodz University of Technology, 2/22 Stefanowskiego Street, 90-537 Lodz, Poland. E-mail: joanna.oracz@p.lodz.pl

[b].Department of Food Analysis and Nutrition, Faculty of Food and Biochemical Technology, University of Chemistry and Technology, Technická 5, 166 28, Prague, Czech Republic. E-mail: Aristeidis.Tsagkaris@vscht.cz

[c].Institute of Molecular and Industrial Biotechnology, Faculty of Biotechnology and Food Sciences, Lodz University of Technology, Lodz, 90-537, Poland.

*Corresponding author: Assoc. Prof. Joanna Oracz, Institute of Food Technology and Analysis, Faculty of Biotechnology and Food Sciences, Lodz University of Technology, 2/22 Stefanowskiego Street, Poland, 90-537 Lodz, Poland. Tel: +48 42 631 34 62, E-mail address: joanna.oracz@p.lodz.pl

*Corresponding author: Assoc. Prof. Aristeidis Tsagkaris, Department of Food Analysis and Nutrition, Faculty of Food and Biochemical Technology, University of Chemistry and Technology, Technická 5, 166 28, Prague, Czech Republic, Tel.: +420 220 444 387, E-mail address: Aristeidis.Tsagkaris@vscht.cz

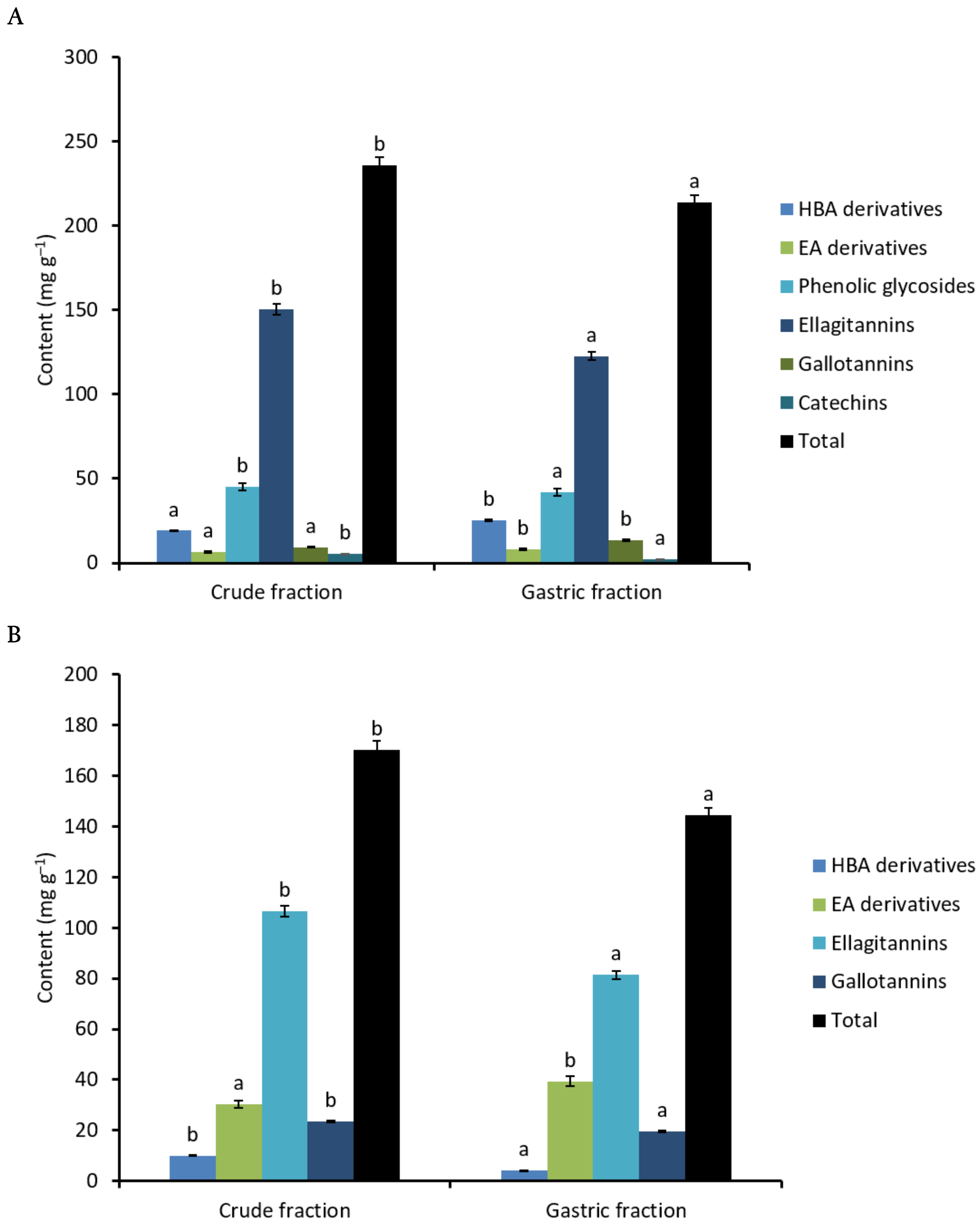

Fig S1. Changes in the concentration of individual groups of phenolic compounds in fractions from *Q. rubra* (A) and *Q. robur* (B) acorns after simulated gastric digestion. The data are expressed as mean values ± standard deviation from three independent replicates. Statistically significant differences between the crude extract and the gastric fraction are indicated by different letters above bars within the same phenolic compound group, according to Tukey's test at $p < 0.05$.

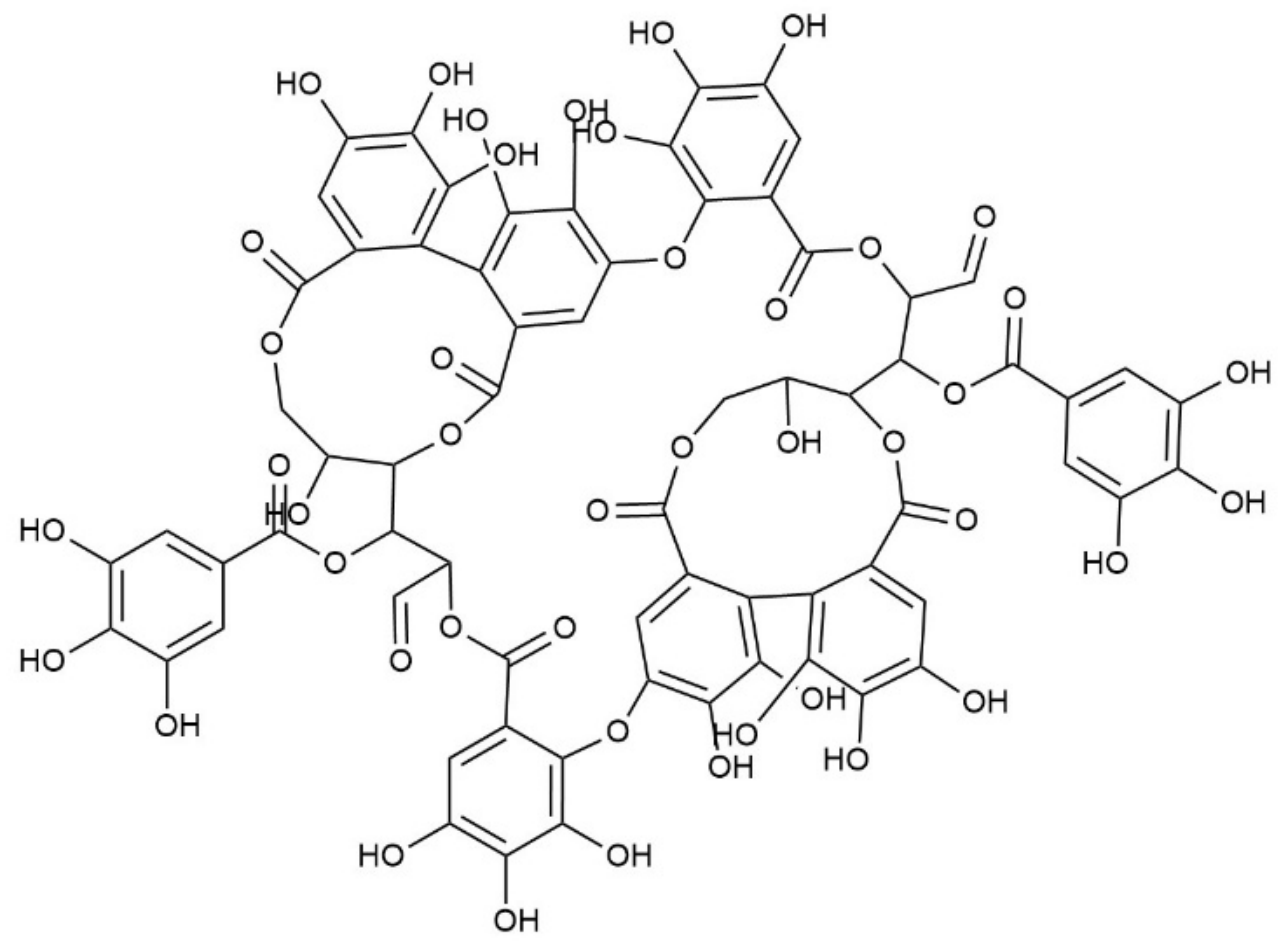

Oenothein B
$C_{68}H_{48}O_{44}$

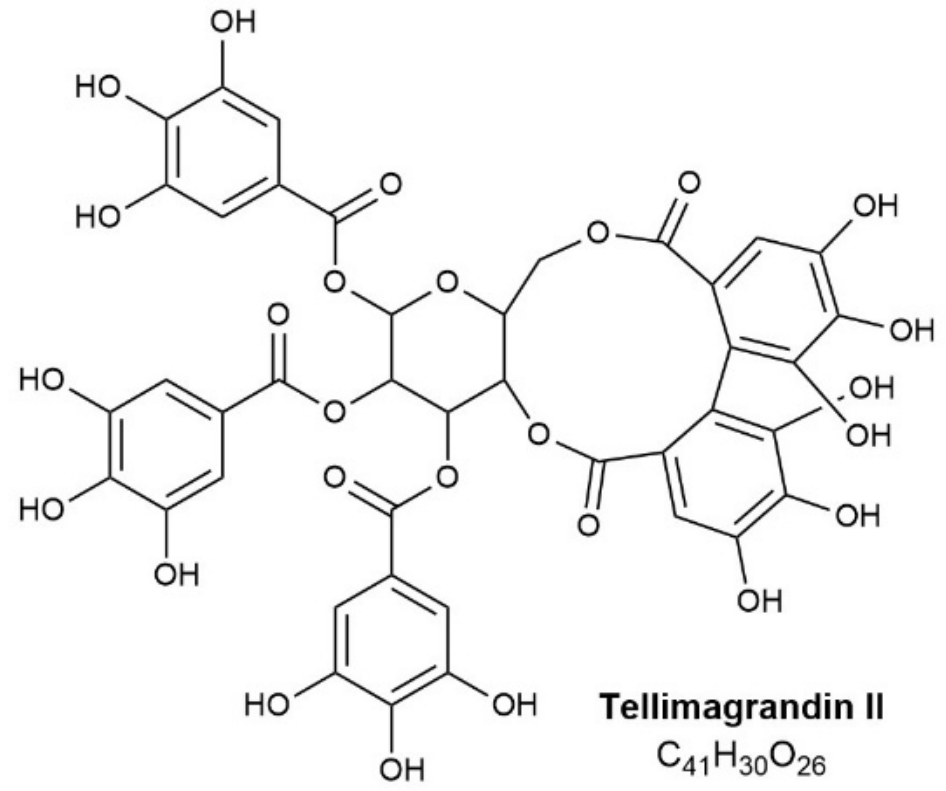

Tellimagrandin II
$C_{41}H_{30}O_{26}$

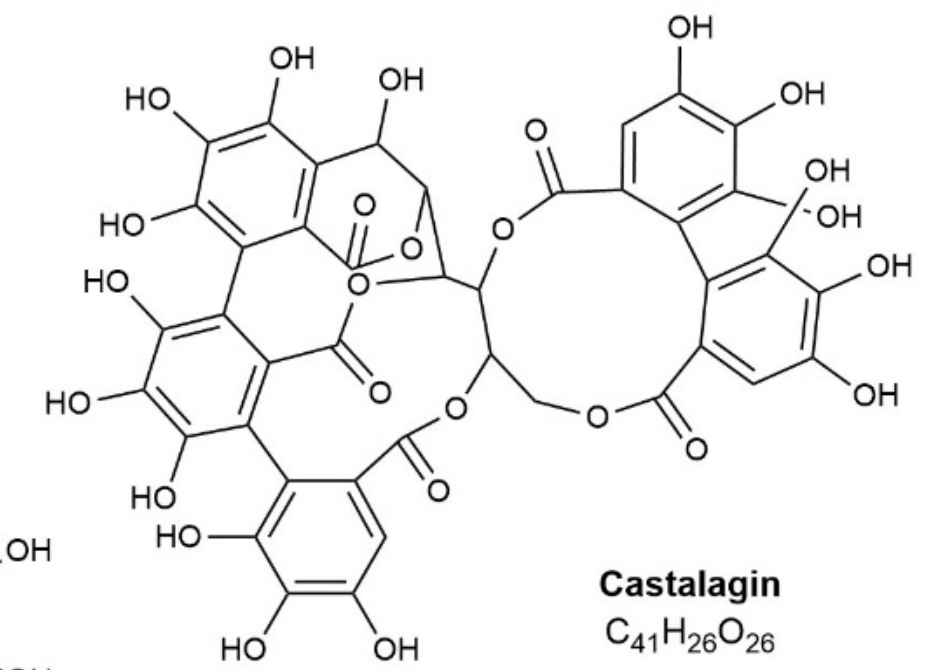

Castalagin
$C_{41}H_{26}O_{26}$

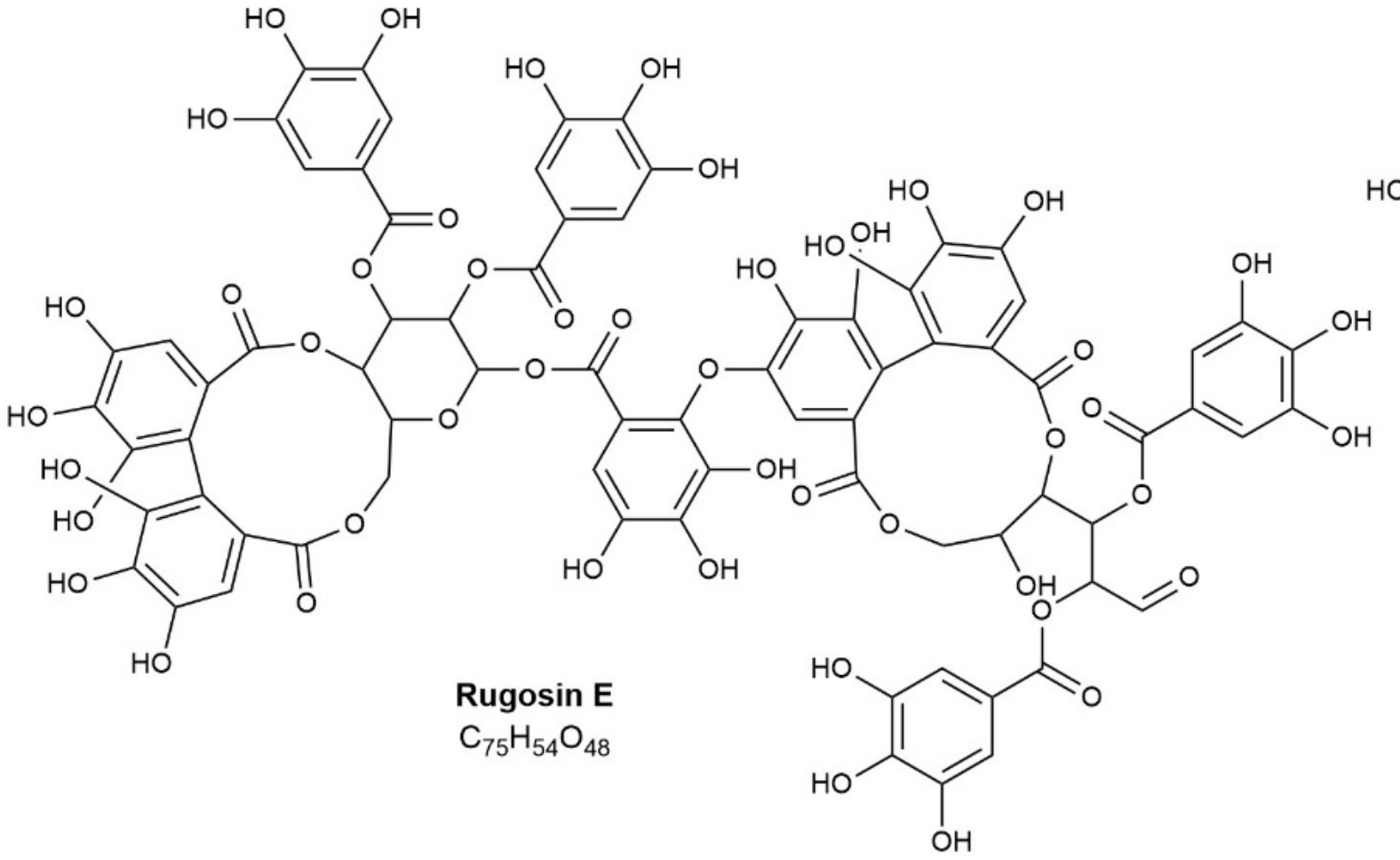

Rugosin E
$C_{75}H_{54}O_{48}$

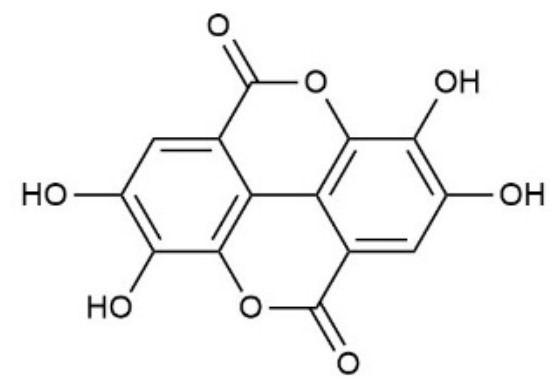

Ellagic acid
$C_{14}H_{6}O_{8}$

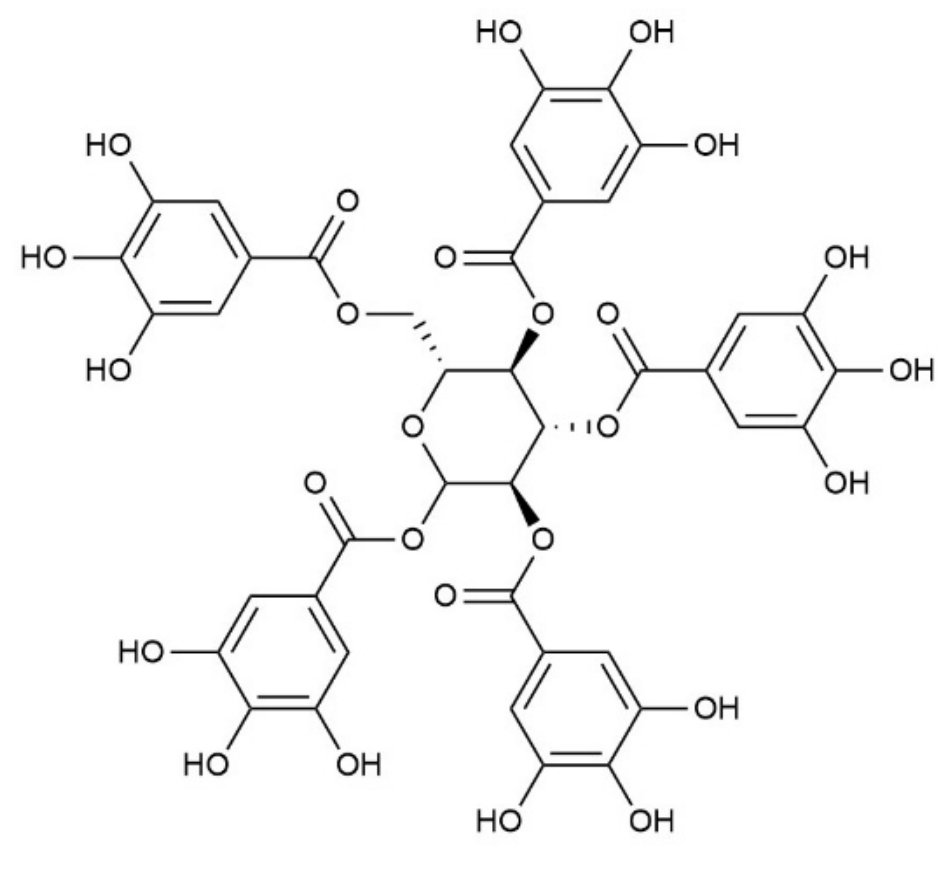

Pentagalloylglucose
$C_{41}H_{32}O_{26}$

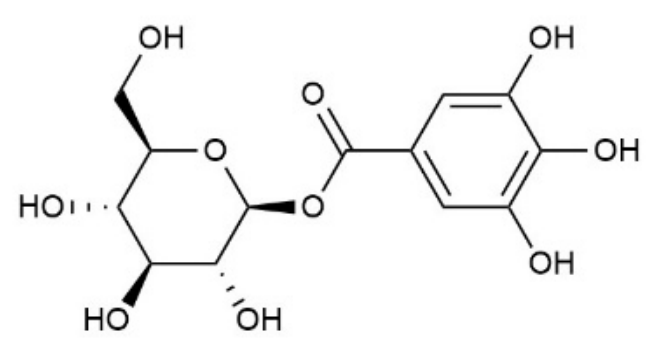

Galloylglucose
$C_{13}H_{16}O_{10}$

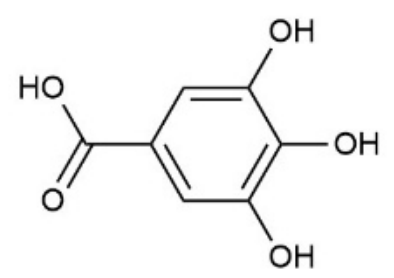

Gallic acid
$C_{7}H_{6}O_{5}$

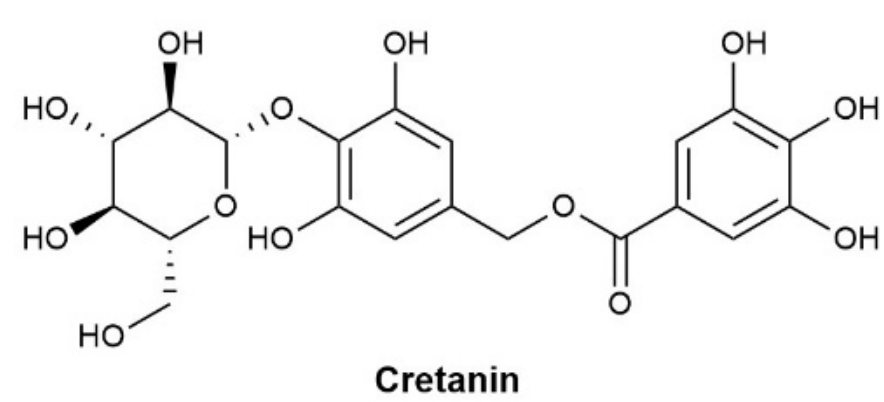

Cretanin
$C_{20}H_{22}O_{13}$

Fig S2. Chemical structures of selected phenolic compounds identified in *Q. rubra* and *Q. robur* acorns. Chemical structures were drawn using ChemDrawn software.

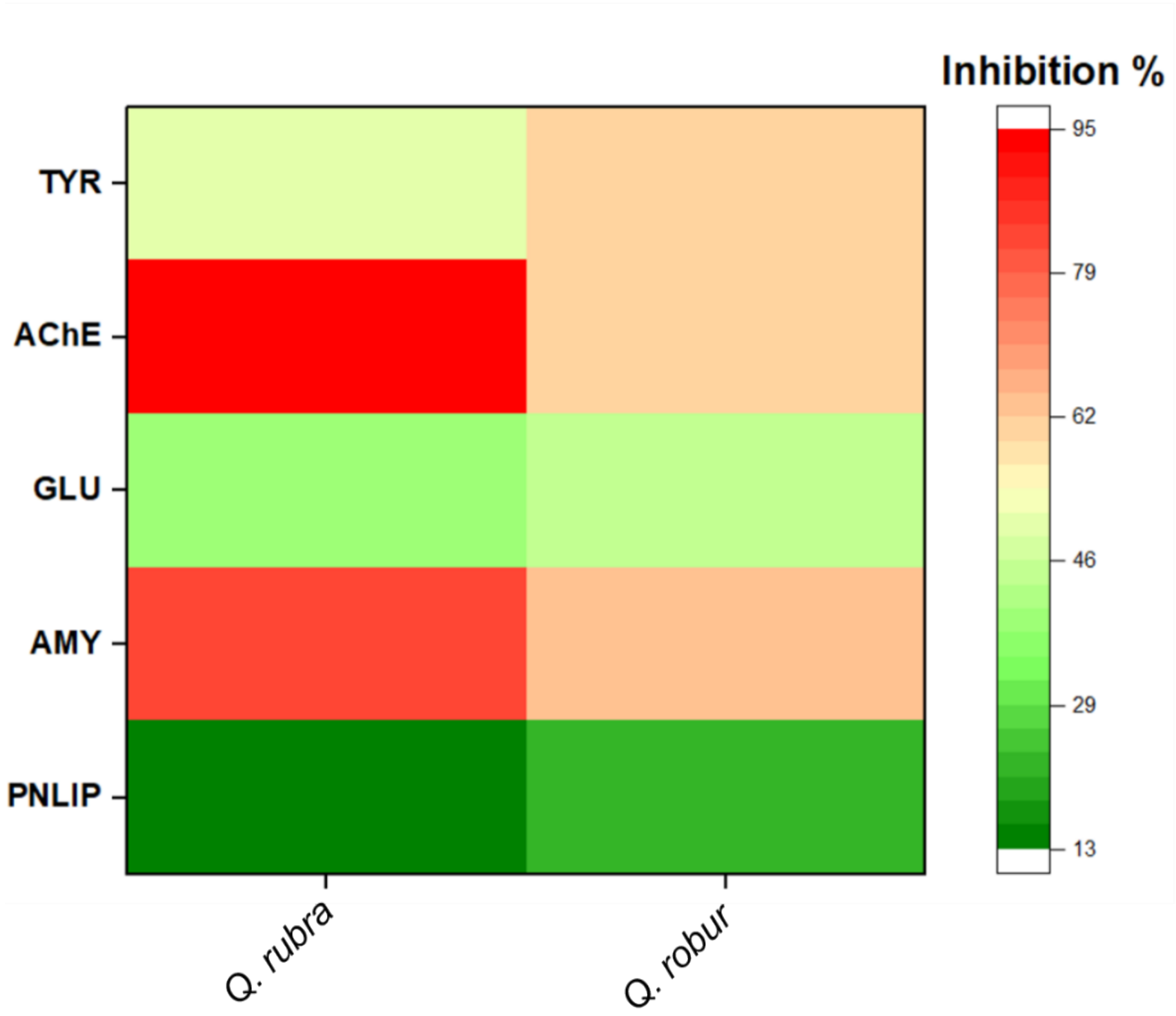

Fig. S3. Heatmap depicting the mean inhibitory effect (n= 3 in each case) for both oak species tested. The initial concentration of the tested fractions was 4 mg/mL.

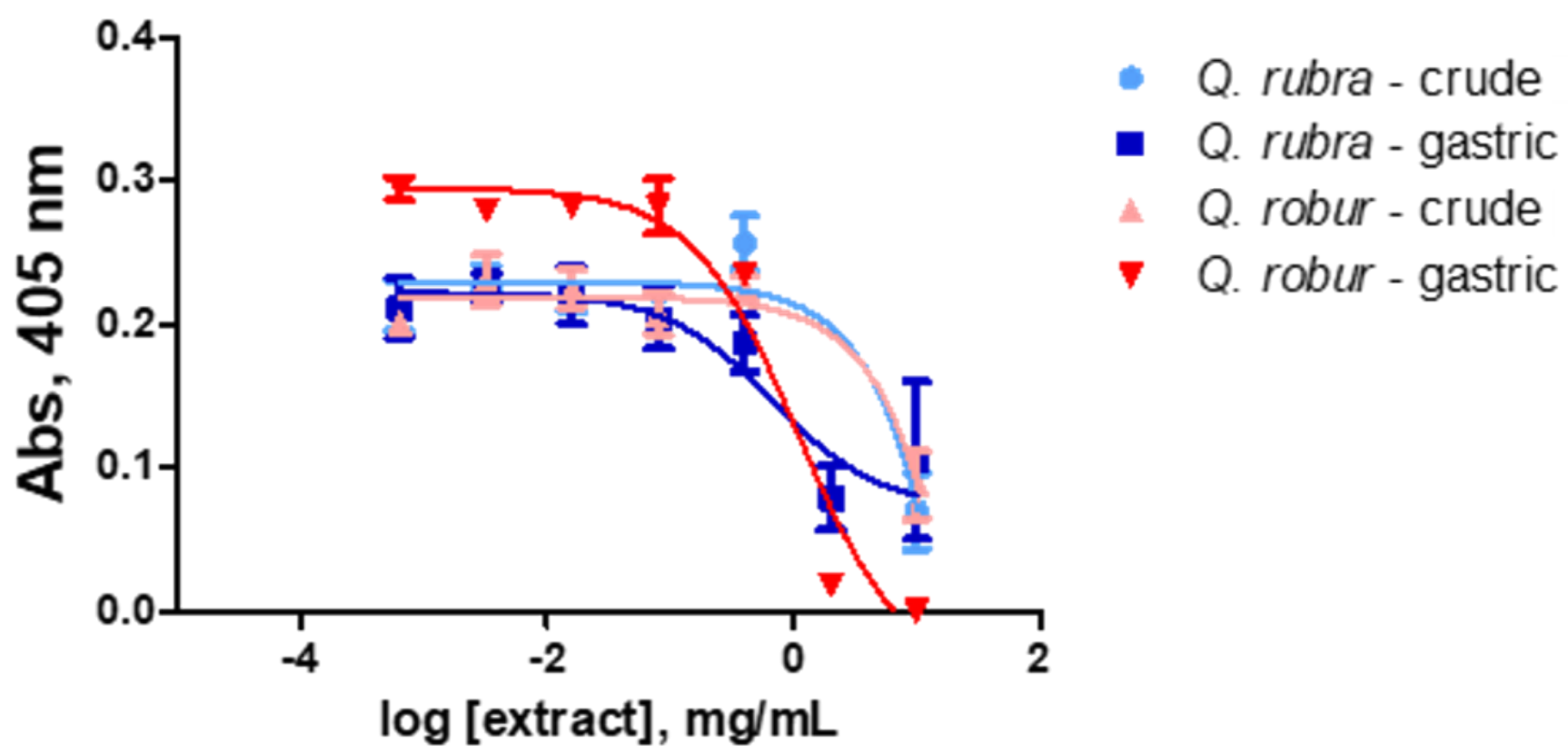

Fig. S4. Dose-response curves for the acorn phenolic fractions against PNLIP. Each concentration point was tested in triplicate (n= 3).

Table S1 Docking results of selected phenolic compounds with acetylcholinesterase, pancreatic lipase, α-amylase, and tyrosinase including predicted binding interactions and binding affinities.

Acetylcholineesterase (4EY6)				
No.	Compound	2D interactions	Contact residues	Affinity [kcal/mol]
1	Ellagic acid	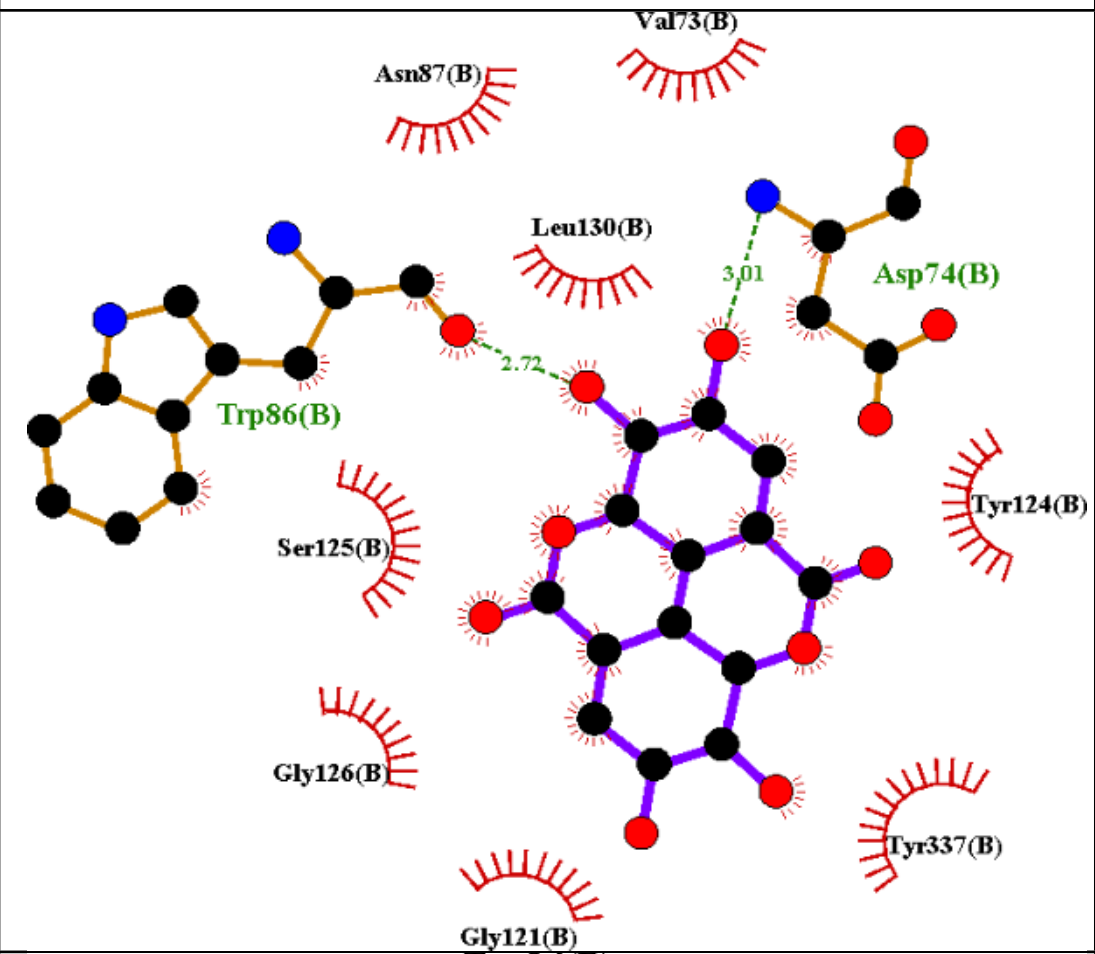	Q71 Y72 V73 D74 T75 L76 W86 N87 P88 Y119 G120 G121 G122 Y124 S125 G126 A127 L130 Y133 E202 S203 W286 L289 E292 S293 V294 F295 R296 F297 Y337 F338 Y341 H447 G448 I451	-10.0
2	Gallic acid	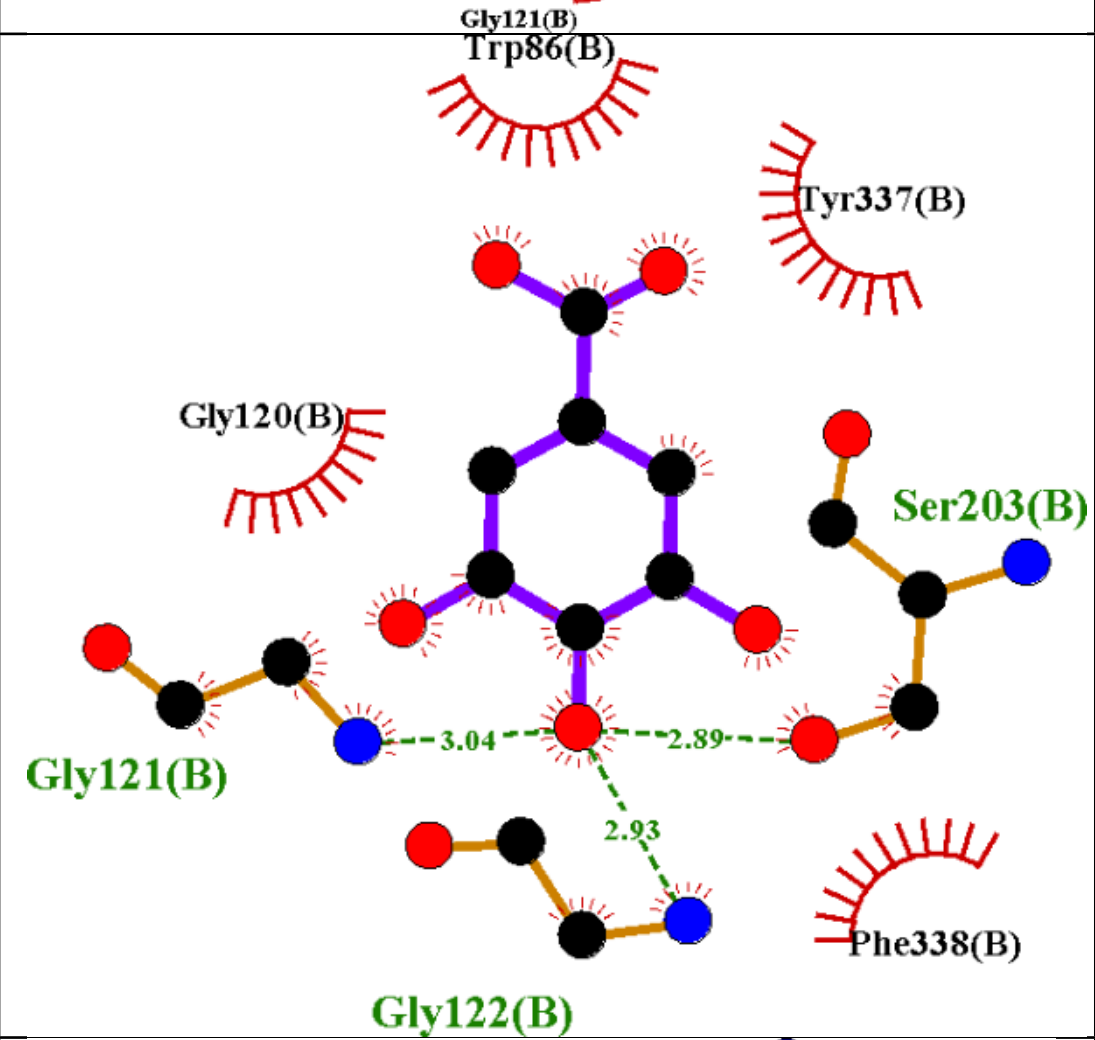	D74 G82 T83 W86 Y119 G120 G121 G122 F123 Y124 S125 G126 L130 Y133 E202 S203 A204 W286 L289 E292 S293 V294 F295 R296 F297 Y337 F338 Y341 G342 H447 G448 Y449 I451	-6.4
3	Cretanin	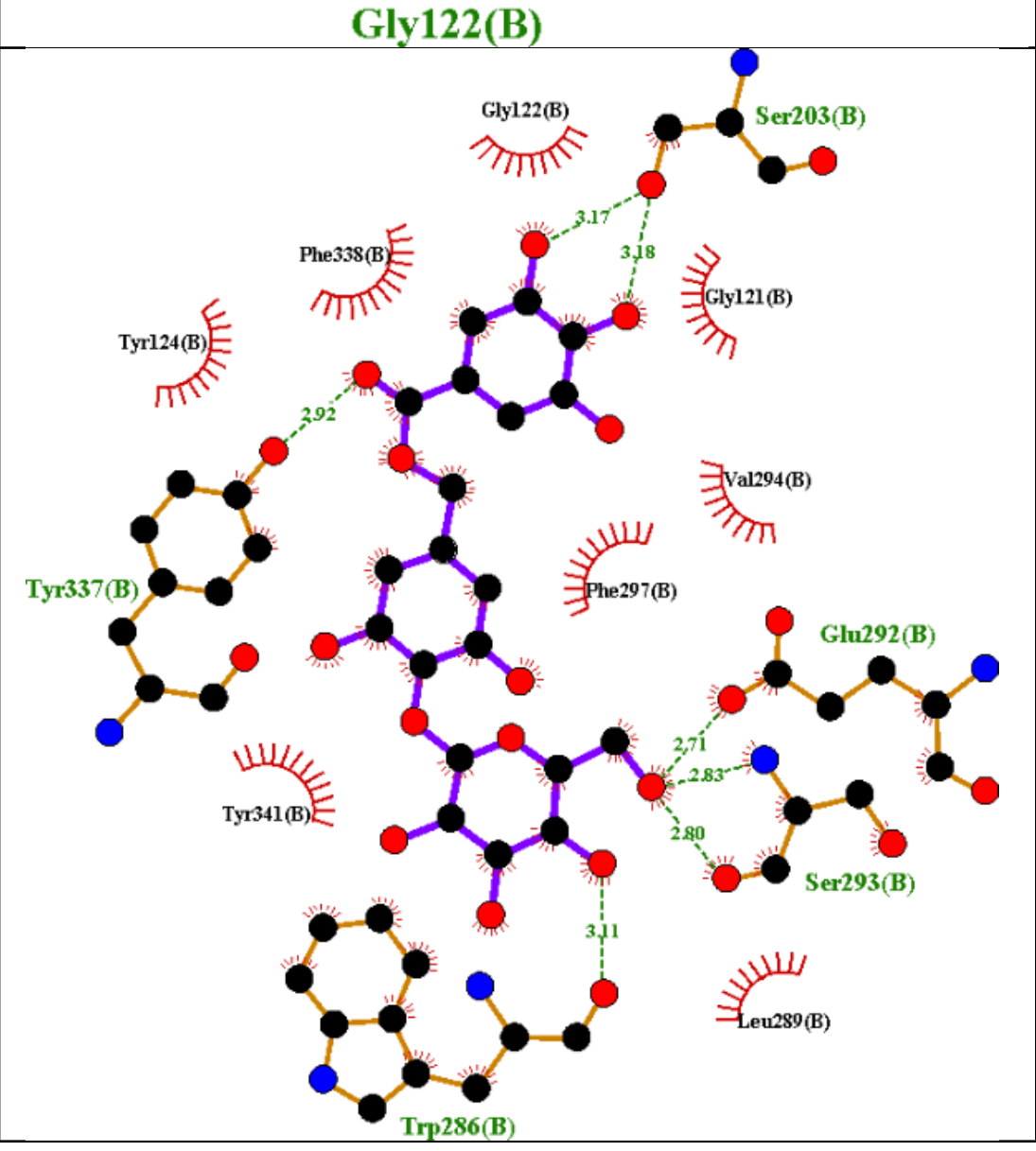	Q71 Y72 D74 T75 L76 T83 W86 N87 G120 G121 G122 F123 Y124 S125 G126 Y133 E202 S203 A204 W236 W286 H287 L289 Q291 E292 S293 V294 F295 R296 F297 Y337 F338 Y341 G342 H447 G448 Y449	-9.4

4	Oenothein B	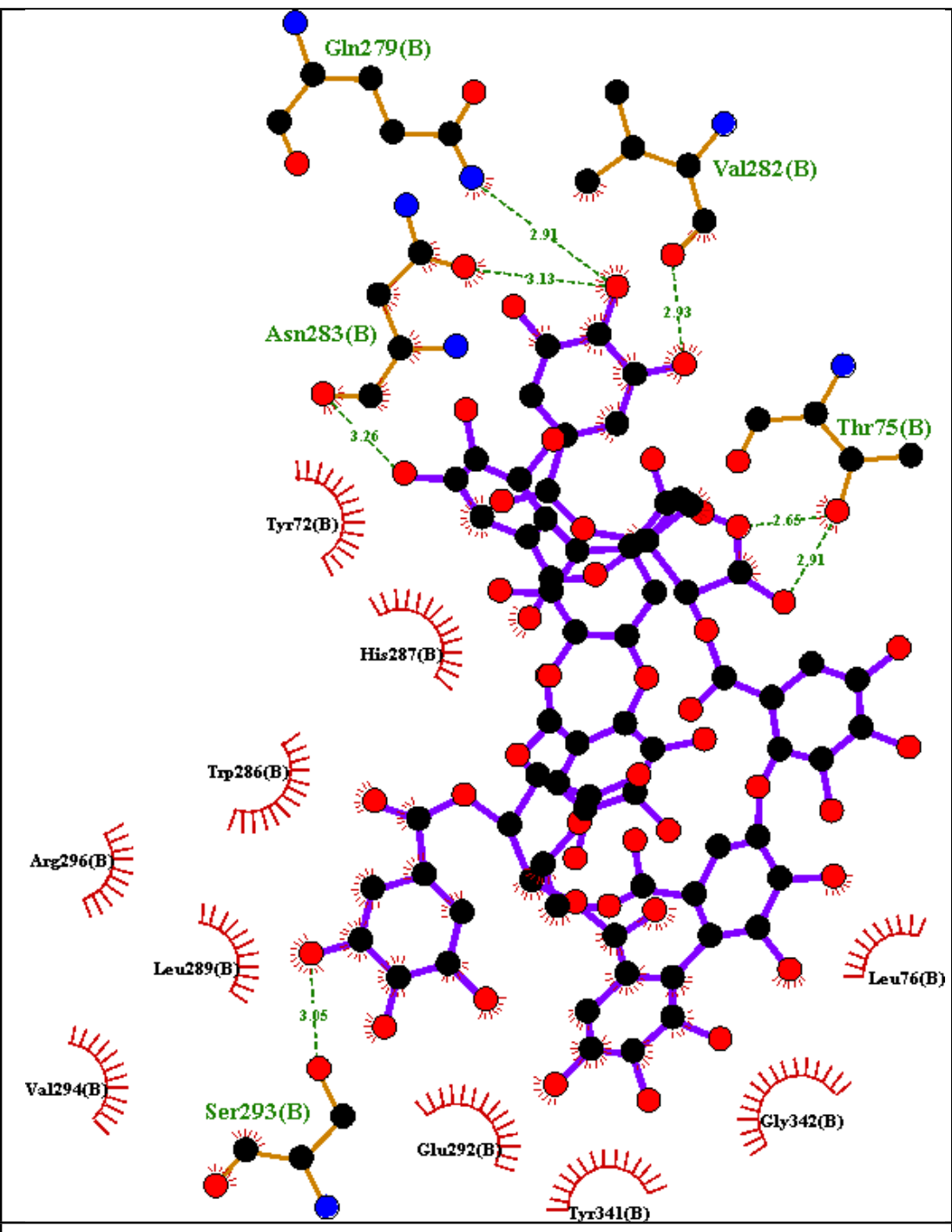	Y72 V73 D74<br>T75 L76 Y77<br>P78 Y124 Q279<br>V282 N283<br>H284 W286<br>H287 L289<br>Q291 E292<br>S293 V294<br>F295 R296<br>F297 Y337<br>F338 L339<br>V340 Y341<br>G342 A343<br>P344 G345<br>F346 S347<br>K348 D349<br>N350 V365	-7.2
5	Vescalagin	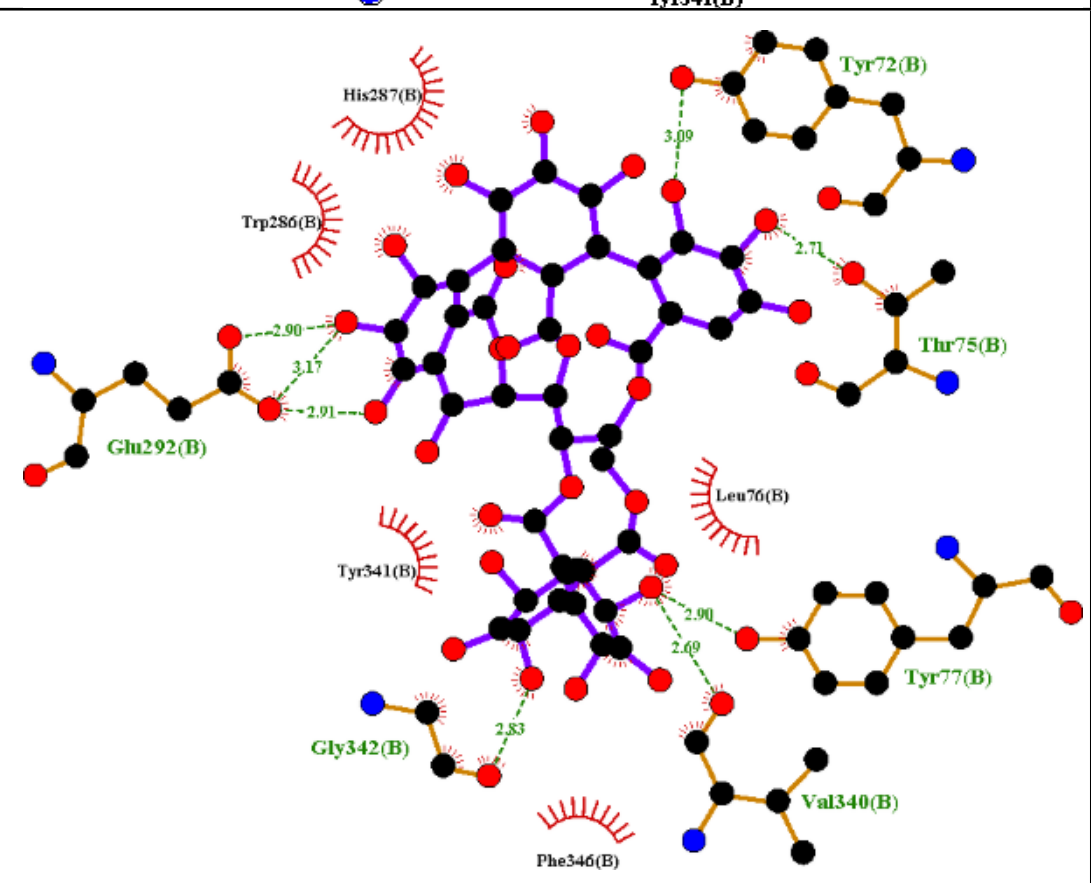	Q71 Y72 V73<br>D74 T75 L76<br>Y77 P78 Y124<br>Q279 V282<br>N283 H284<br>W286 H287<br>L289 Q291<br>E292 S293<br>V294 R296<br>L339 V340<br>Y341 G342<br>A343 P344<br>G345 F346<br>S347 K348<br>E358	-8.1
6	Castalagin	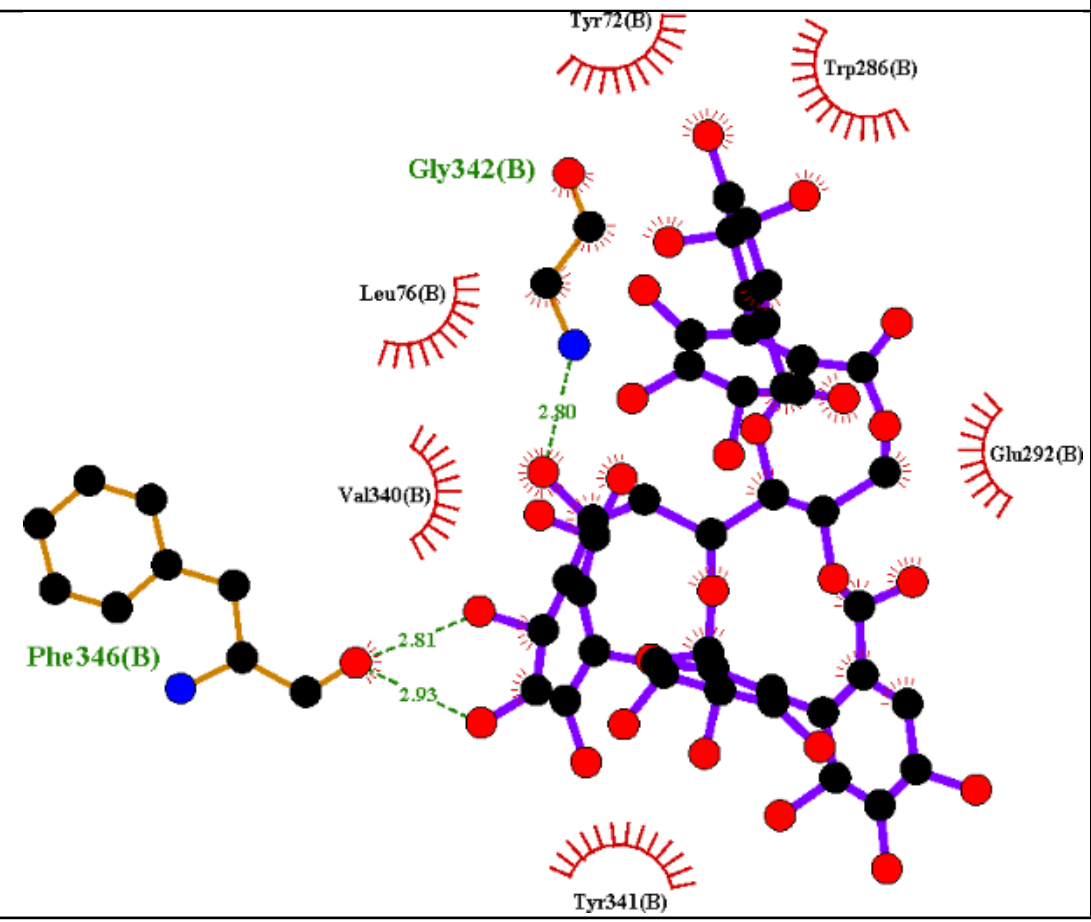	Y72 V73 D74<br>T75 L76 Y77<br>Y124 Q279<br>V282 N283<br>H284 E285<br>W286 H287<br>V288 L289<br>Q291 E292<br>S293 V294<br>R296 L339<br>V340 Y341<br>G342 A343<br>P344 F346<br>S347 V365	-7.3

7	Rugosin E	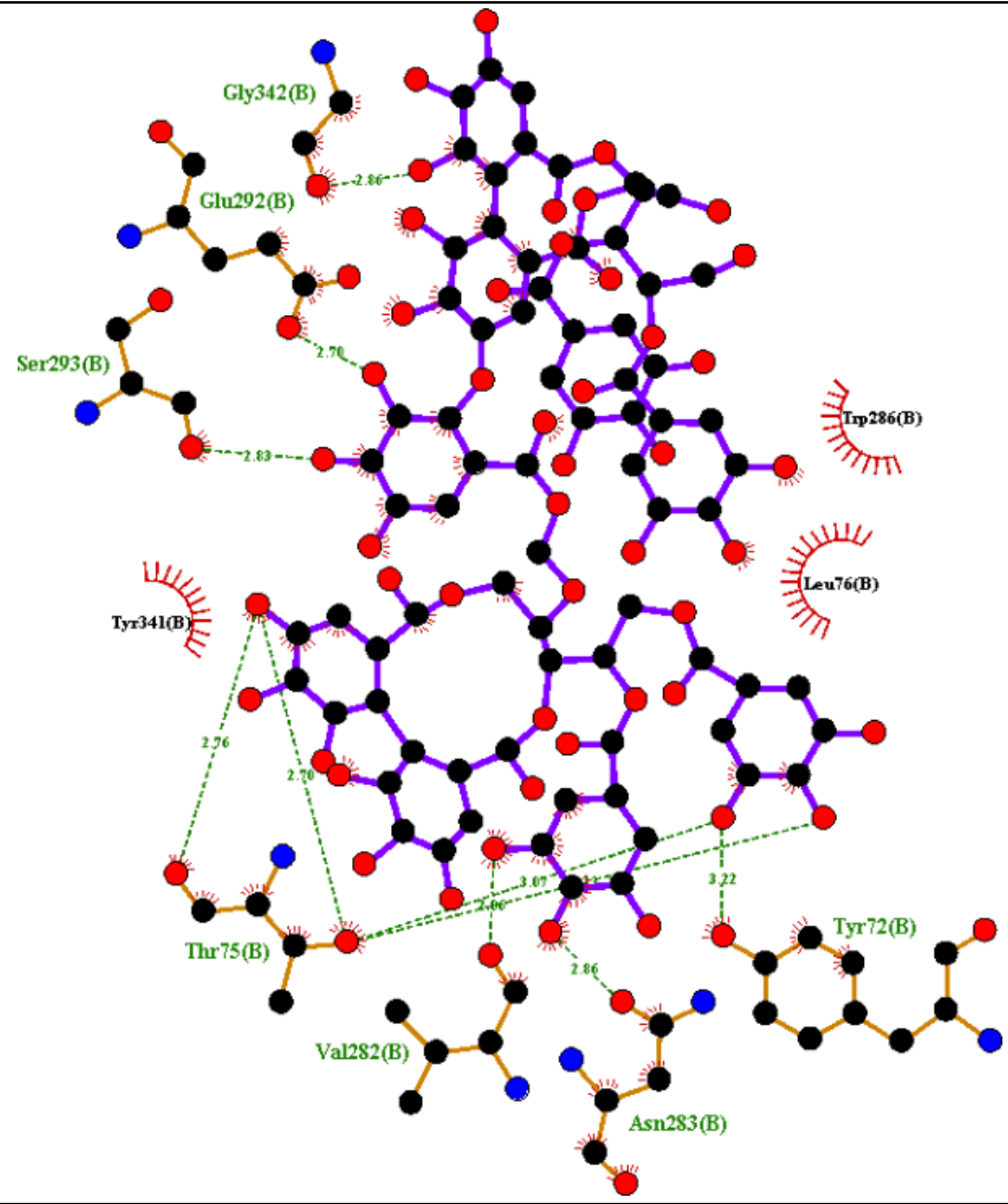	TYR72 VAL73 ASP74 THR75 LEU76 TYR77 PRO78 LEU92 TYR124 GLN279 VAL282 ASN283 HIS284 GLU285 TRP286 HIS287 LEU289 PRO290 GLN291 GLU292 SER293 VAL294 PHE295 ARG296 PHE297 PHE338 LEU339 VAL340 TYR341 GLY342 ALA343 PRO344 GLY345 PHE346 SER347 LYS348 ASN350 GLU358 LEU360 ALA361 GLY362 ARG364 VAL365	-7.4
8	Tellimagrandin II	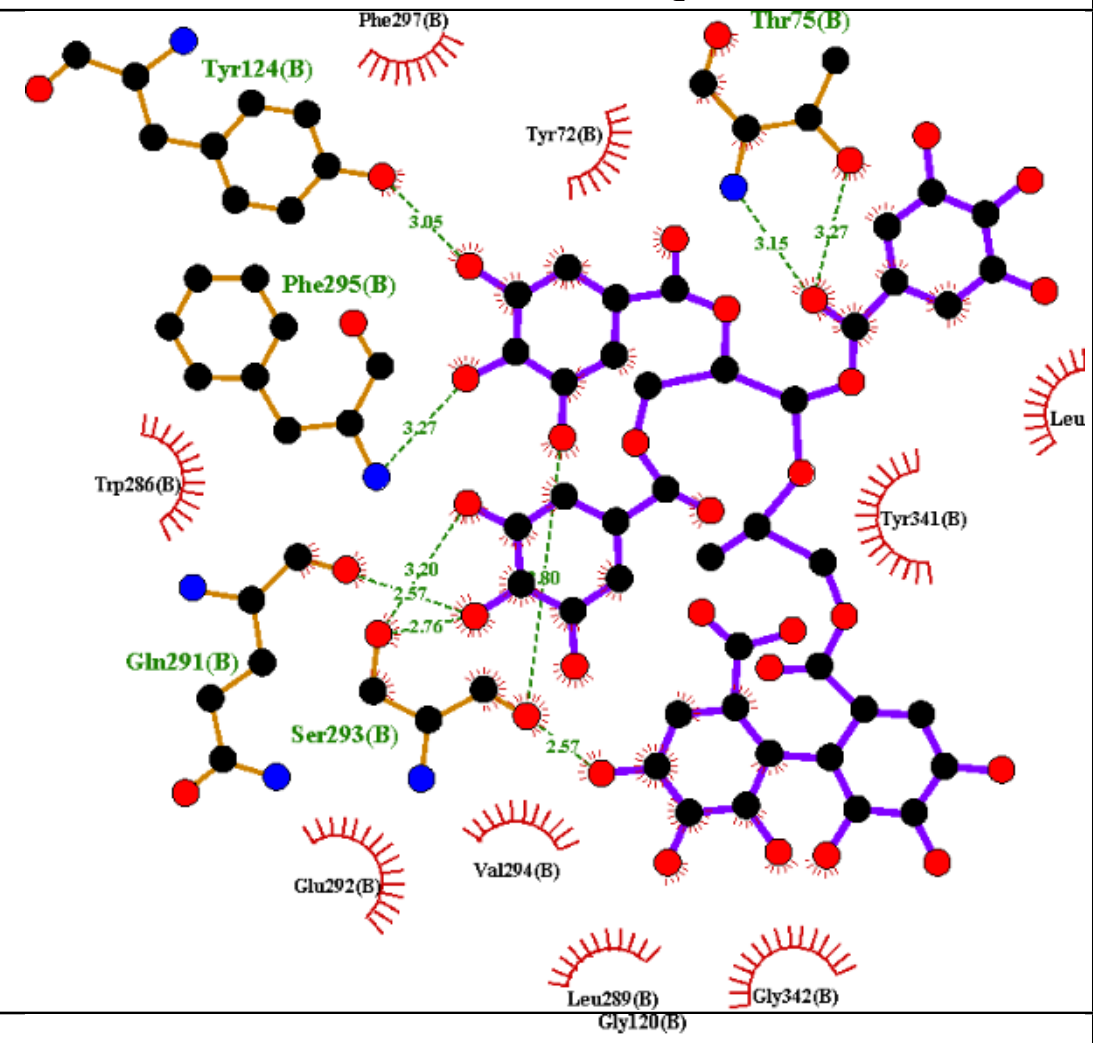	Y72 V73 D74 T75 L76 Y77 P78 Y124 V282 N283 H284 W286 H287 L289 P290 Q291 E292 S293 V294 F295 R296 F297 Y337 F338 L339 V340 Y341 G342 A343 P344 G345 F346 V365	-10.6
9	Galloylglucose	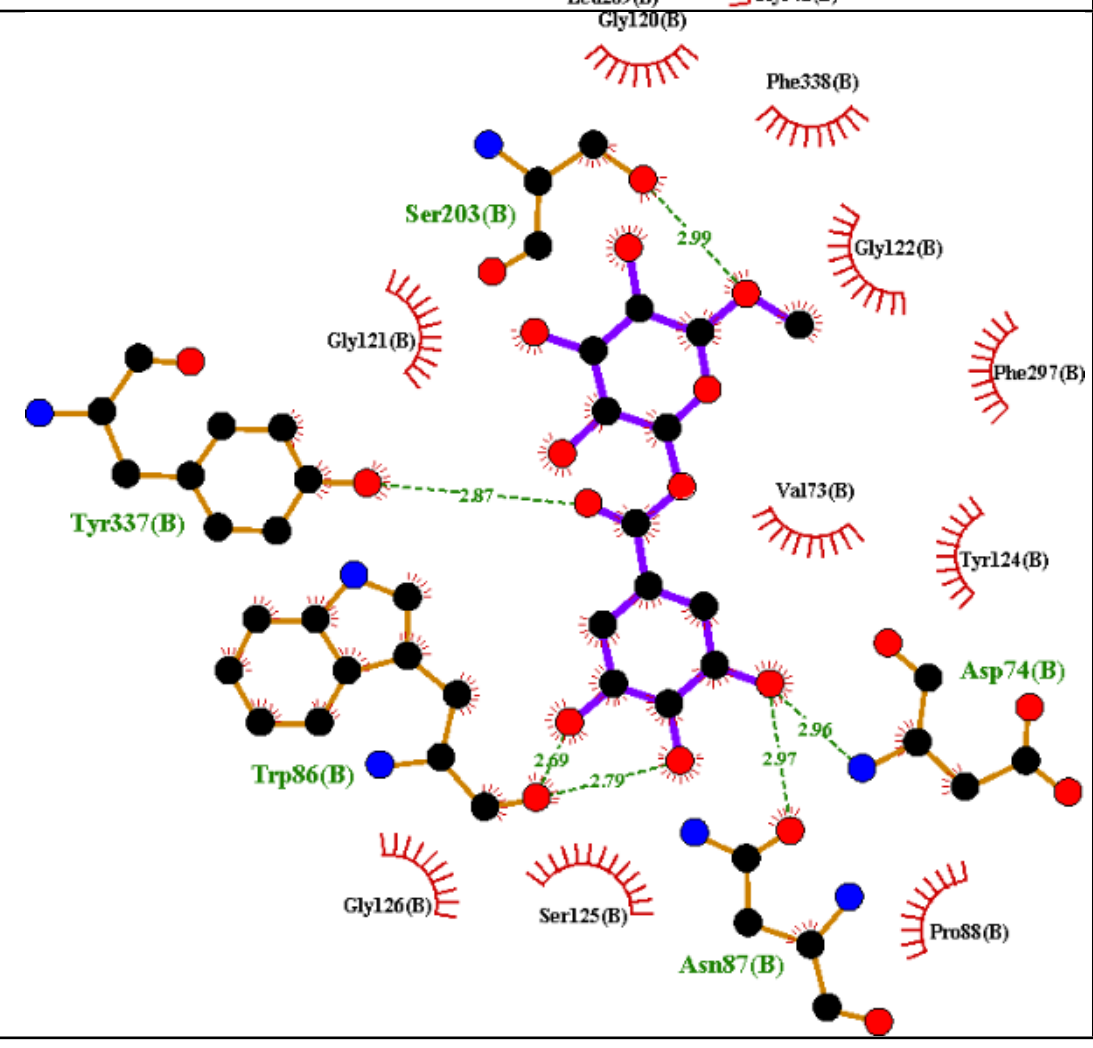	Q71 Y72 V73 D74 G82 T83 E84 M85 W86 N87 P88 Y119 G120 G121 G122 F123 Y124 S125 G126 A127 L130 Y133 E202 S203 A204 W286 F295 F297 Y337 F338 Y341 H447 G448 Y449 I451	-8.9

10	Vescavaloninic acid	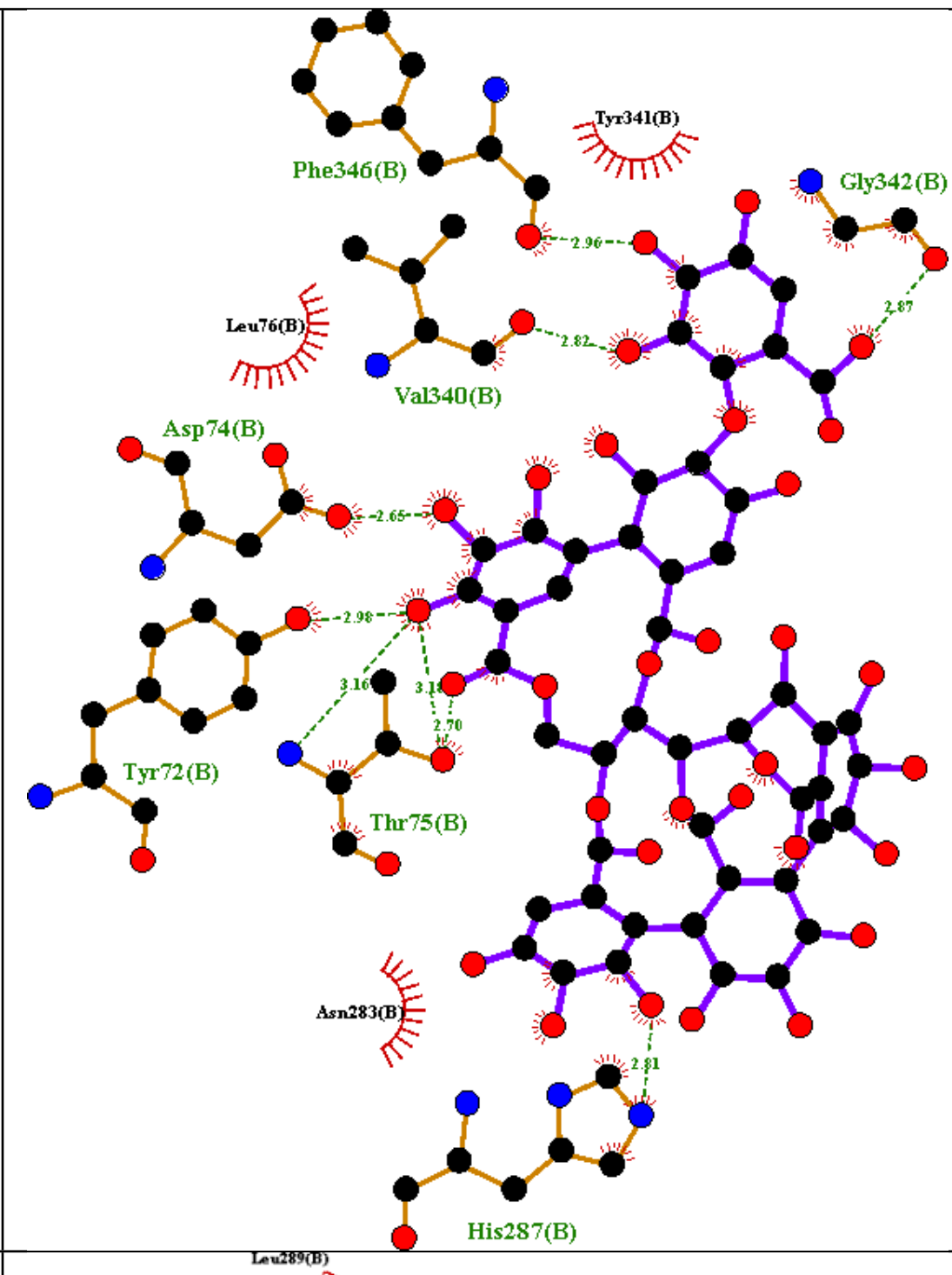	Y72 V73 D74<br>T75 L76 Y77<br>P78 Y124 Q279<br>V282 N283<br>H284 W286<br>H287 V288<br>L289 Q291<br>E292 S293<br>V294 F295<br>R296 F297<br>Y337 F338<br>L339 V340<br>Y341 G342<br>A343 P344<br>G345 F346<br>S347 V365	-8.5
11	Pentagalloylglucose	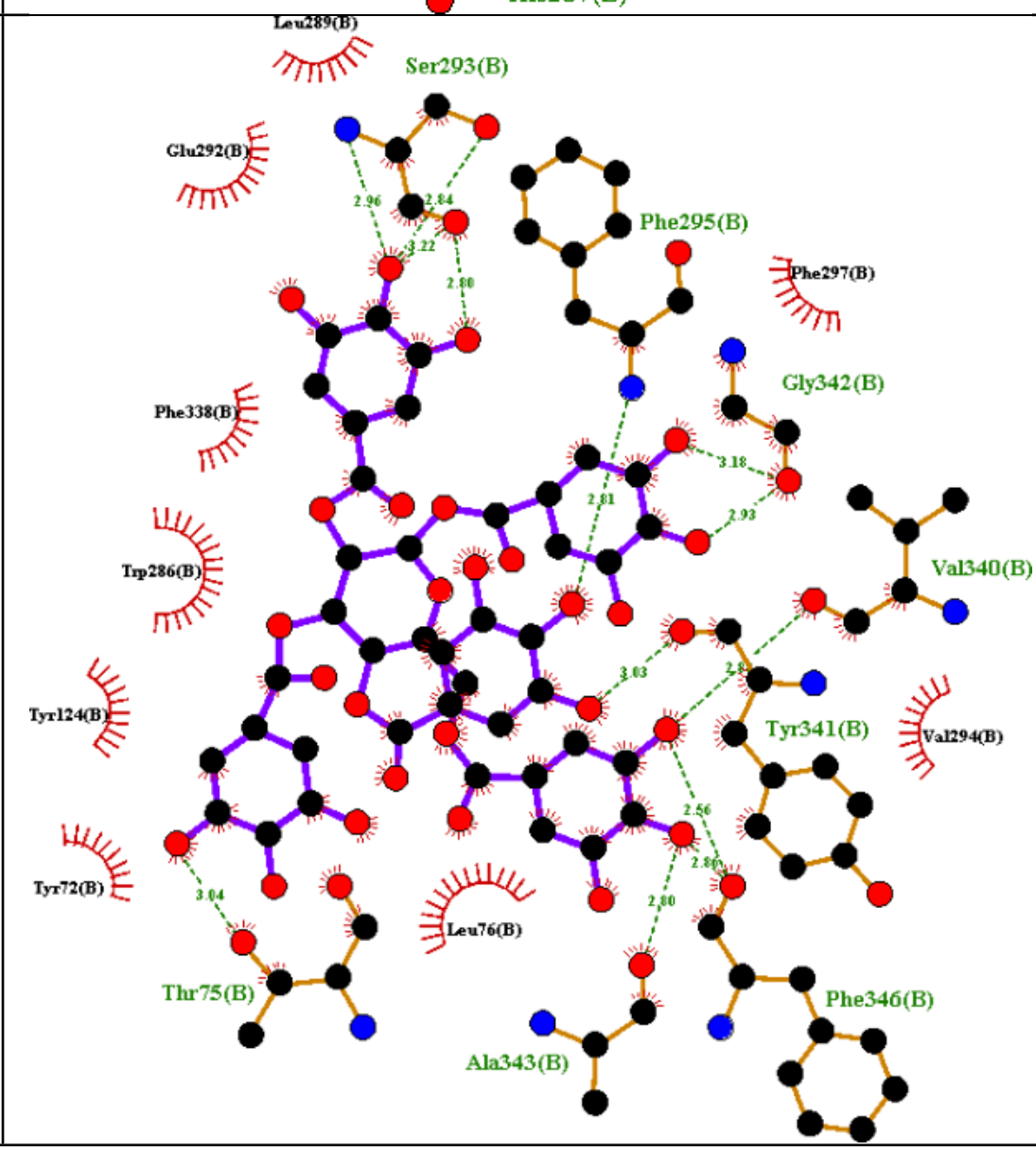	Y72 V73 D74<br>T75 L76 Y77<br>P78 Y124 V282<br>N283 H284<br>E285 W286<br>H287 L289<br>P290 Q291<br>E292 S293<br>V294 F295<br>R296 F297<br>Y337 F338<br>L339 V340<br>Y341 G342<br>A343 P344<br>G345 F346<br>S347 V365	-9.7

12	Acetylcholine (control)	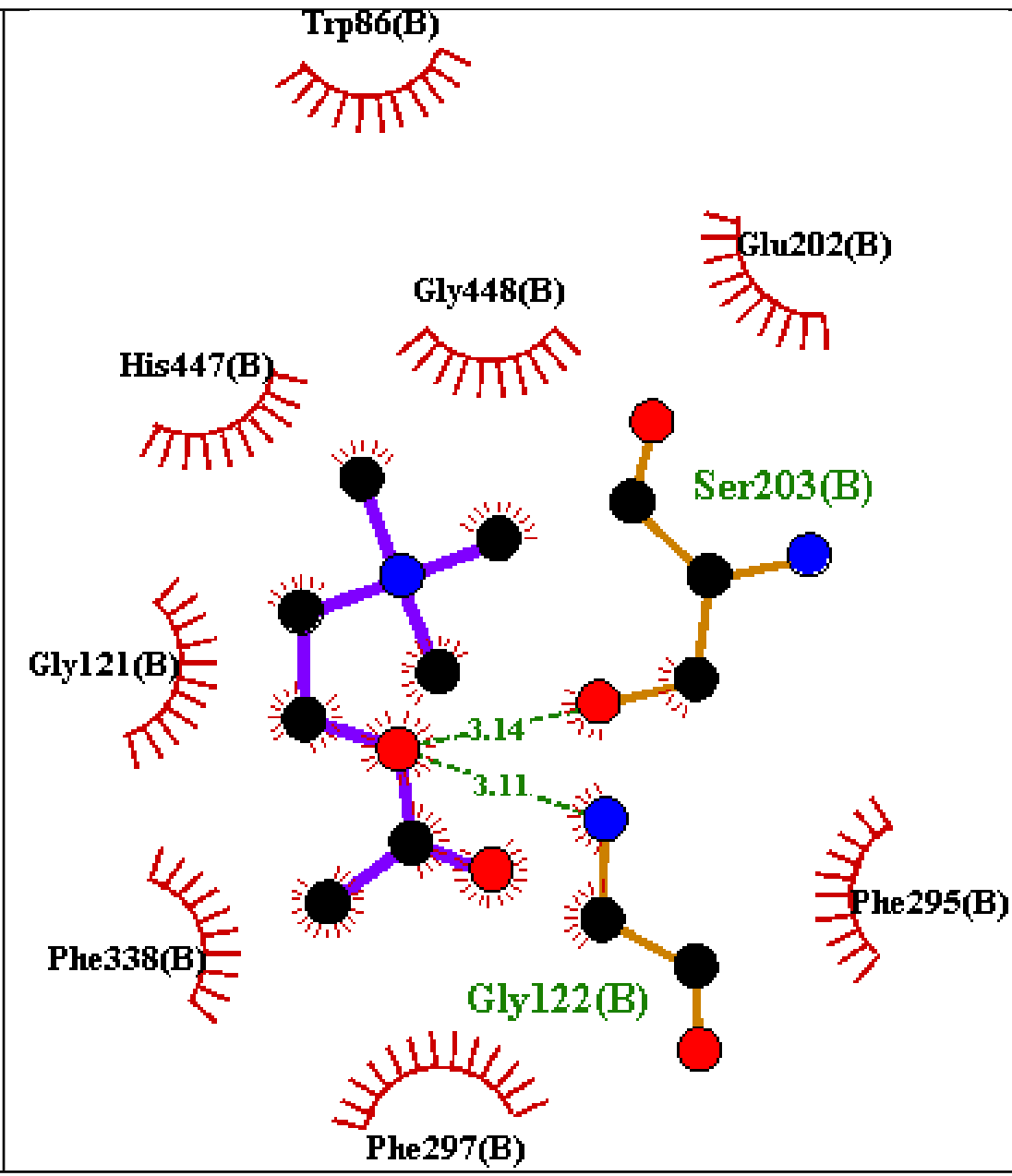	D74 T83 W86 N87 G120 G121 G122 Y124 S125 Y133 E202 S203 A204 W286 S293 V294 F295 R296 F297 Y337 F338 Y341 H447 G448 Y449 I451	-7.6
Pancreatic lipase (1ETH)				
No.	Compound	2D interactions	Contact residues	Affinity [kcal/mo]
1	Ellagic acid	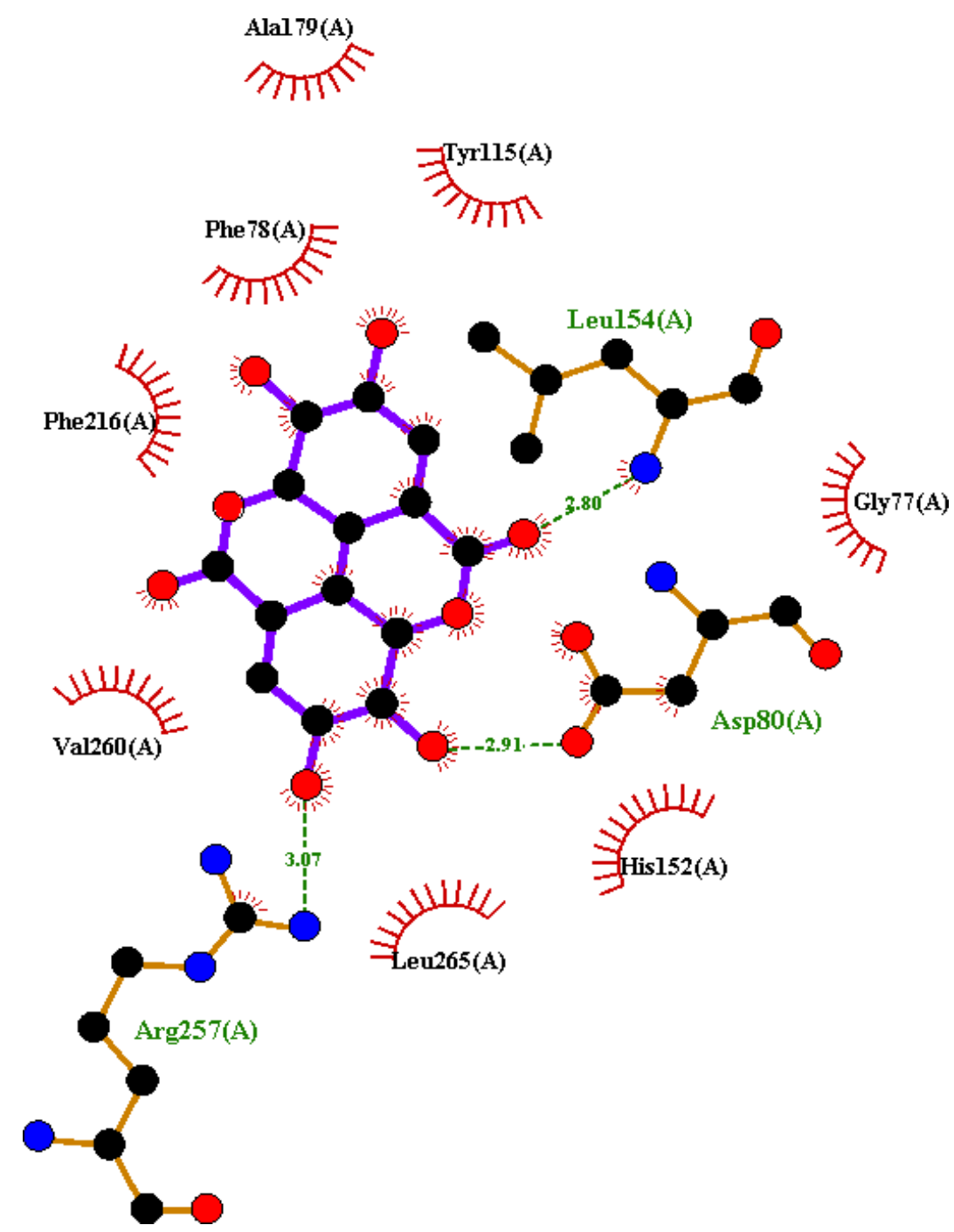	H76 G77 F78 I79 D80 W86 Y115 H152 L154 G155 D177 P178 A179 E180 P181 D206 A207 A208 P209 N213 L214 G215 F216 K233 Q234 C238 Q239 Q245 R257 D258 F259 V260 A261 C262 N263 L265 Y268	-9.2

2	Gallic acid	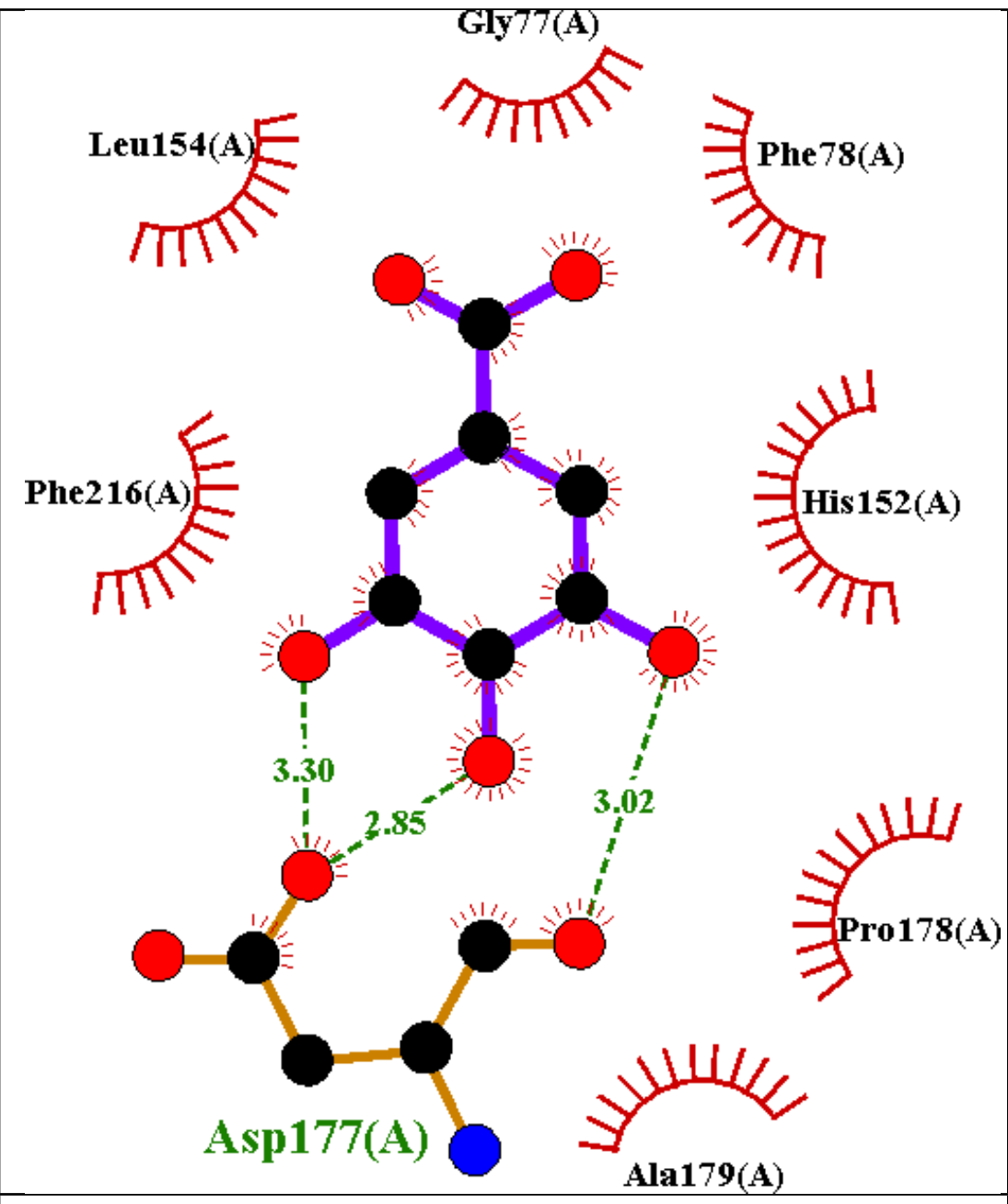	G77 F78 I79 Y115 H152 L154 G155 S156 L176 D177 P178 A179 E180 D206 N213 L214 G215 F216 G217 C238 Q239 Q245 R257 D258 F259 V260 A261 C262 N263 L265 S267	-6.3
3	Cretanin	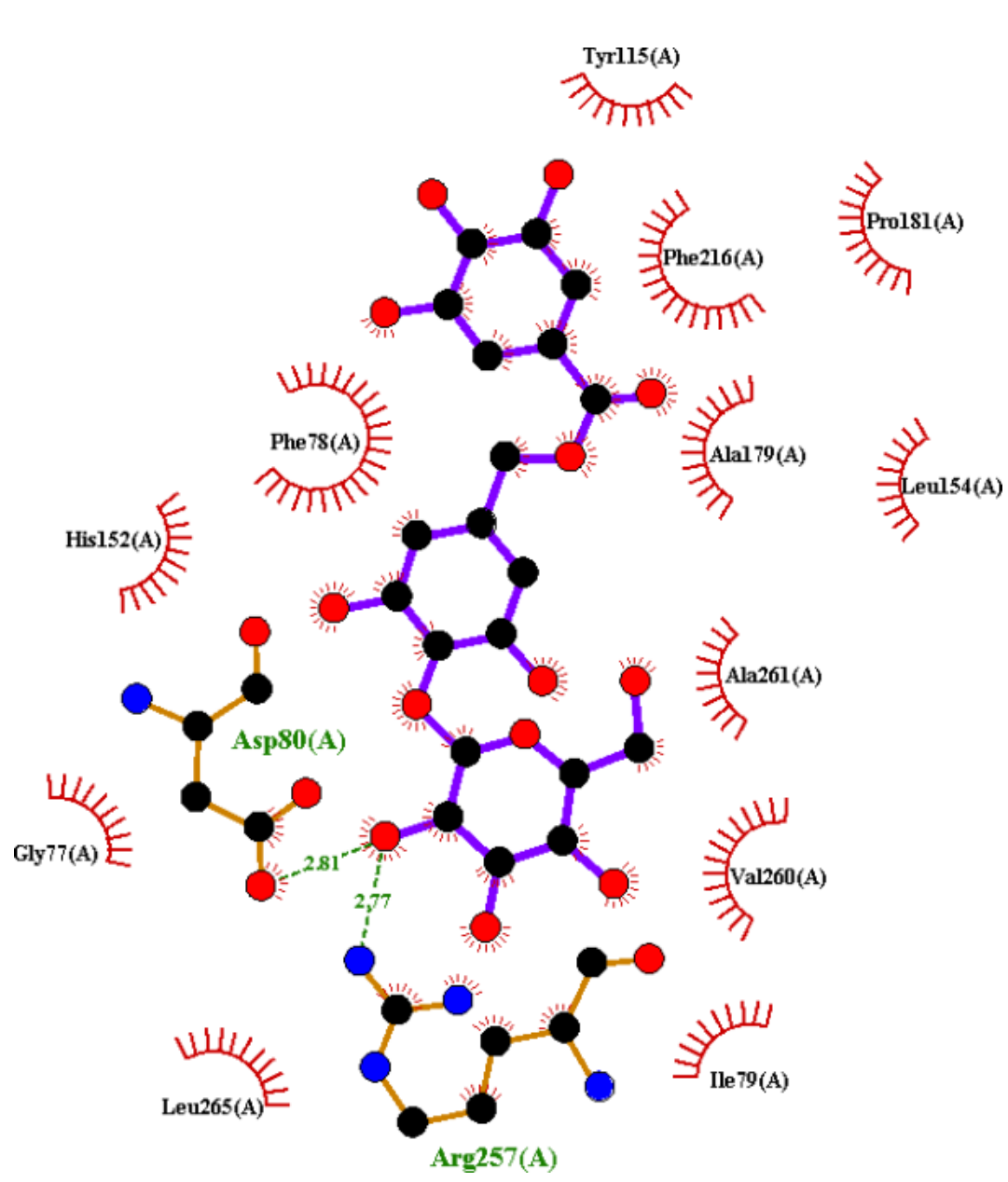	H76 G77 F78 I79 D80 W86 Y115 T116 H152 L154 G155 S156 L176 D177 P178 A179 E180 P181 D206 A207 A208 I210 N213 L214 G215 F216 G217 K233 Q234 G237 C238 Q239 K240 N241 I242 Q245 W253 R257 D258 F259 V260 A261 C262 N263 L265 S267 Y268	-8.4

4	Oenothein B	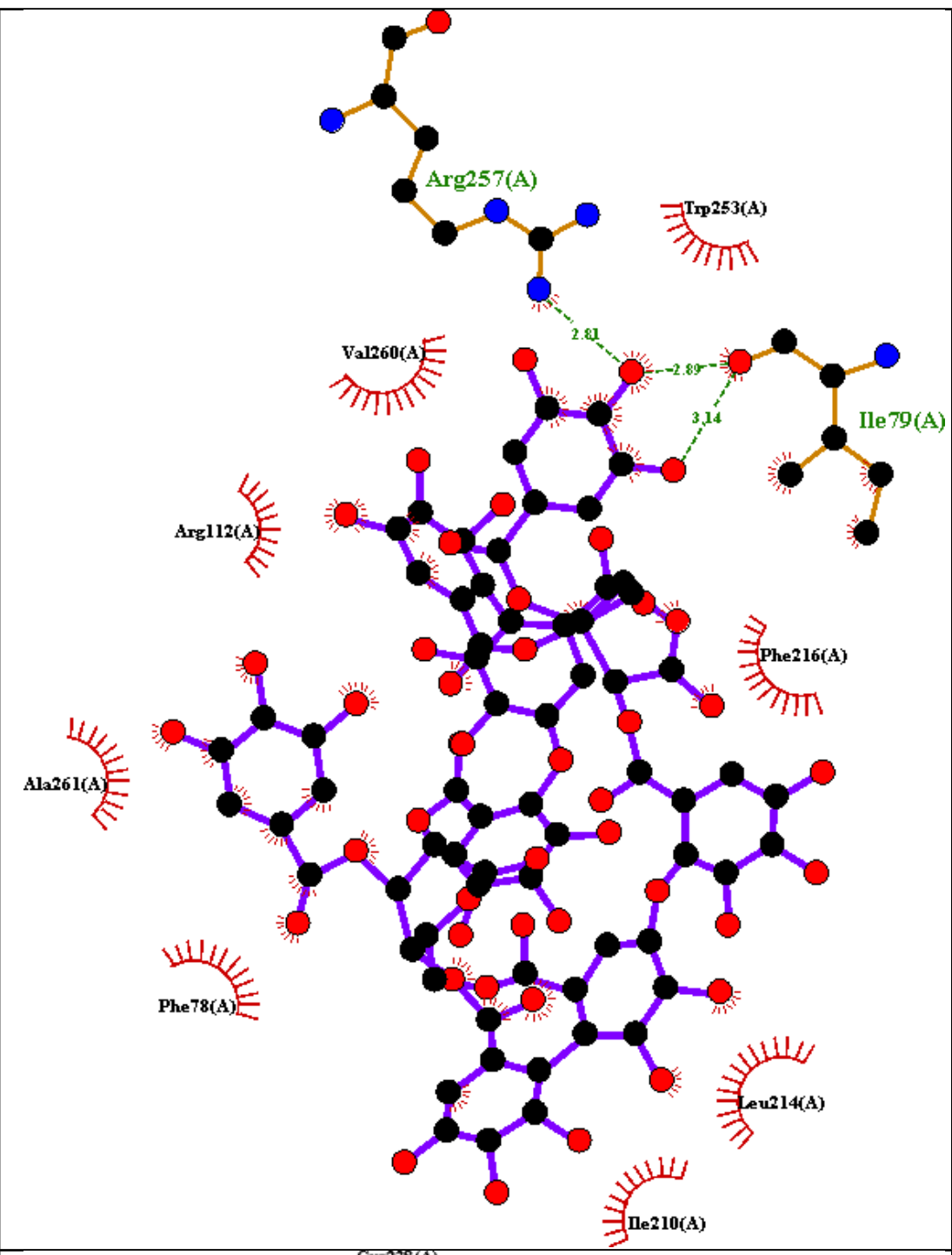	G77 F78 I79 D80 E84 R112 T113 Y115 H152 L154 A179 P181 D206 I210 I211 N213 L214 G215 F216 K233 C238 Q239 K240 N241 I242 Q245 I246 W253 T256 R257 D258 F259 V260 A261 C262 N263 L265	-7.5
5	Vescalagin	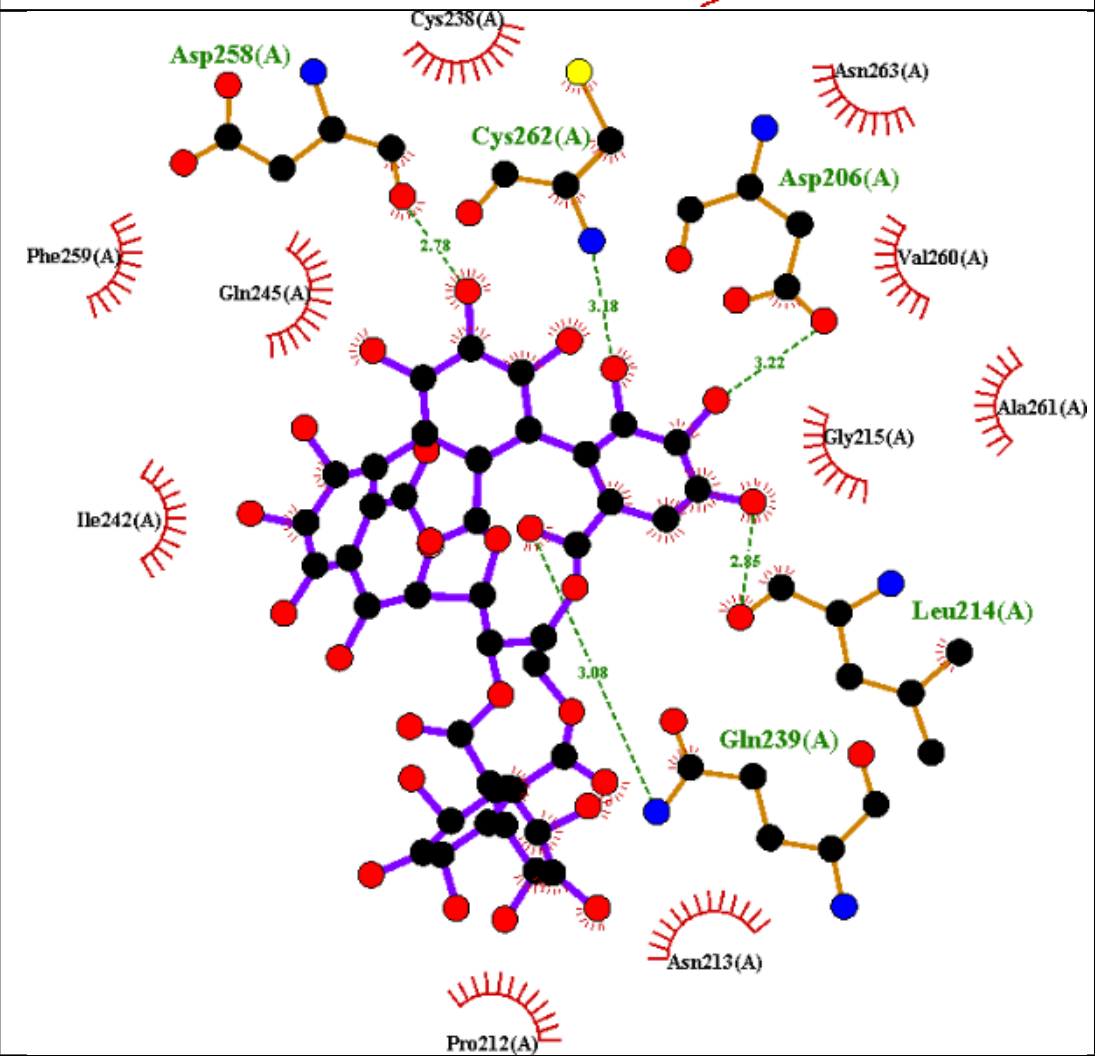	F78 I79 D80 K81 E84 R112 T113 G114 Y115 D206 A207 A208 P212 N213 L214 G215 F216 K233 Q234 C238 Q239 K240 N241 I242 Q245 W253 T256 R257 D258 F259 V260 A261 C262 N263	-8.7
6	Castalagin	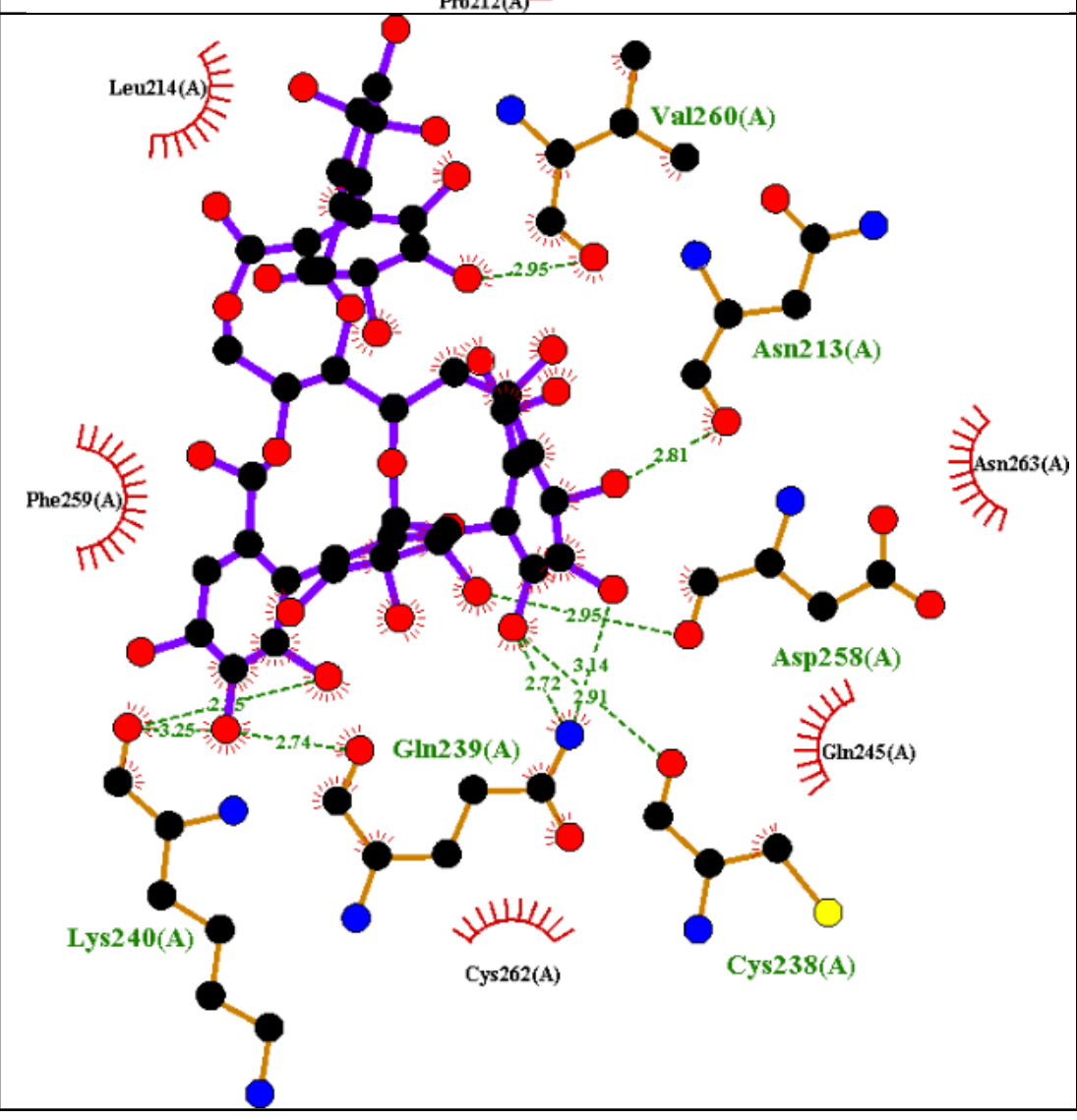	D206 A207 A208 P212 N213 L214 G215 F216 K233 Q234 G237 C238 Q239 K240 N241 I242 Q245 R257 D258 F259 V260 A261 C262 N263	-9.3

7	Rugosin E	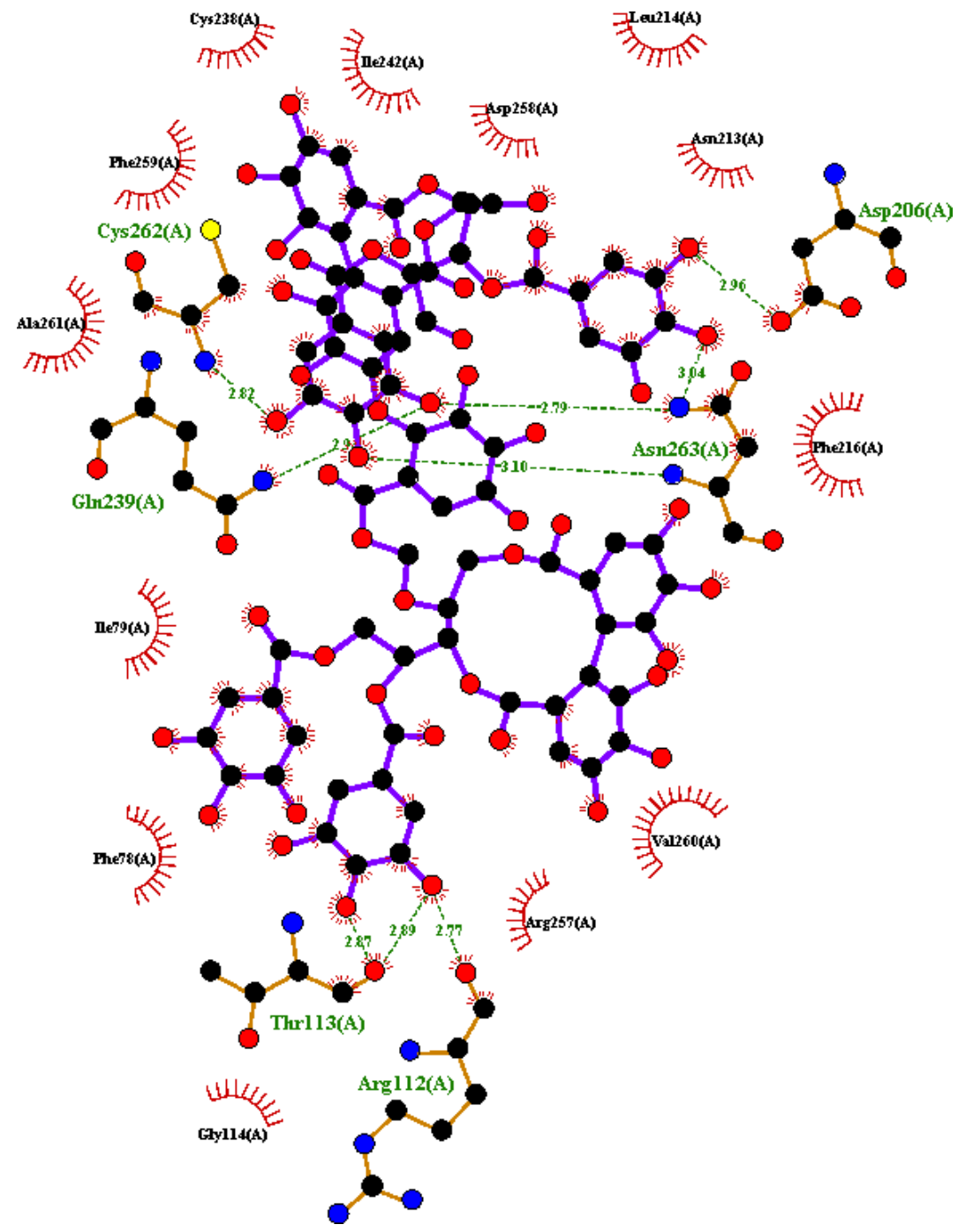	G77 F78 I79<br>D80 K81 E84<br>R112 T113<br>G114 Y115<br>H152 L154<br>L176 D177<br>P178 A179<br>E180 P181<br>D206 A207<br>A208 I210<br>P212 N213<br>L214 G215<br>F216 G217<br>K233 Q234<br>C238 Q239<br>K240 N241<br>I242 Q245 I246<br>I252 W253<br>T256 R257<br>D258 F259<br>V260 A261<br>C262 N263<br>L265 S267<br>Y268	-7.5
8	Tellimagrandin II	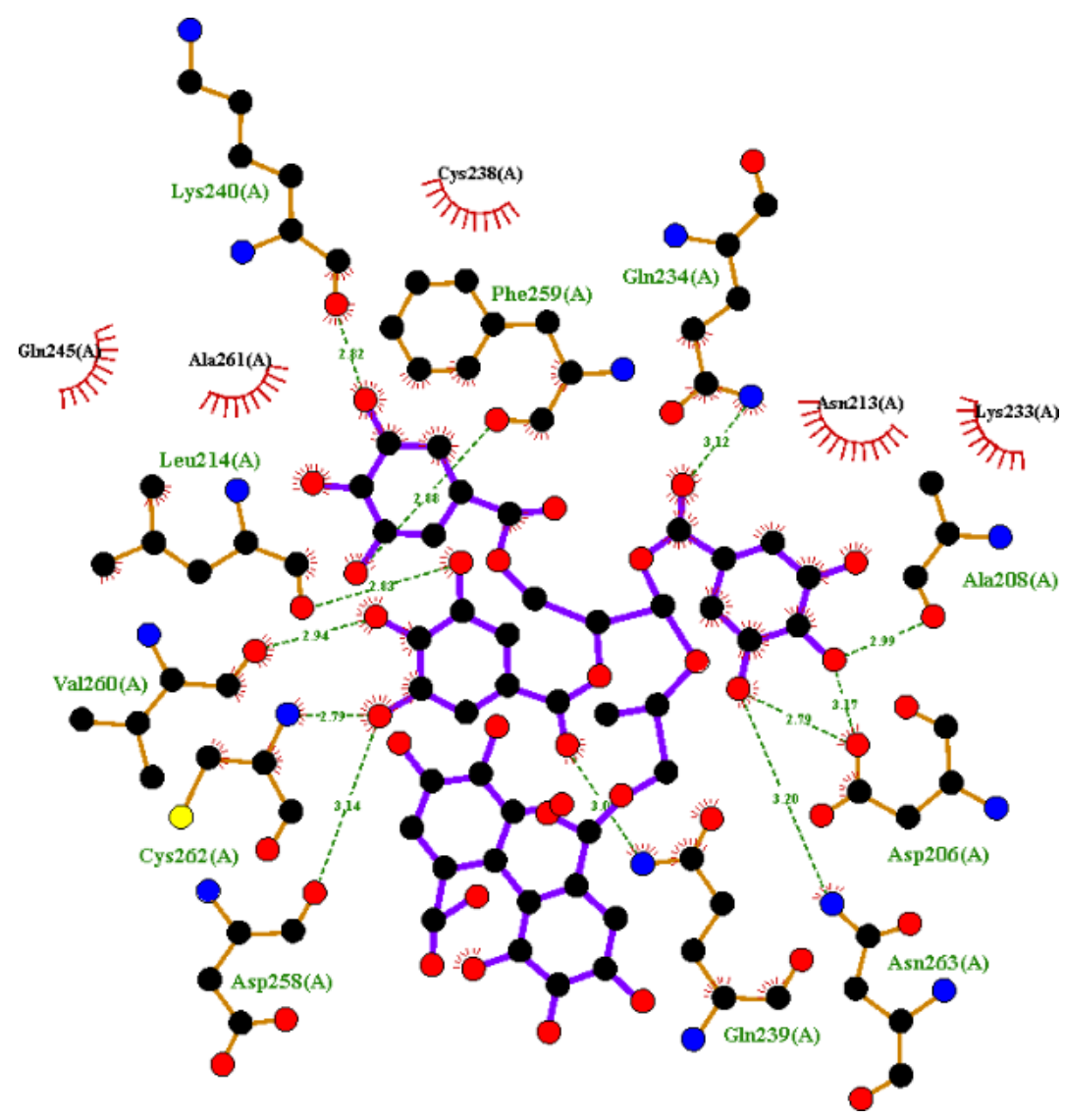	G77 F78 I79<br>D80 Y115 T116<br>H152 L154<br>P178 A179<br>E180 P181<br>D206 A207<br>A208 P209<br>I210 P212<br>N213 L214<br>G215 F216<br>G232 K233<br>Q234 G237<br>C238 Q239<br>K240 N241<br>I242 Q245<br>R257 D258<br>F259 V260<br>A261 C262<br>N263 L265<br>Y268	-9.1

9	Galloylglucose	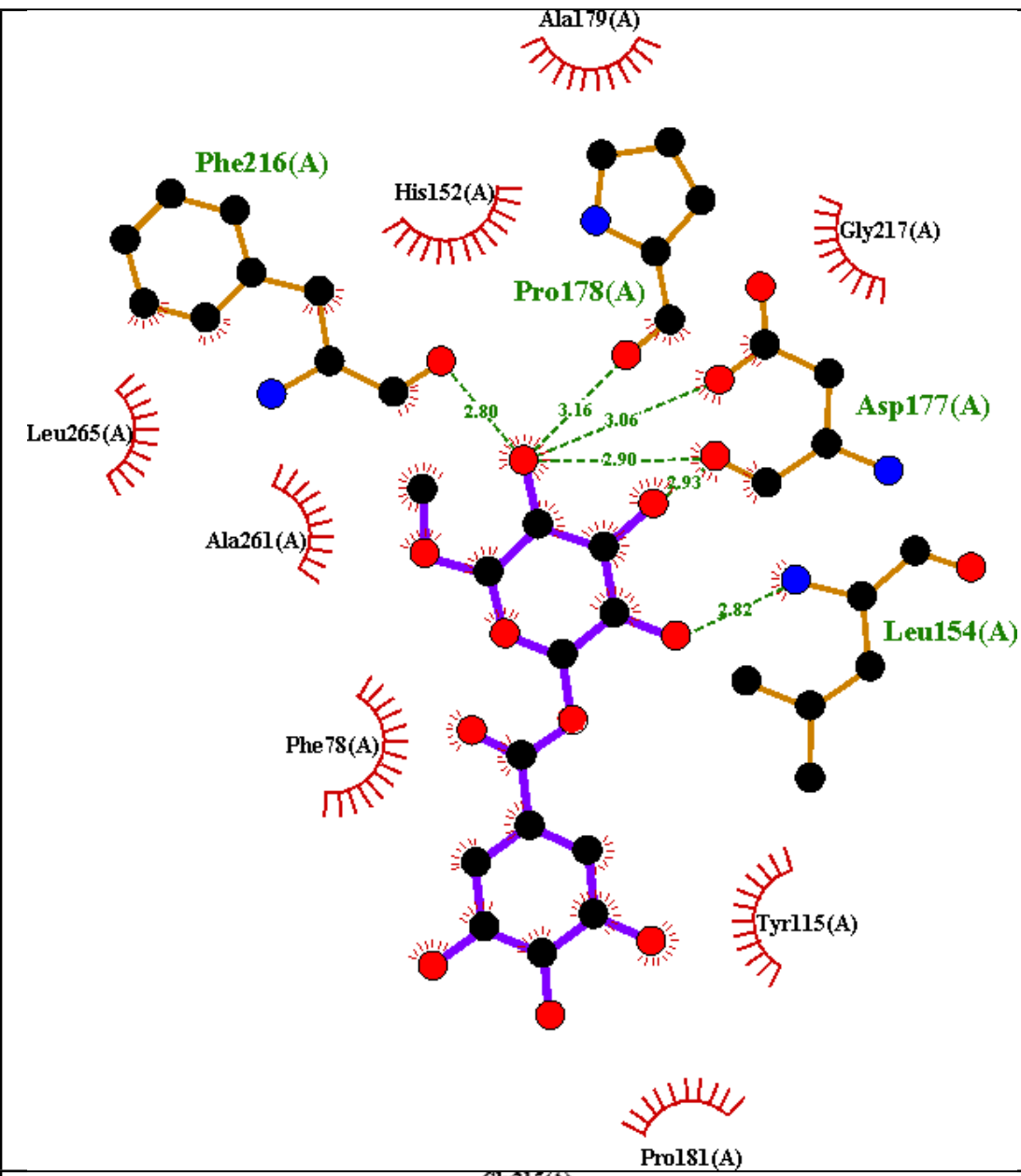	H76 G77 F78 I79 D80 Y115 H152 L154 G155 S156 L176 D177 P178 A179 E180 P181 D206 A207 A208 P209 I210 N213 L214 G215 F216 G217 K233 Q234 C238 Q239 K240 N241 I242 Q245 R257 D258 F259 V260 A261 C262 N263 L265 S267 Y268	-8.3
10	Vescavaloninic acid	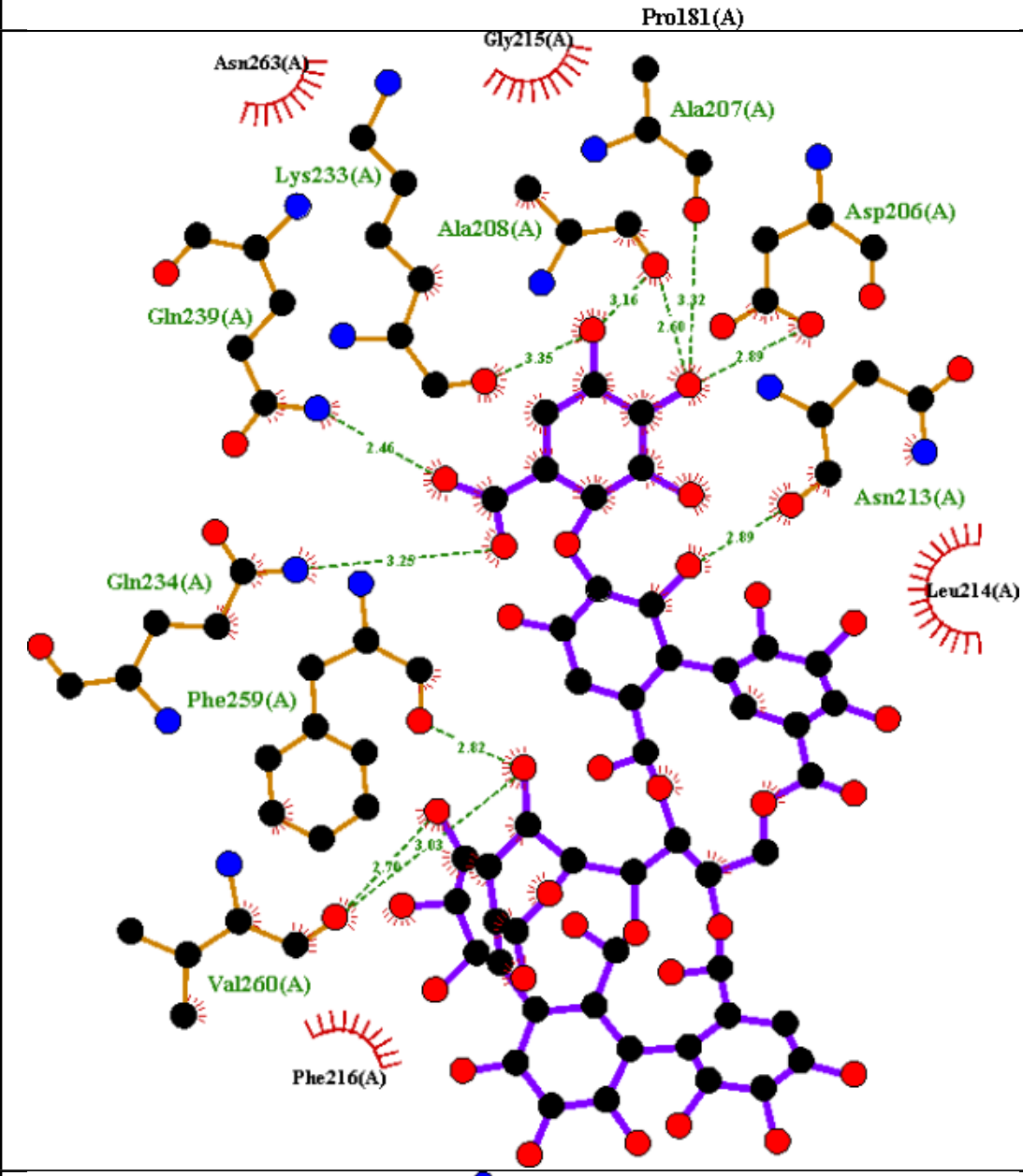	F78 I79 Y115 P181 D206 A207 A208 P209 I210 P212 N213 L214 G215 F216 K233 Q234 C238 Q239 K240 N241 I242 Q245 I246 I252 W253 T256 D258 F259 V260 A261 C262 N263	-7.1
11	Pentagalloylglucose	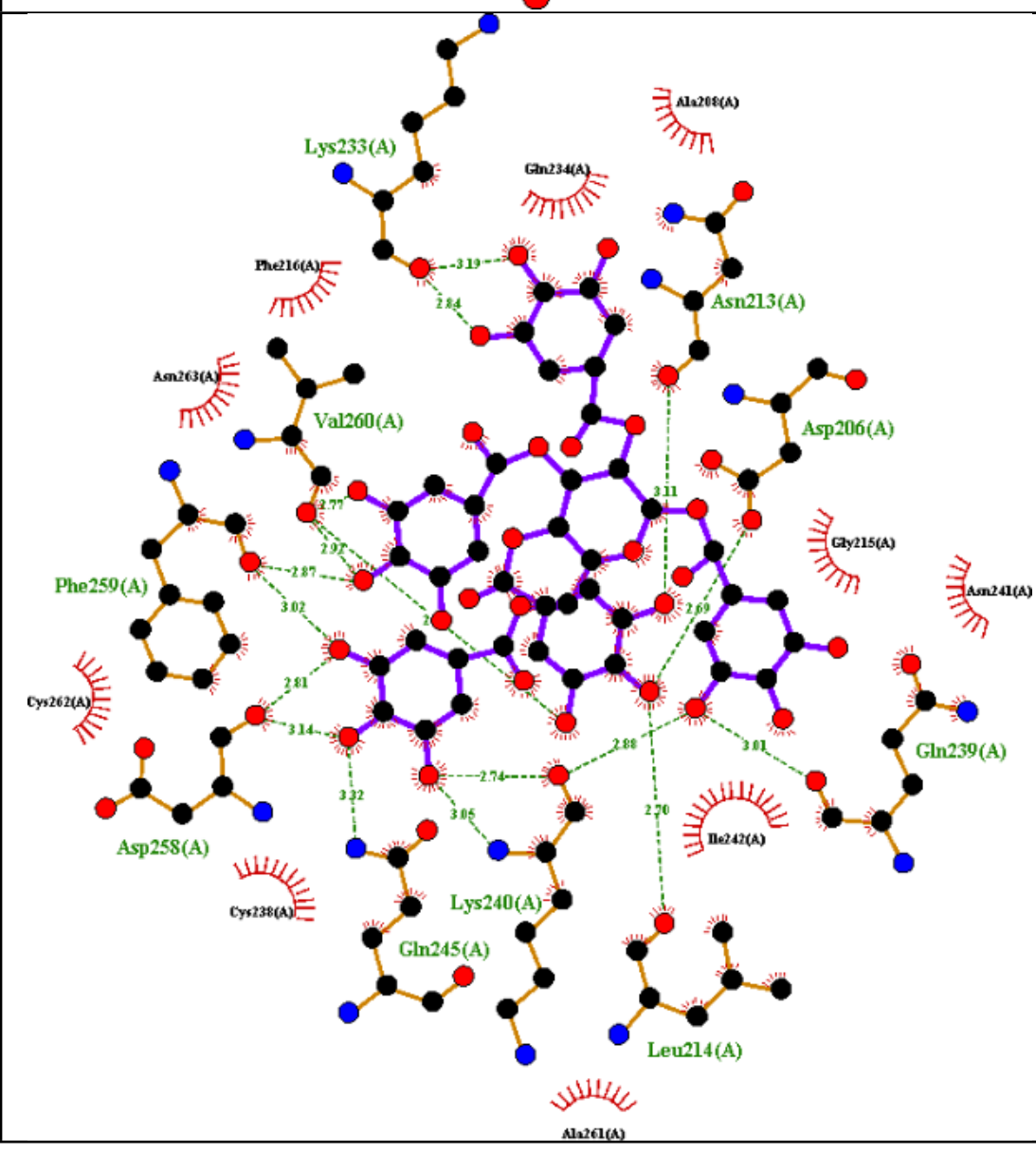	D206 A207 A208 P209 P212 N213 L214 G215 F216 K233 Q234 C238 Q239 K240 N241 I242 Q245 R257 D258 F259 V260 A261 C262 N263	-8.2

12	Orlistat (control)	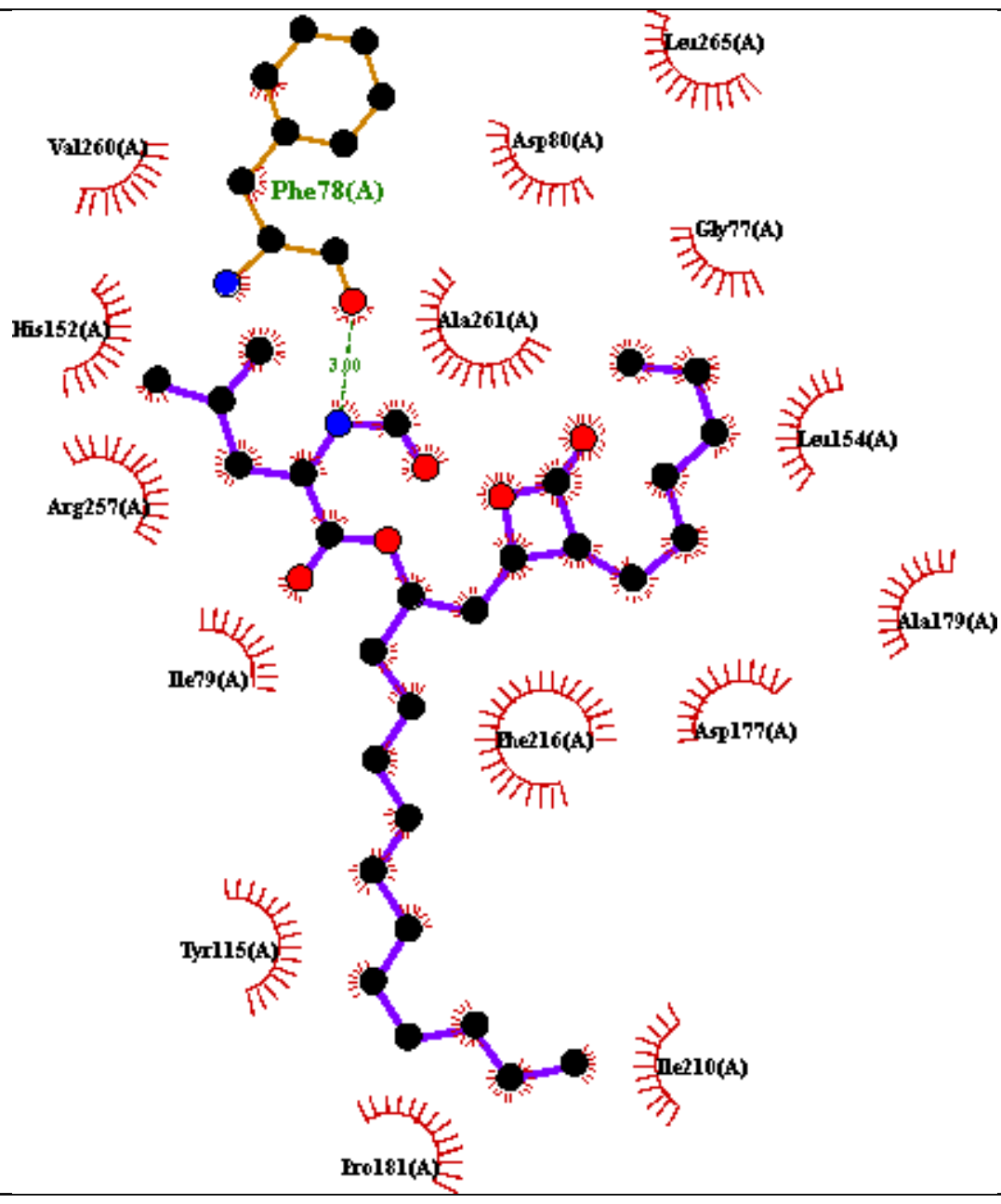	H76 G77 F78 I79 D80 W86 Y115 H152 L154 G155 D177 P178 A179 E180 P181 T205 D206 I210 F216 G217 W253 T256 R257 V260 A261 N263 L265 R266 S267 Y268	-7.8
α-Amylase (3BAY)				
No.	Compound	2D interactions	Contact residues	Affinity [kcal/mo]
1	Ellagic acid	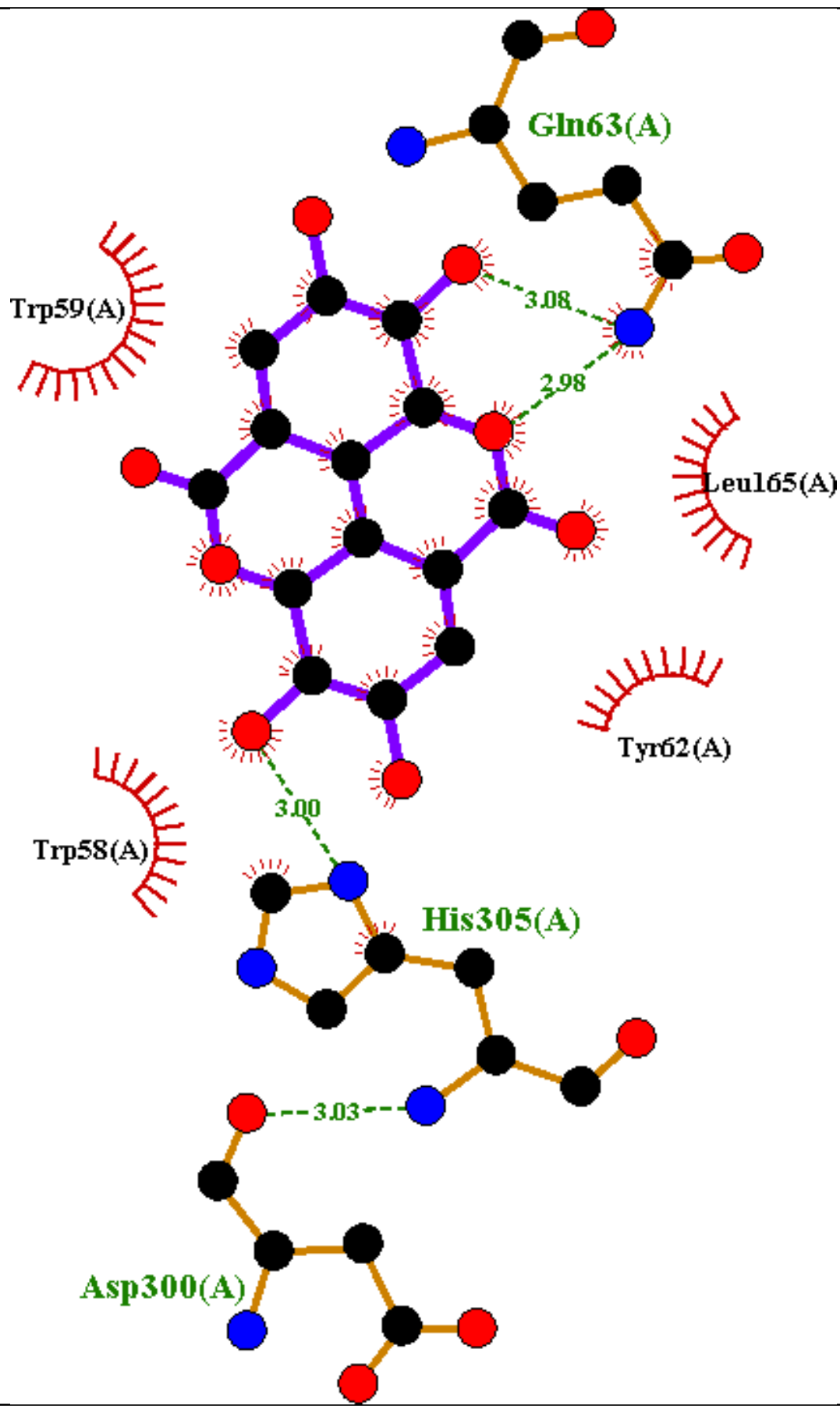	I51 W58 W59 Y62 Q63 V98 H101 G104 V107 Y151 L162 T163 L165 R195 D197 A198 S199 K200 H201 E233 V234 I235 E240 H299 D300 H305	-8.5

2	Gallic acid	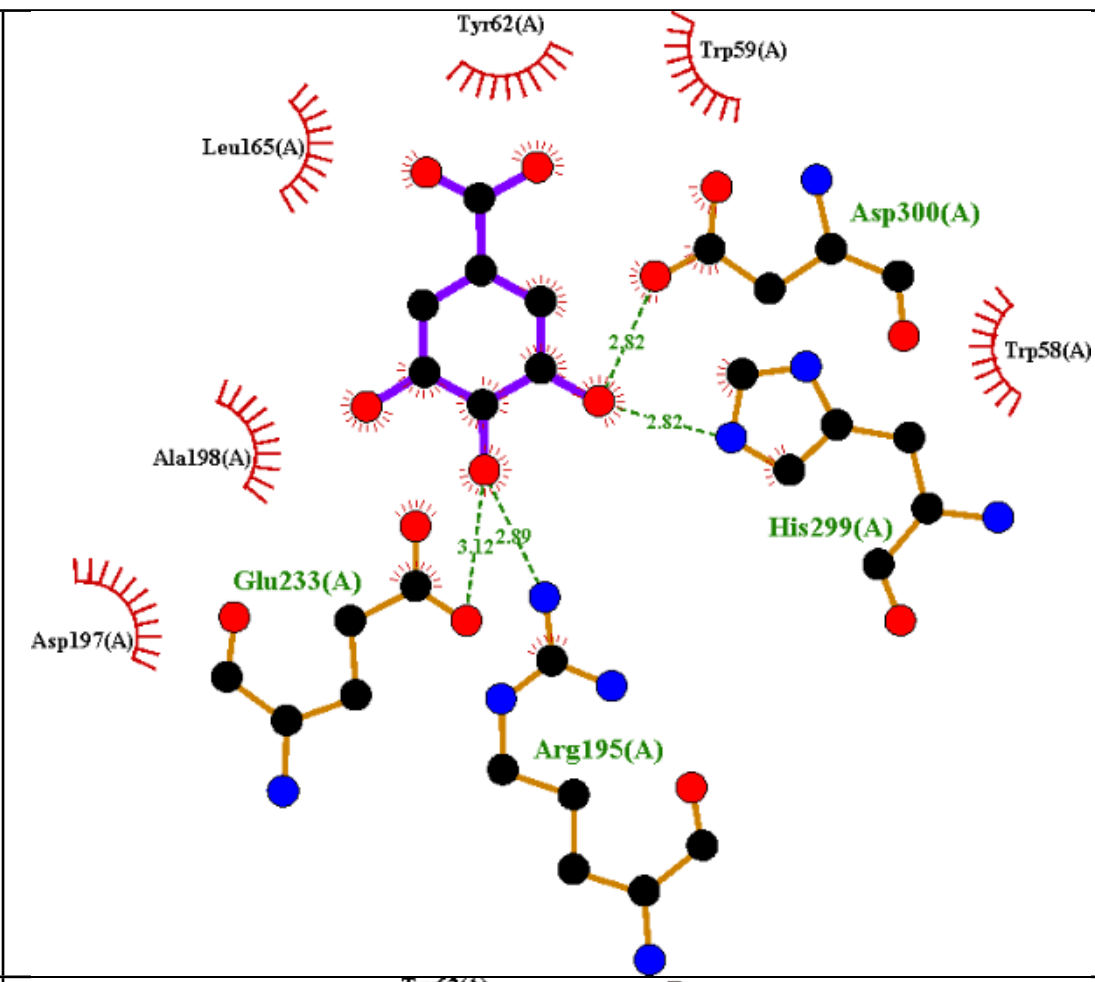	W58 W59 E60 R61 Y62 Q63 V98 H101 G104 Y151 L162 T163 L165 R195 D197 A198 S199 K200 H201 E233 I235 H299 D300 H305 G306	-6.2
3	Cretanin	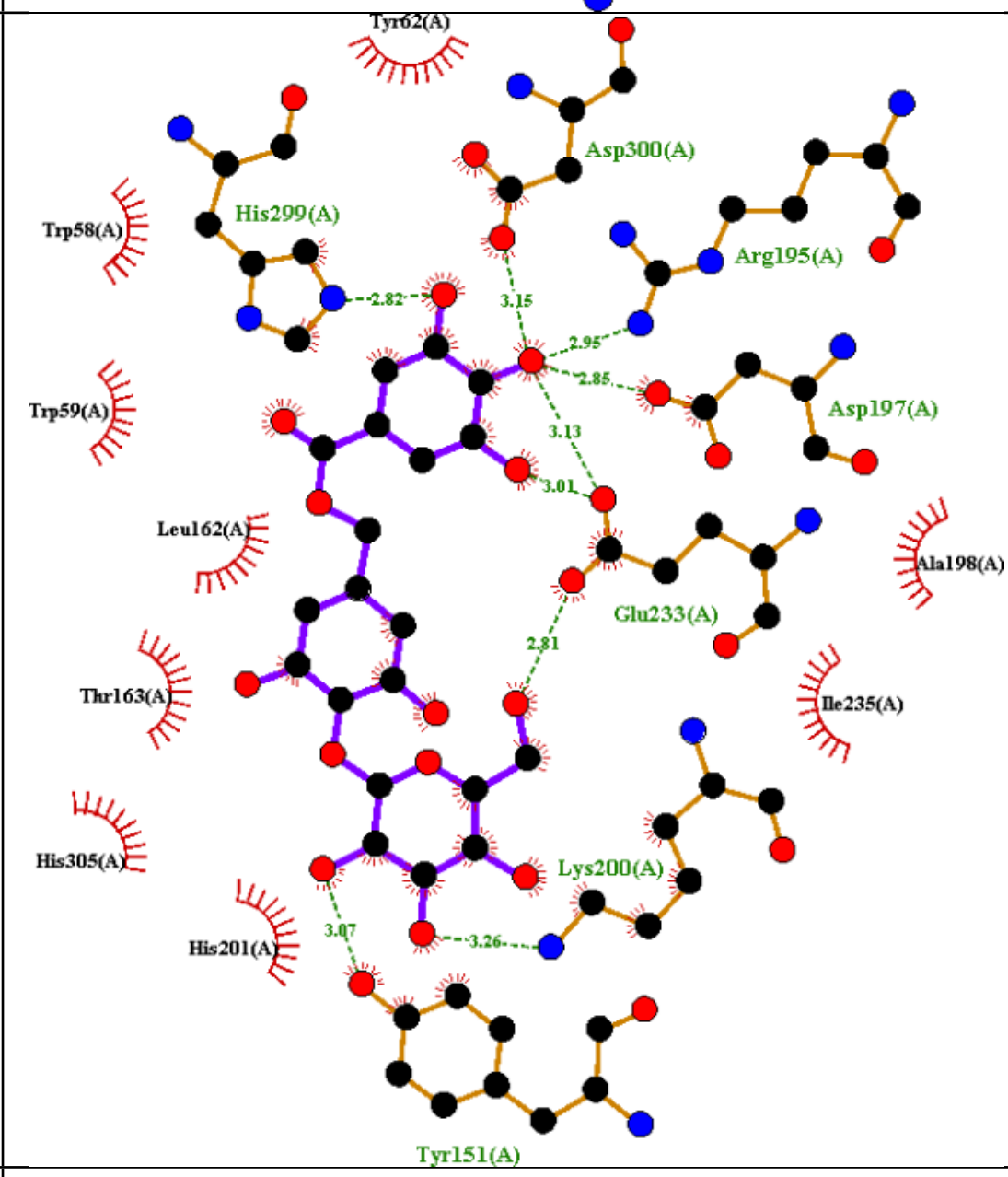	W58 W59 E60 Y62 Q63 V98 H101 Y151 L162 T163 L165 R195 D197 A198 S199 K200 H201 E233 V234 I235 D236 E240 H299 D300 H305 G306	-8.5
4	Oenothein B	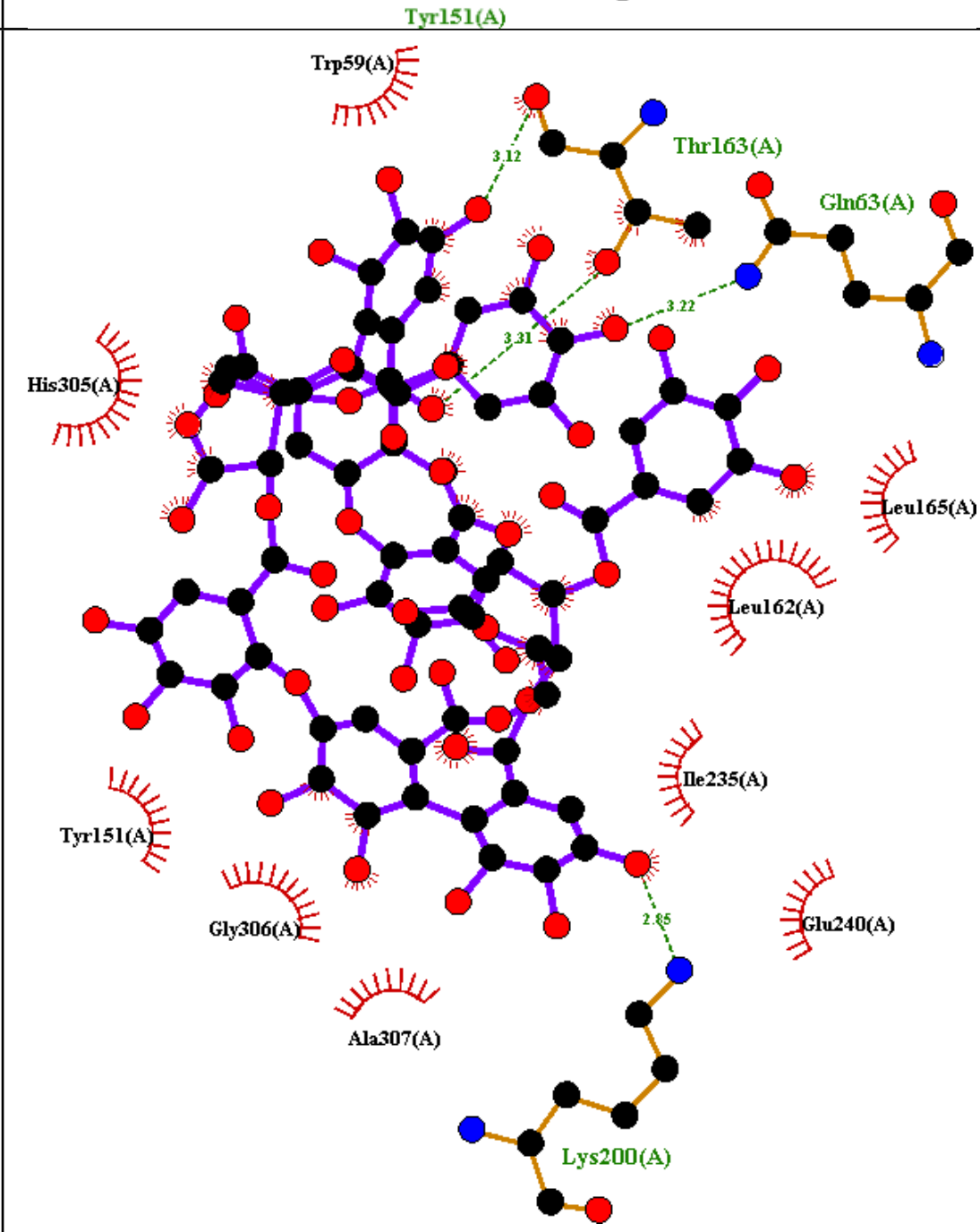	I51 N53 W58 W59 Y62 Q63 H101 N105 A106 V107 S145 D147 I148 E149 N150 Y151 N152 L162 T163 G164 L165 R195 D197 A198 K200 H201 E233 I235 D236 L237 G238 G239 E240 P241 H299 D300 R303 G304 H305 G306 A307 G308 G309 A310 N352 D353 D356	-8.6

5	Vascalagin	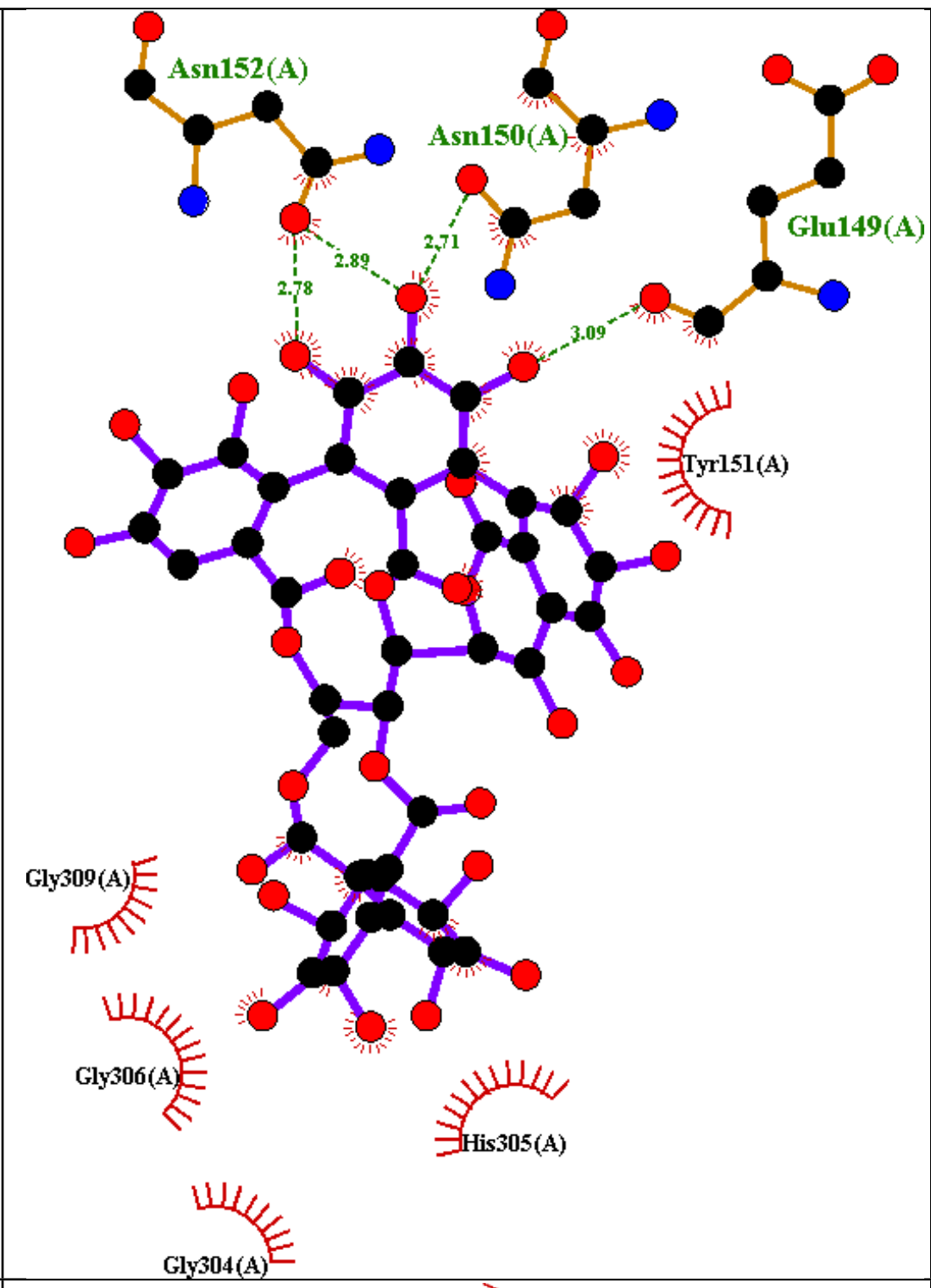	I148 E149<br>N150 Y151<br>N152 V157<br>L162 T163<br>A198 K200<br>H201 E233<br>V234 I235<br>D236 L237<br>G238 G239<br>E240 P241<br>D300 G304<br>H305 G306<br>A307 G308<br>G309 A310<br>D356	-7.4
6	Castalagin	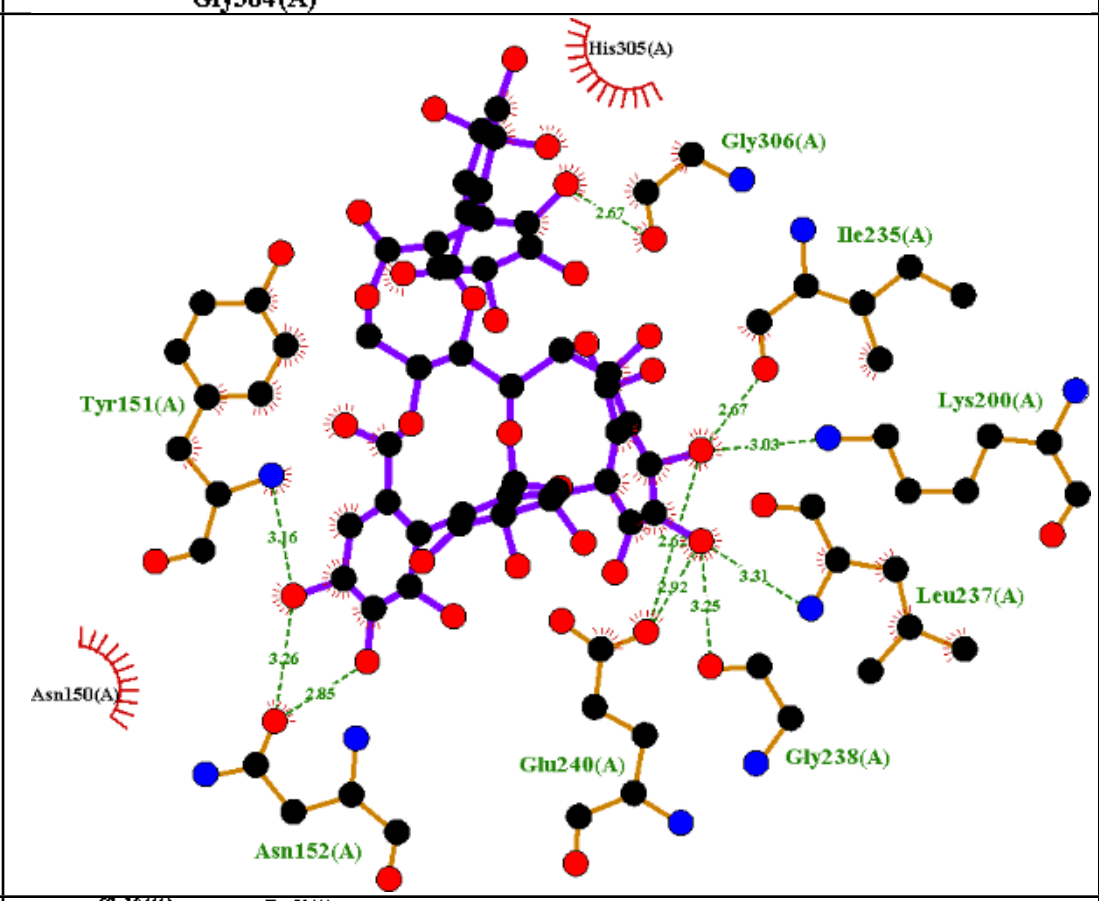	W58 I148 E149<br>N150 Y151<br>N152 L162<br>T163 L165<br>R195 D197<br>A198 K200<br>H201 E233<br>I235 D236<br>L237 G238<br>G239 E240<br>D300 G304<br>H305 G306<br>A307 G308<br>G309 A310<br>S311 D356	-8.1
7	Rugosin E	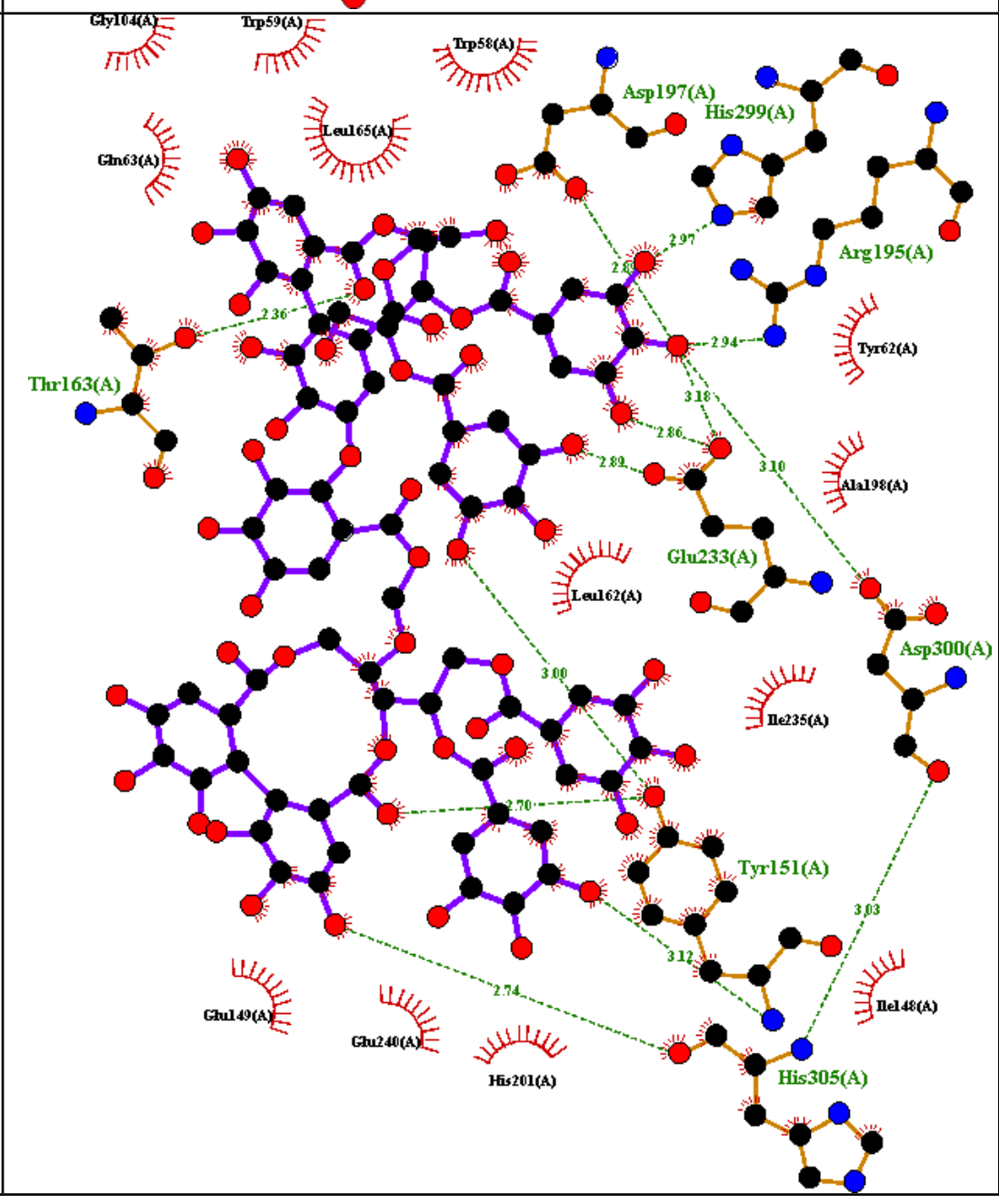	I51 P54 W58<br>W59 E60 R61<br>Y62 Q63 H101<br>C103 G104<br>N105 A106<br>V107 S145<br>D147 I148<br>E149 N150<br>Y151 N152<br>R161 L162<br>T163 G164<br>L165 R195<br>D197 A198<br>S199 K200<br>H201 E233<br>V234 I235<br>D236 L237<br>G238 G239<br>E240 P241<br>H299 D300<br>R303 G304<br>H305 G306	-9.0

			A307 G308 G309 A310 S311 G351 N352 D353 V354 D356 W357	
8	Tellimagrandin II	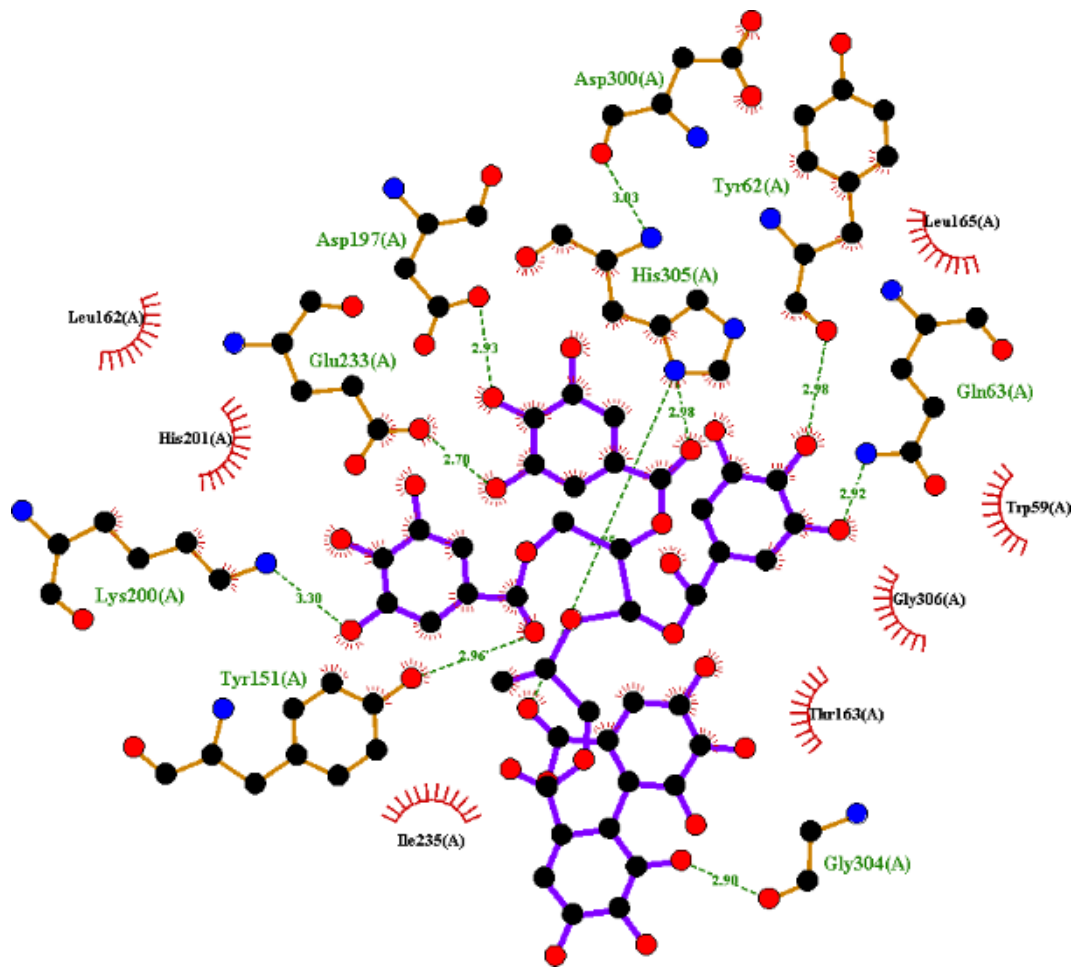	W58 W59 E60 R61 Y62 Q63 H101 G104 D147 I148 E149 Y151 R161 L162 T163 G164 L165 R195 D197 A198 S199 K200 H201 E233 V234 I235 D236 L237 G238 G239 E240 H299 D300 G304 H305 G306 A307 G308 G309 N352 D356	-9.4
9	Galloylglucose	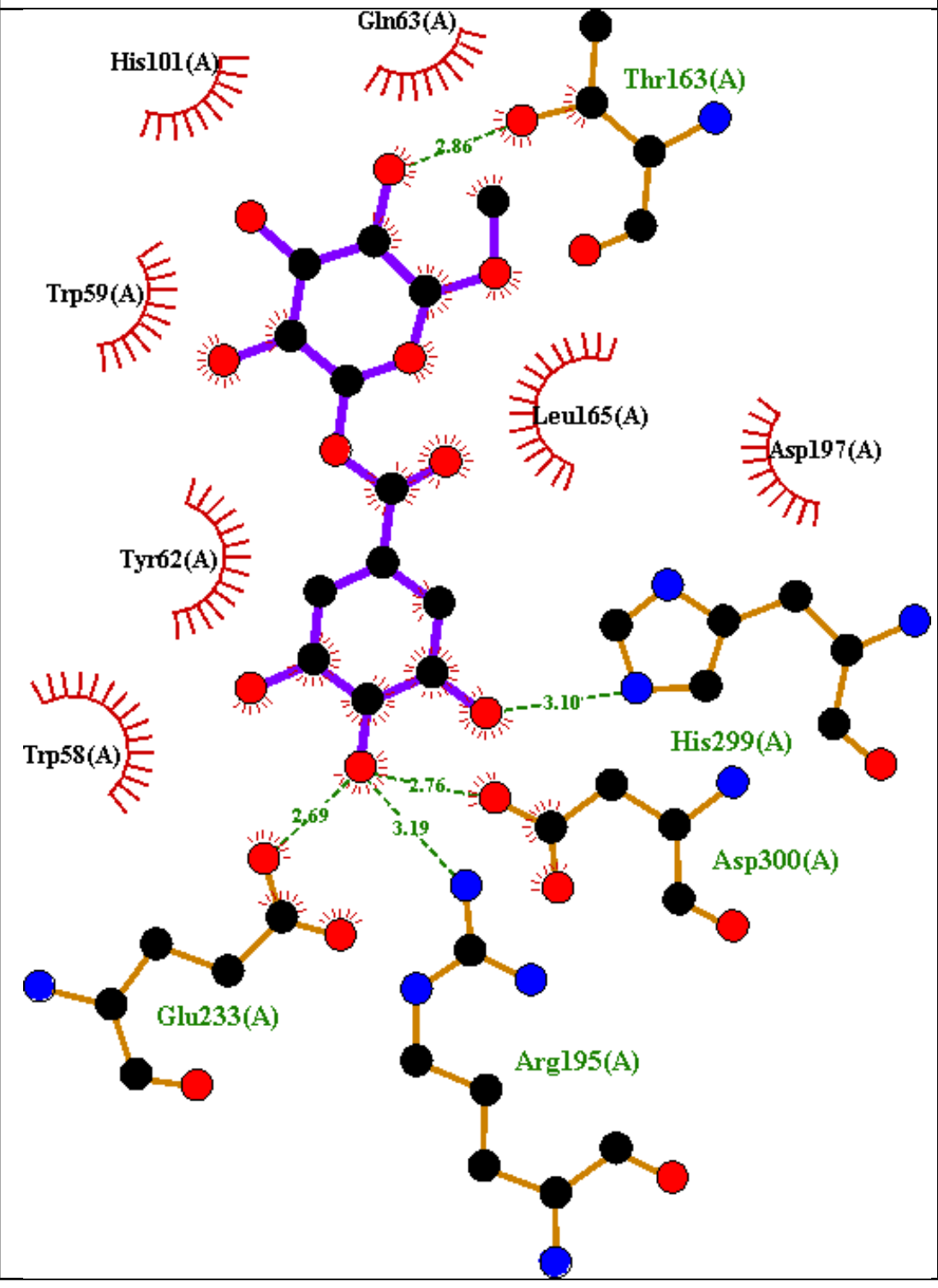	I51 W58 W59 Y62 Q63 H101 Y151 L162 T163 L165 R195 D197 A198 K200 H201 E233 I235 E240 H299 D300 H305 G306	-7.6

10	Vescavaloninic acid	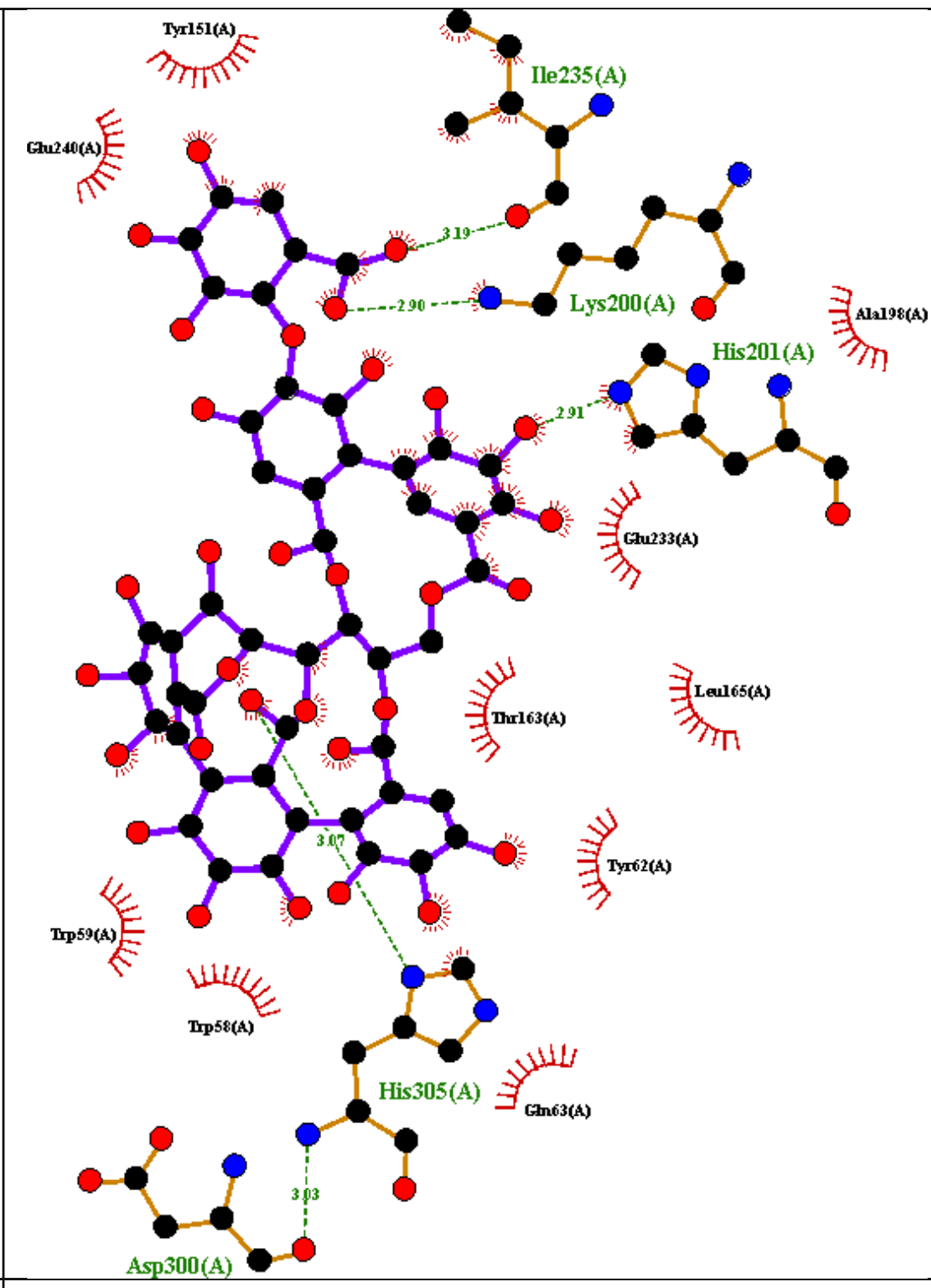	I51 N53 P54 W58 W59 E60 Y62 Q63 H101 C103 G104 N105 A106 V107 D147 I148 E149 N150 Y151 N152 L162 T163 G164 L165 R195 D197 A198 S199 K200 H201 E233 I235 D236 L237 G238 G239 E240 H299 D300 R303 G304 H305 G306 A307 G308 G309 D353 V354 D356 W357	-10.2
11	Pentagalloylglucose	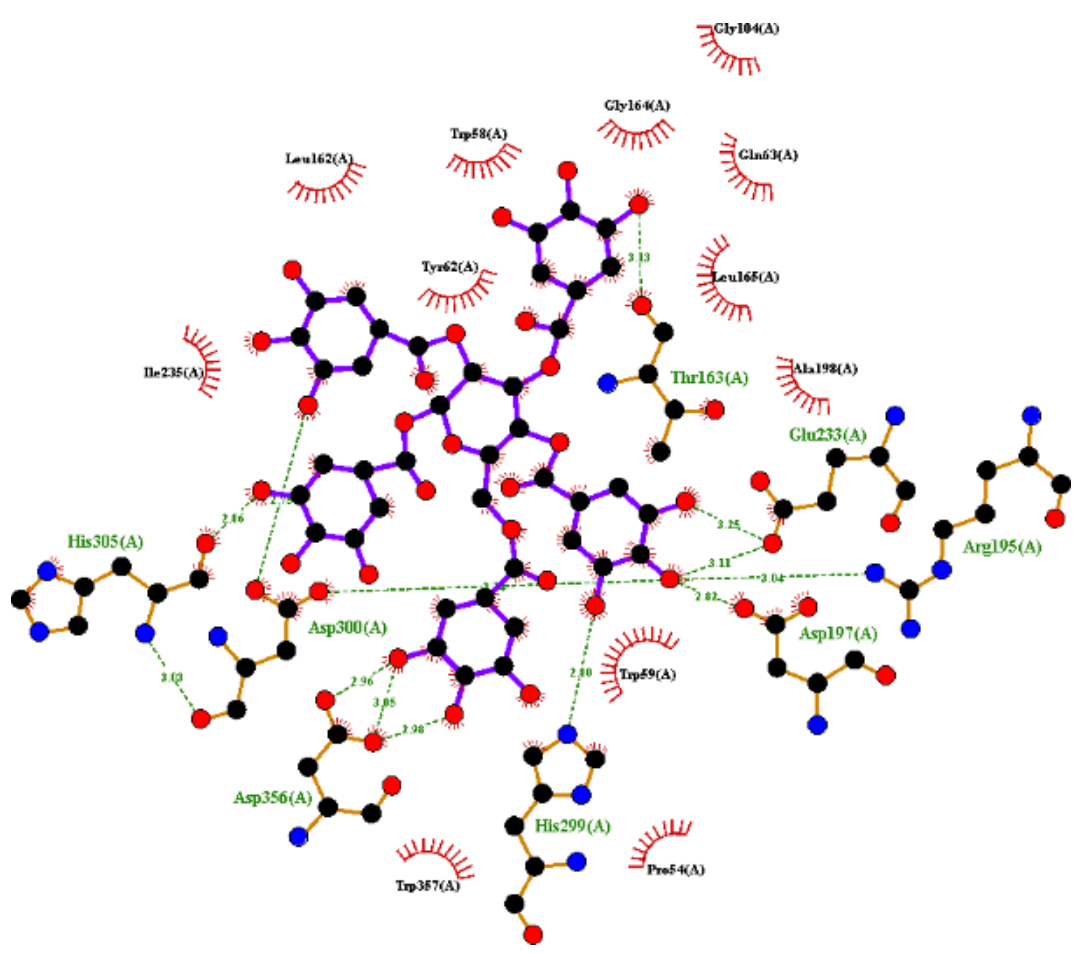	I51 N53 P54 W58 W59 Y62 Q63 V98 H101 C103 G104 N105 A106 V107 Y151 L162 T163 G164 L165 R195 D197 A198 S199 K200 H201 E233 V234 I235 H299 D300 G304 H305 G306 A307 D353 V354 D356 W357	-9.6

<table>
<tr><td>12</td><td>Acarbose (control)</td><td>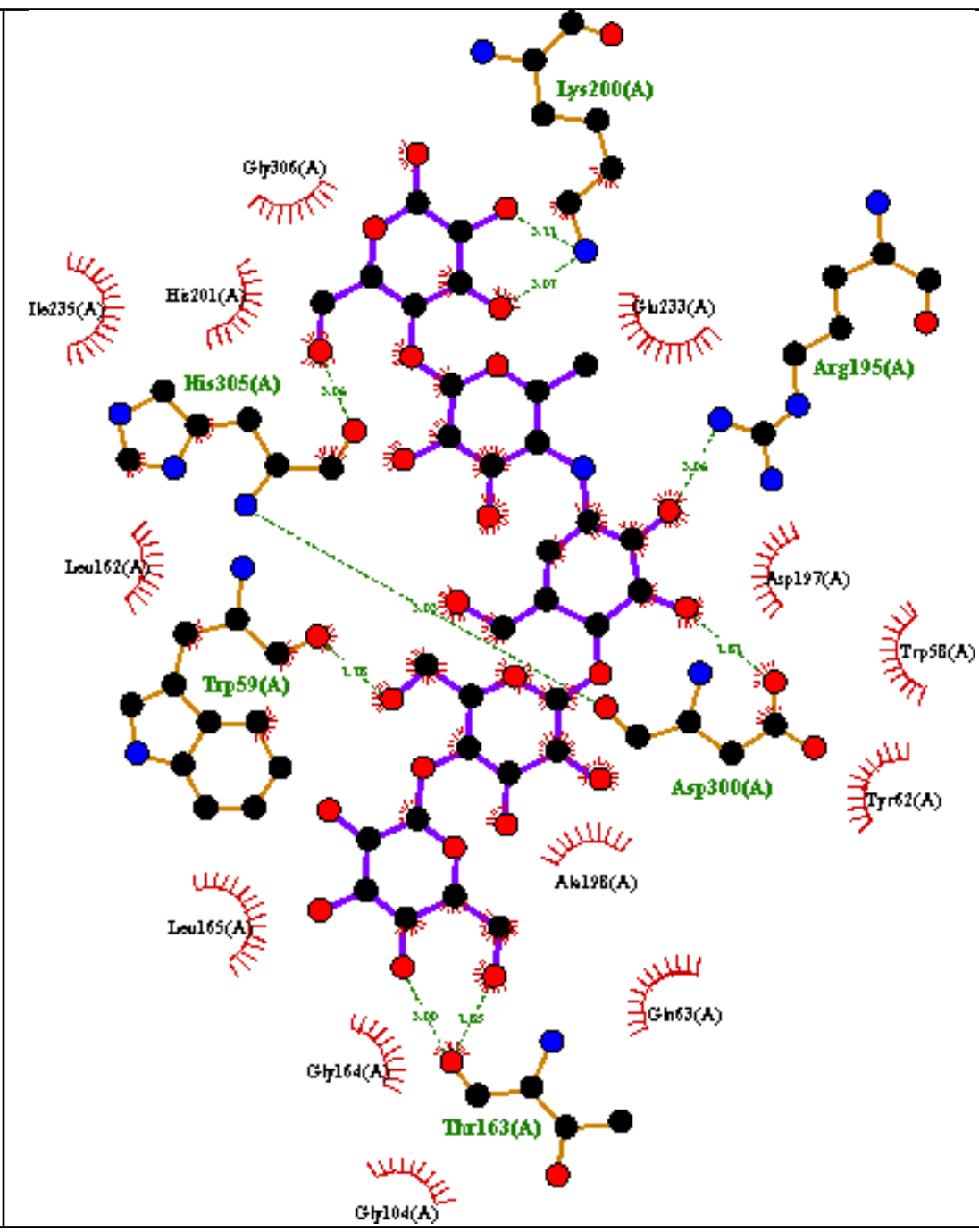
</td><td>I51 W58 W59 E60 R61 Y62 Q63 V98 H101 C103 G104 N105 A106 V107 S145 D147 I148 E149 Y151 L162 T163 G164 L165 R195 D197 A198 S199 K200 H201 E233 V234 I235 D236 L237 E240 H299 D300 H305 G306 A307 G308 N352 D353 V354 D356</td><td>-8.4</td></tr>
<tr><td colspan="5">Tyrosinase (2Y9W)</td></tr>
<tr><td>No.</td><td>Compound</td><td>2D interactions</td><td>Contact residues</td><td>Affinity [kcal/mol]</td></tr>
<tr><td>1</td><td>Ellagic acid</td><td>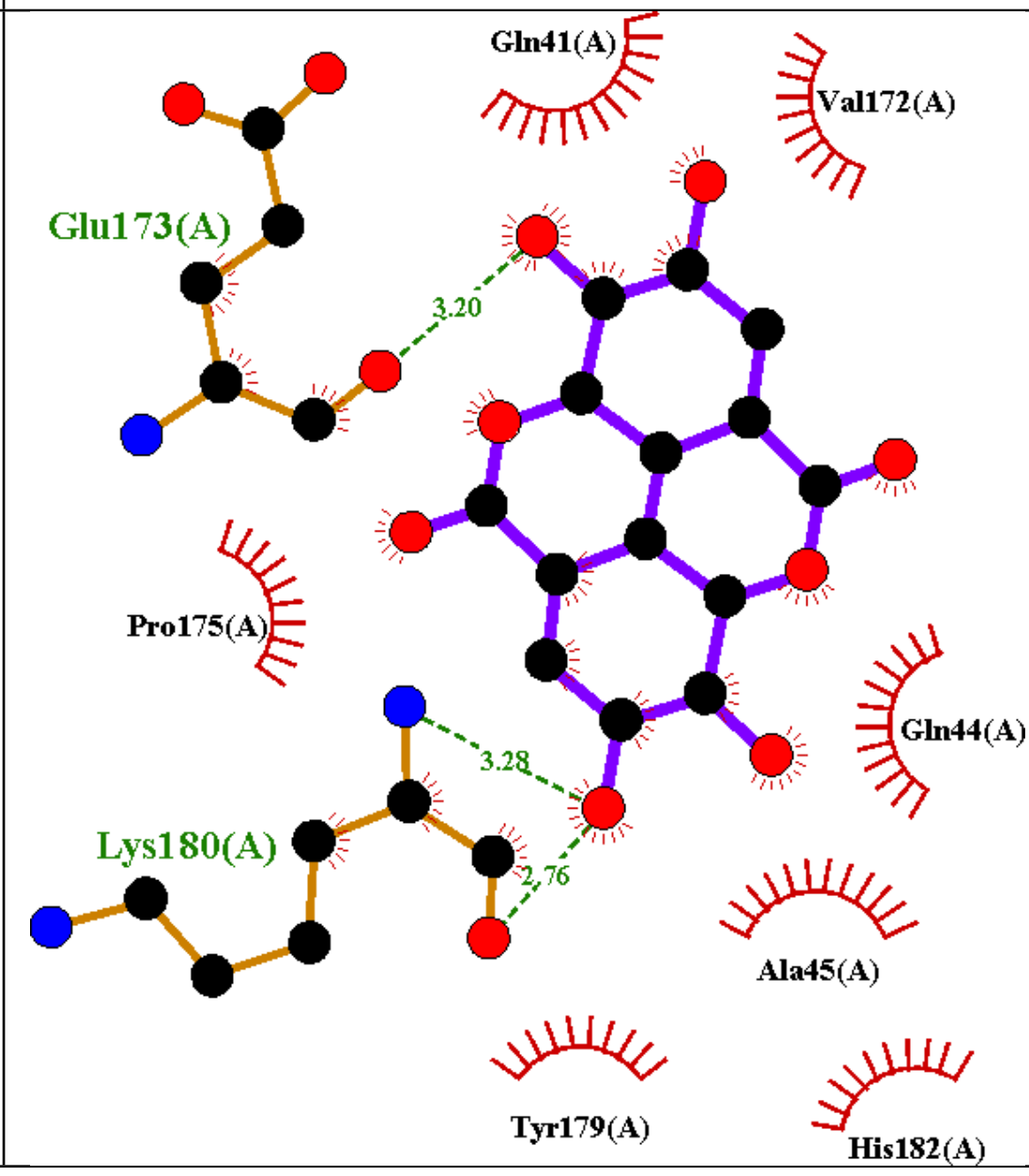
</td><td>Q41 Q44 A45 K158 V172 E173 N174 P175 I176 L177 H178 Y179 K180 F181 H182 T197</td><td>-6.7</td></tr>
</table>

2	Gallic acid	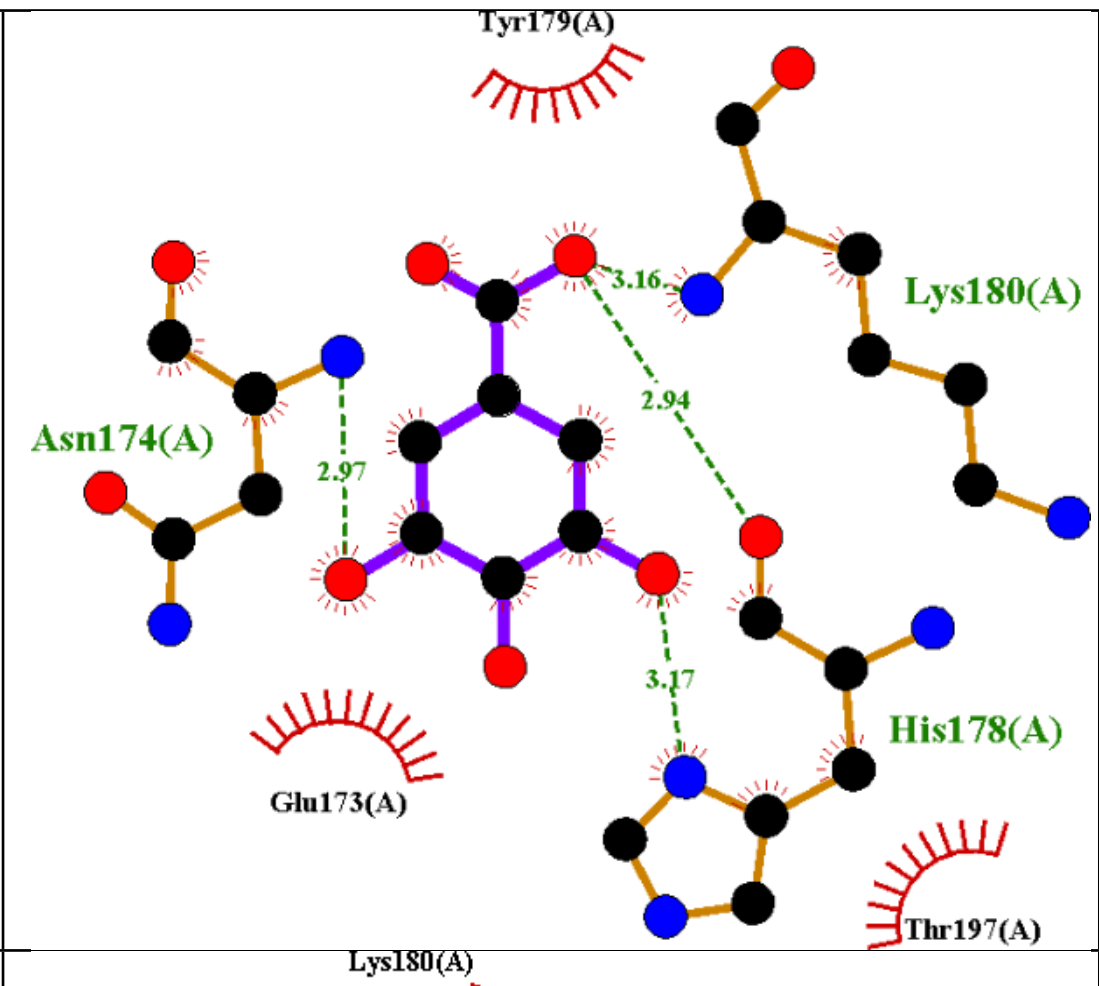	L40 Q41 V42 Q44 A45 D157 K158 V172 E173 N174 P175 L177 H178 Y179 K180 F181 H182 T197	-5.5
3	Cretanin	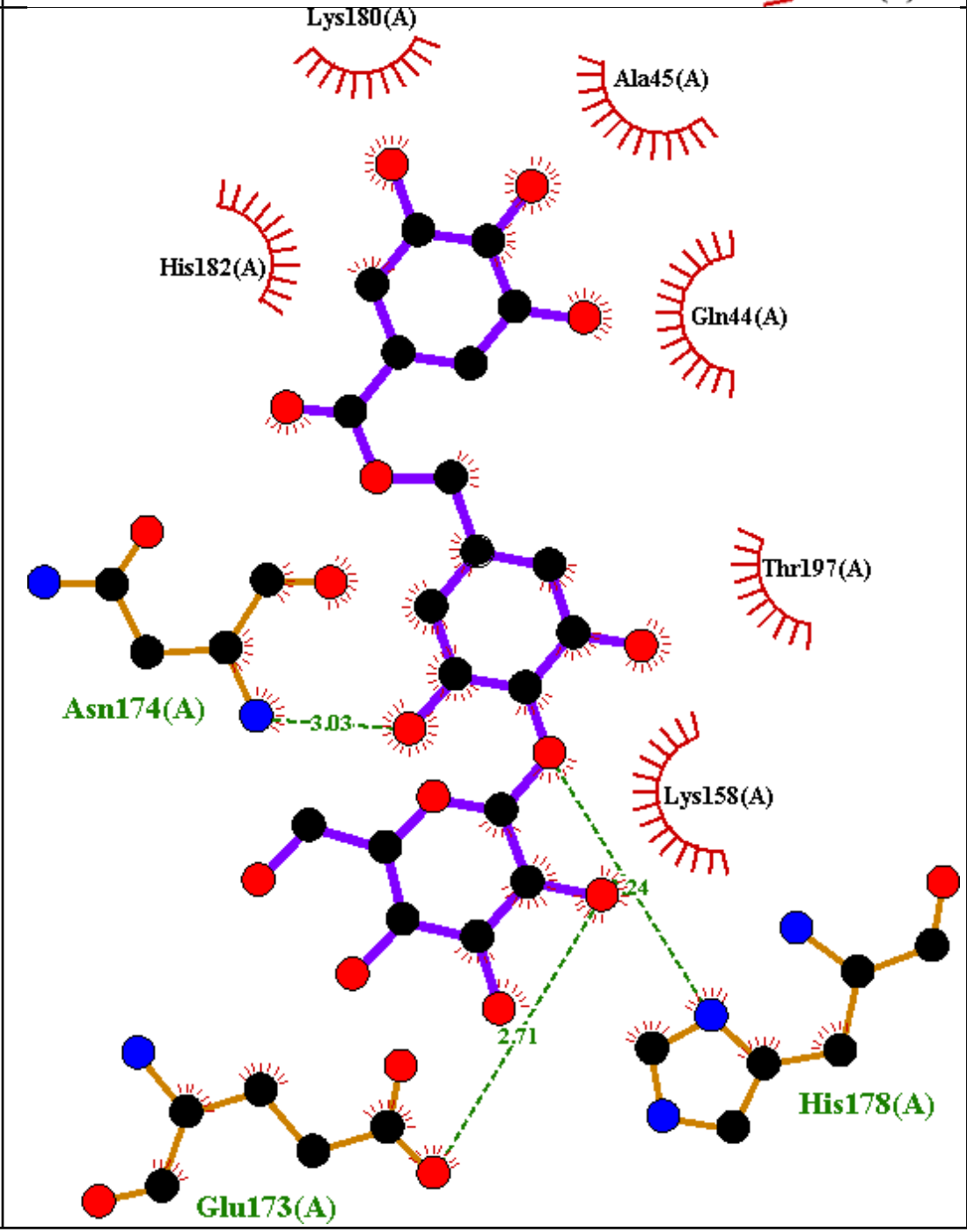	V37 R38 L40 Q41 V42 Q44 A45 R46 D157 K158 Q159 V172 E173 N174 P175 I176 L177 H178 Y179 K180 F181 H182 T197 T198 M199 E208	-7.1

4	Oenothein B	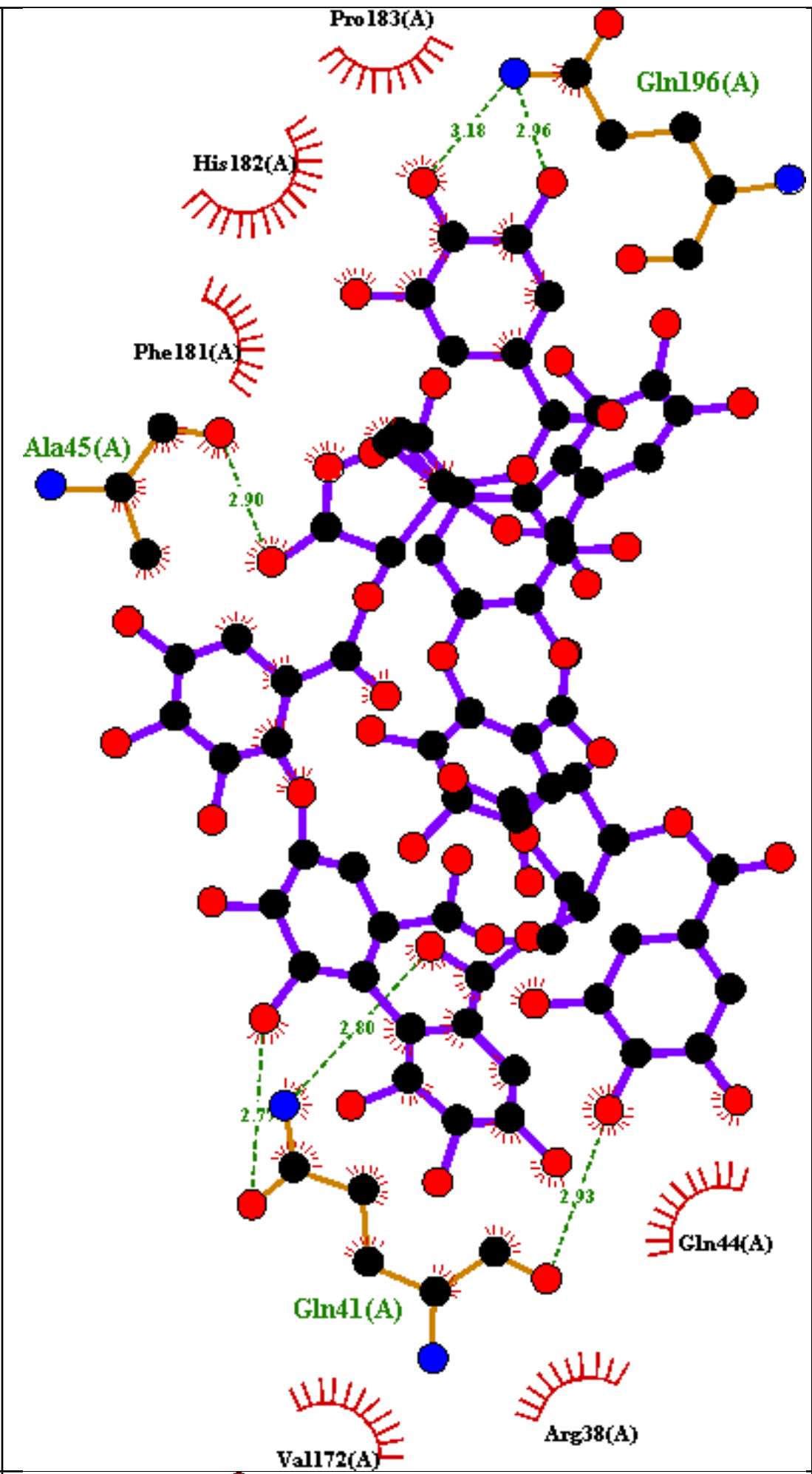	R38 L40 Q41 V42 Q44 A45 R46 D47 T111 V112 R115 D157 K158 Q159 D164 Y165 I170 E171 V172 E173 N174 P175 L177 H178 Y179 K180 F181 H182 P183 I184 Q196 T197	-7.2
5	Vescalagin	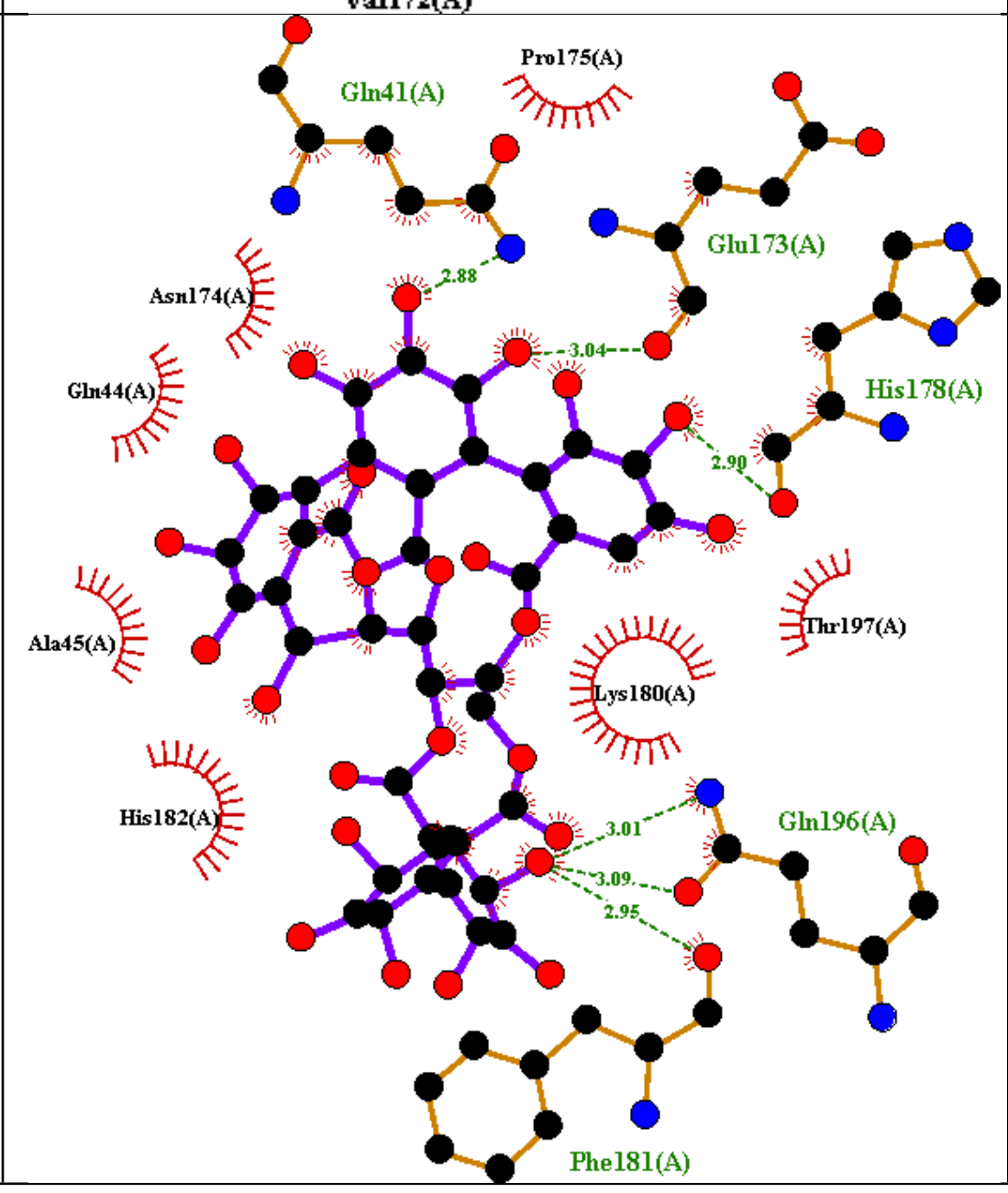	R38 L40 Q41 V42 Q44 A45 R46 K158 Q159 Y165 I170 E171 V172 E173 N174 P175 L177 H178 Y179 K180 F181 H182 P183 I184 Q194 W195 Q196 T197 M199 K206 Q207 E208 I210	-8.1

6	Castalagin	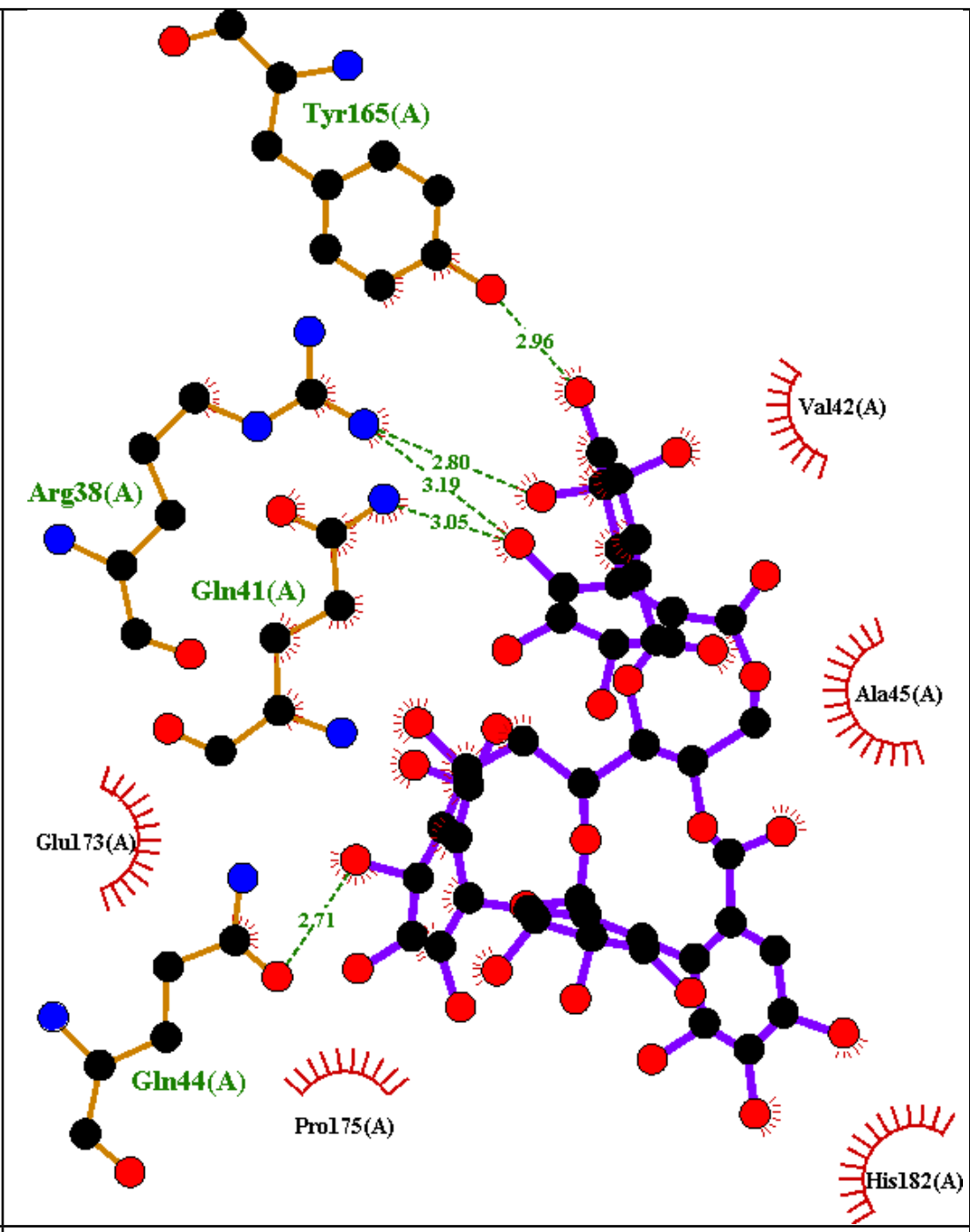	V37 R38 L40 Q41 V42 Q44 A45 R46 D47 V112 R115 Q159 I162 D164 Y165 I170 E171 V172 E173 N174 P175 H178 Y179 K180 F181 H182 P183 I184 Q196	-8.3
7	Rugosin E	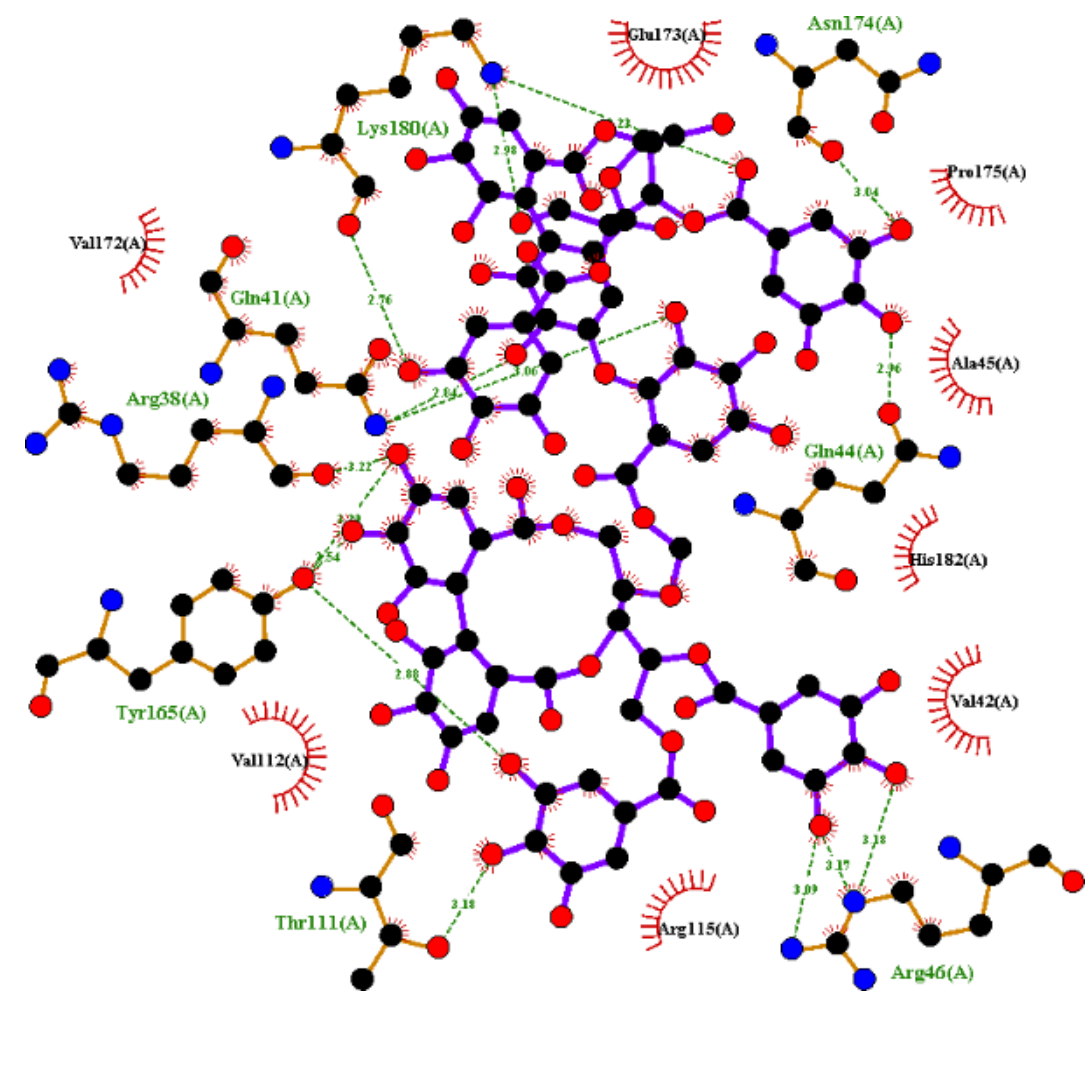	V37 R38 A39 L40 Q41 V42 L43 Q44 A45 R46 D47 Q48 D50 A108 T111 V112 R115 K158 Q159 D164 Y165 I170 E171 V172 E173 N174 P175 I176 L177 H178 Y179 K180 F181 H182 P183 I184 Q194 W195 Q196 T197 M199 E208 N209 I210	-7.8
8	Tellimagrandin II	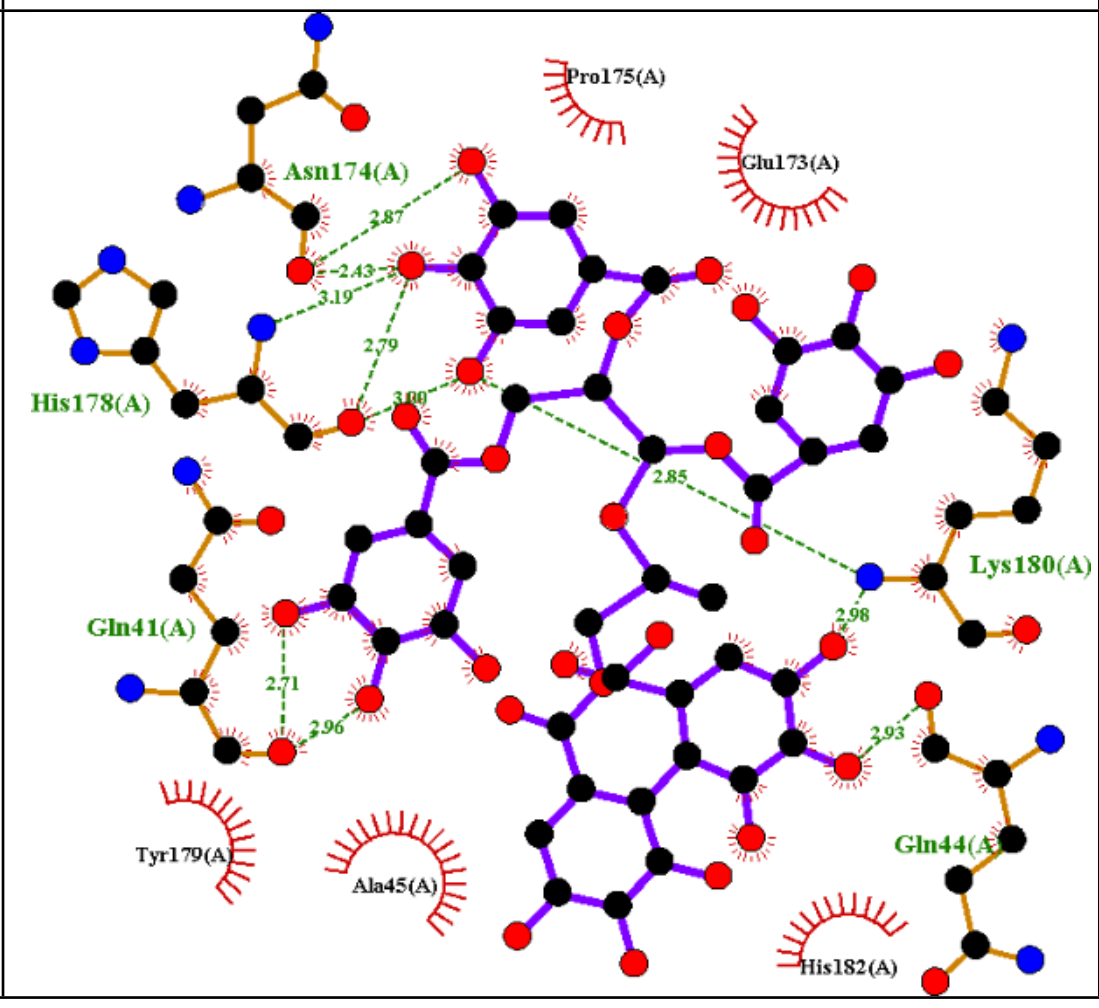	R38 L40 Q41 V42 L43 Q44 A45 R46 D47 Q48 V112 R115 K158 Q159 D164 Y165 I170 E171 V172 E173 N174 P175 I176 L177 H178 Y179 K180 F181 H182 P183 Q196 T197	-8.6

<table>
<tr><td>9</td><td>Galloylglucose</td><td>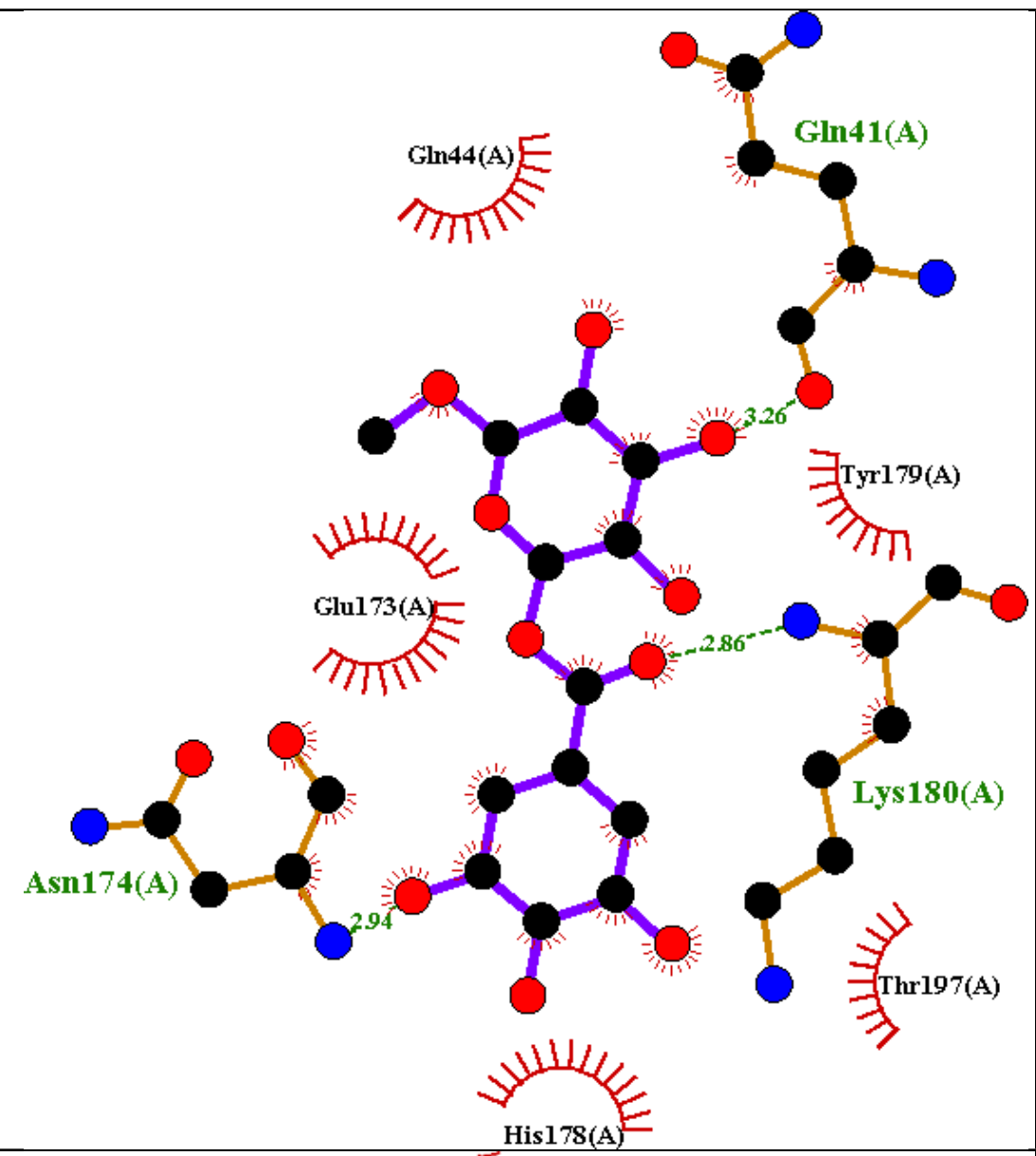
</td><td>R38 L40 Q41 V42 Q44 A45 R46 D157 K158 V172 E173 N174 P175 I176 L177 H178 Y179 K180 F181 H182 T197</td><td>-6.8</td></tr>
<tr><td>10</td><td>Vescavaloninic acid</td><td>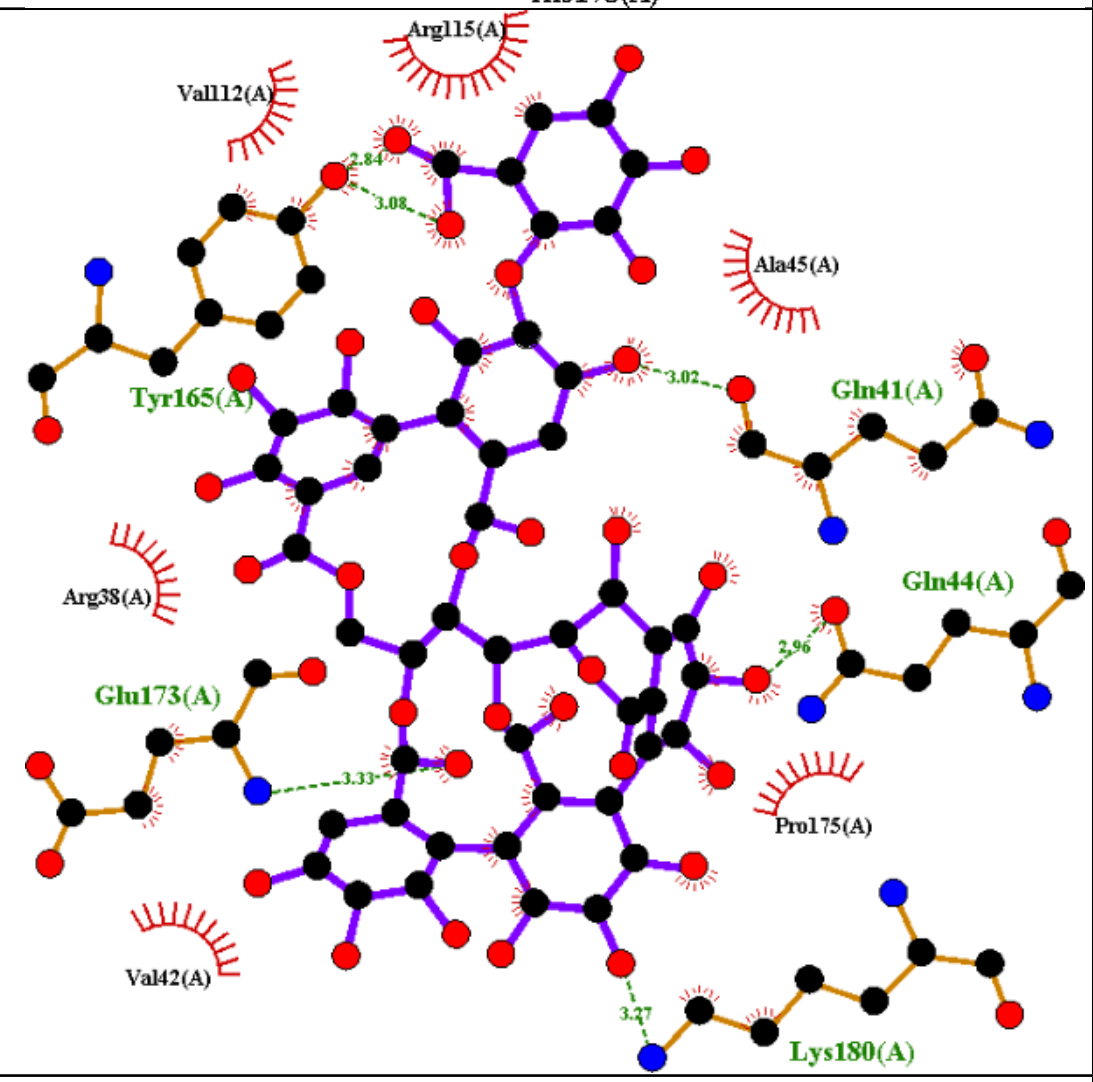
</td><td>R38 L40 Q41 V42 Q44 A45 R46 T111 V112 R115 D157 K158 Q159 D164 Y165 I170 E171 V172 E173 N174 P175 L177 H178 Y179 K180 F181 H182 P183 I184 W195 Q196 T197 M199</td><td>-8.0</td></tr>
<tr><td>11</td><td>Pentagalloylglucose</td><td>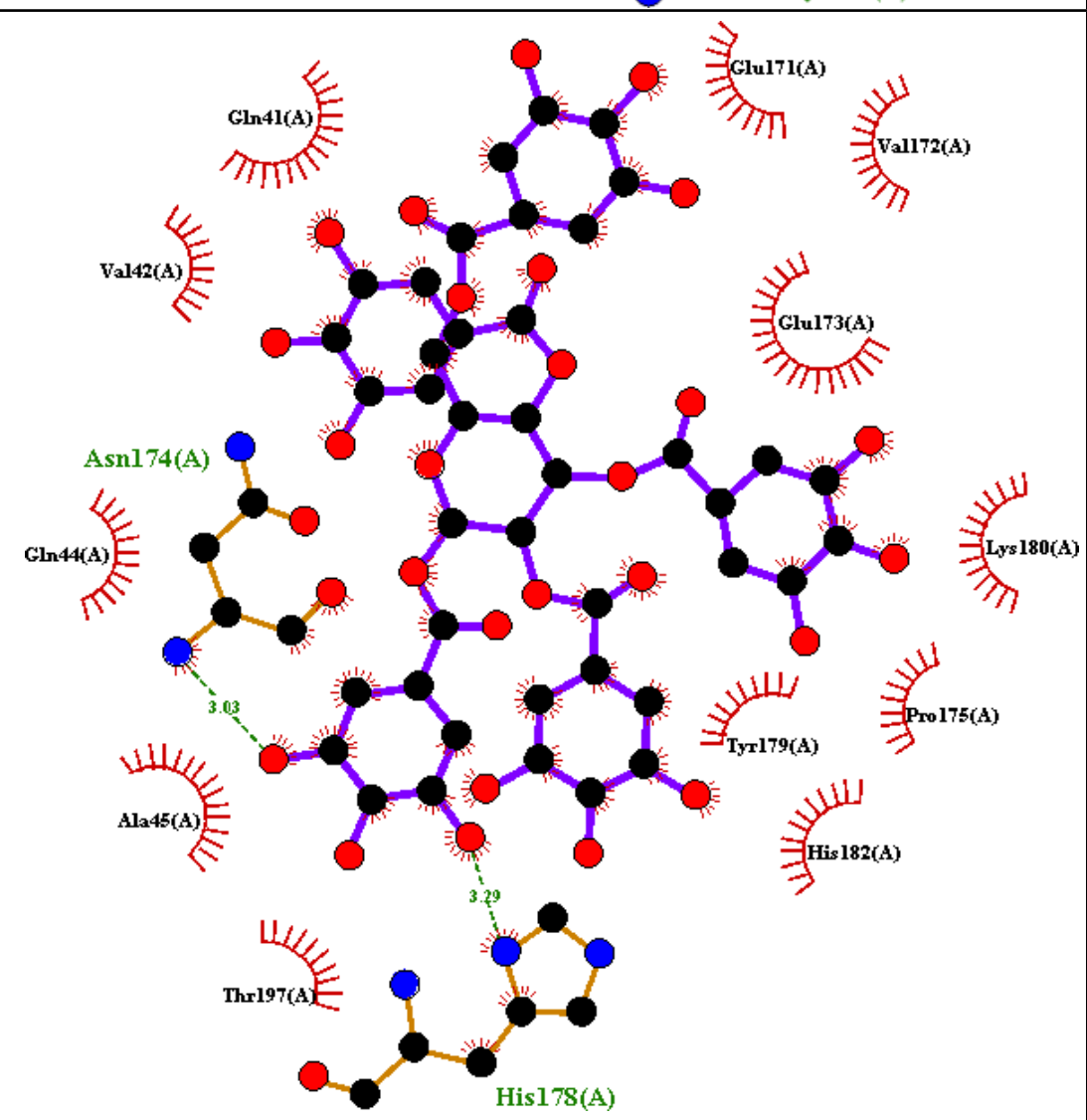
</td><td>R38 A39 L40 Q41 V42 L43 Q44 A45 R46 V112 R115 I155 R156 D157 K158 Q159 D164 Y165 I170 E171 V172 E173 N174 P175 I176 L177 H178 Y179 K180 F181 H182 Q194 W195 Q196 T197 T198 M199 K206 Q207 E208 I210 E211</td><td>-7.5</td></tr>
</table>

12	Tyrosine (control)	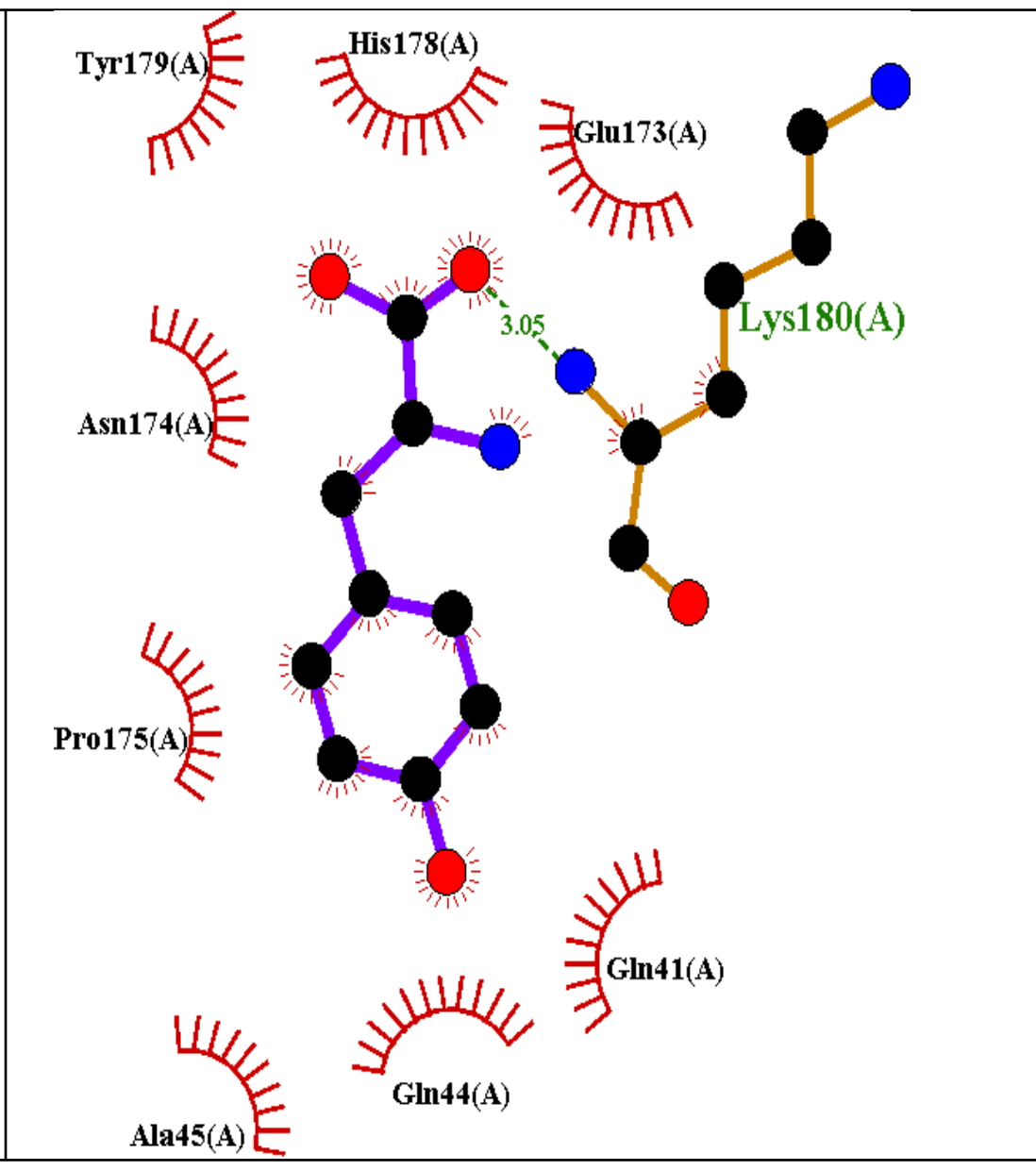	L40 Q41 V42 Q44 A45 K158 E173 N174 P175 I176 L177 H178 Y179 K180 H182 T197 E208	-5.4
α-Glucosidase (5NN8)				
No.	Compound	2D interactions	Contact residues	Affinity [kcal/mo]
1	Ellagic acid	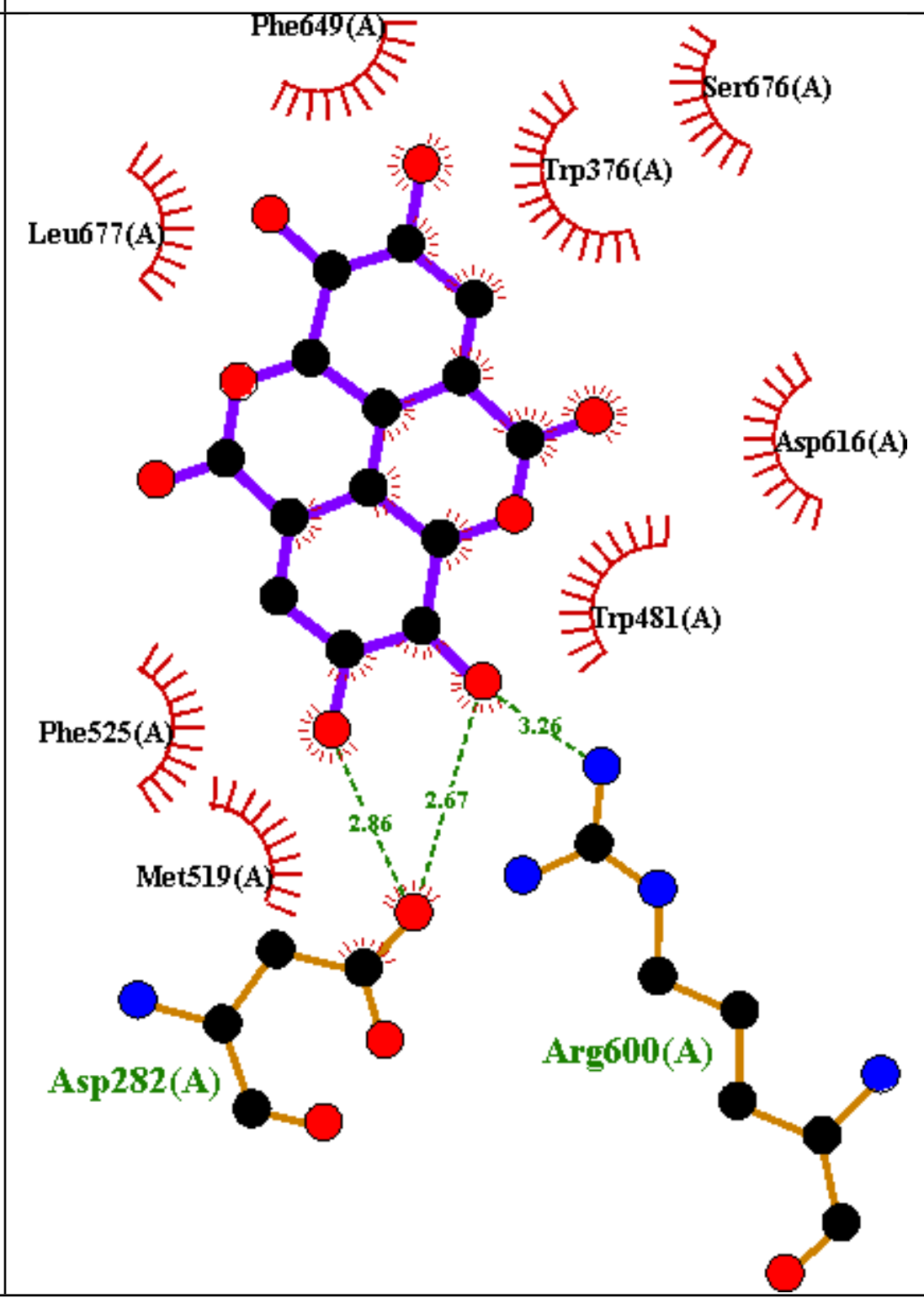	D282 L283 A284 Y292 W376 D404 I441 W481 W516 D518 M519 S523 N524 F525 R600 W613 D616 F649 L650 H674 N675 S676 L677 L678	-6.2

2	Gallic acid	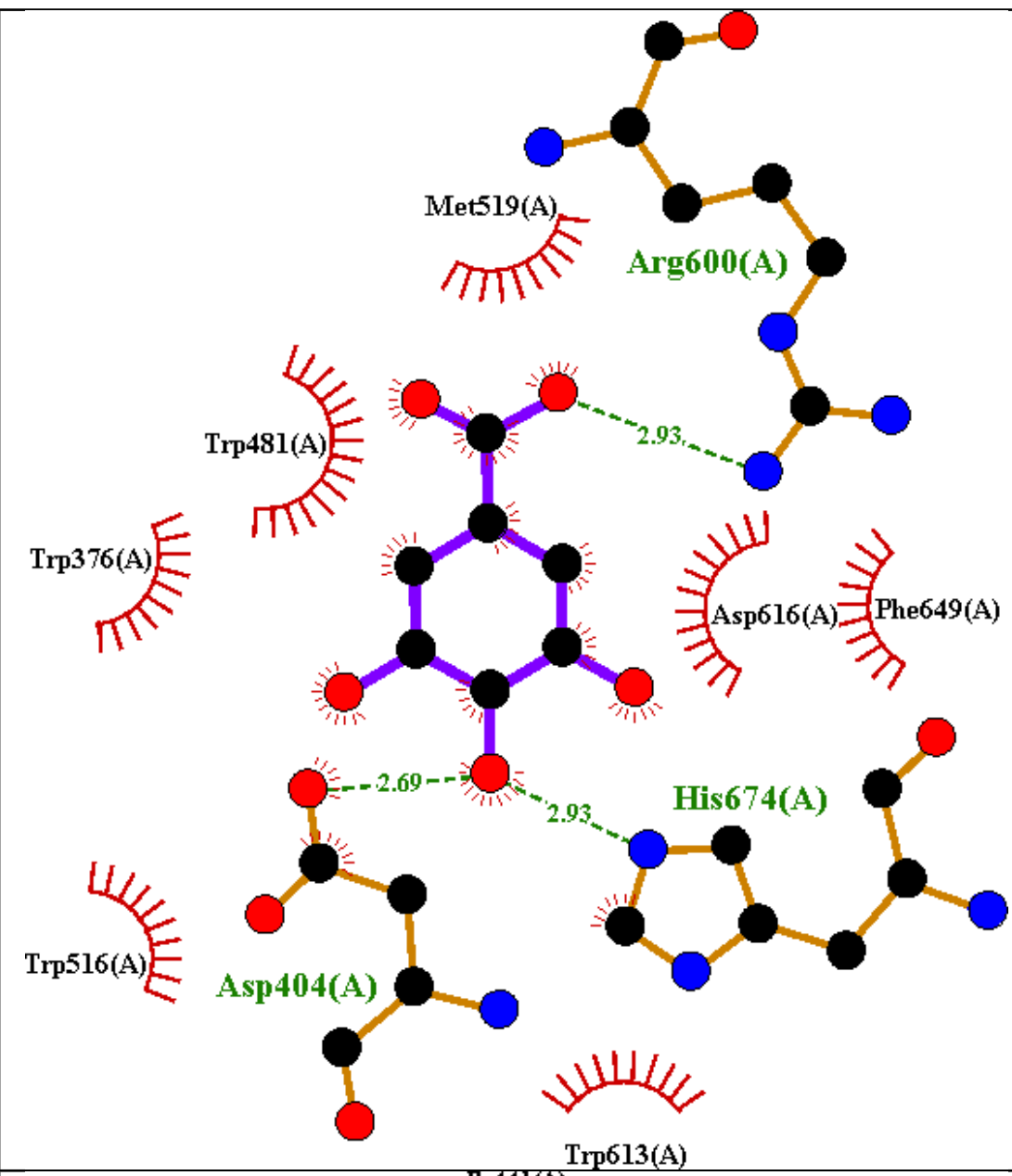	D282 W376 D404 L405 I441 D443 W481 W516 D518 M519 S523 F525 R600 W613 G615 D616 D645 F649 L650 R672 H674 S676 L677	-5.5
3	Cretanin	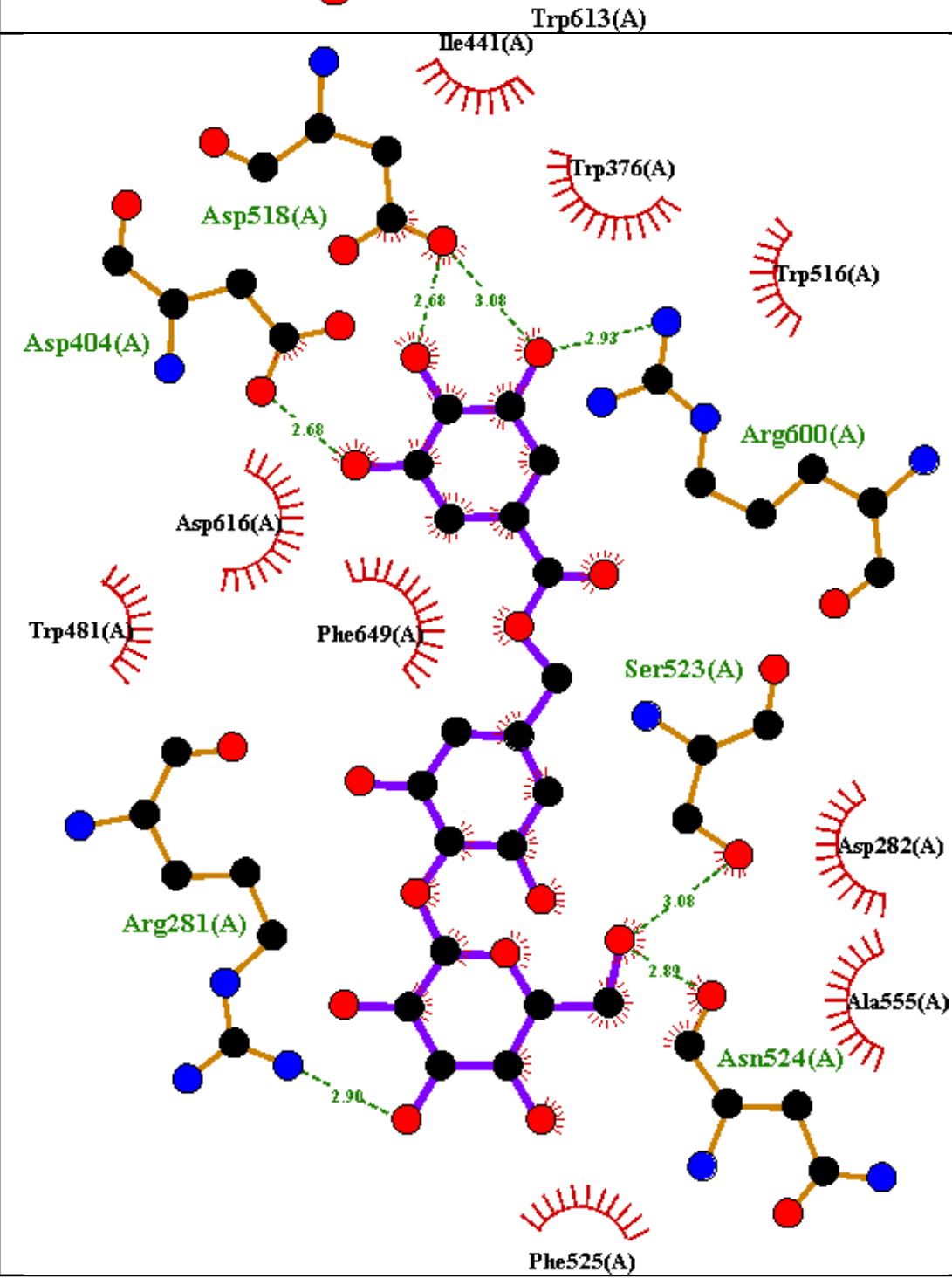	R281 D282 L283 A284 Y292 W376 D404 L405 R411 I441 V480 W481 P482 G483 W516 D518 M519 S523 N524 F525 I526 A555 T556 R600 W613 G615 D616 W618 D645 F649 L650 G651 H674 S676 L677 L678 S679	-7.4

4	Oenothein B	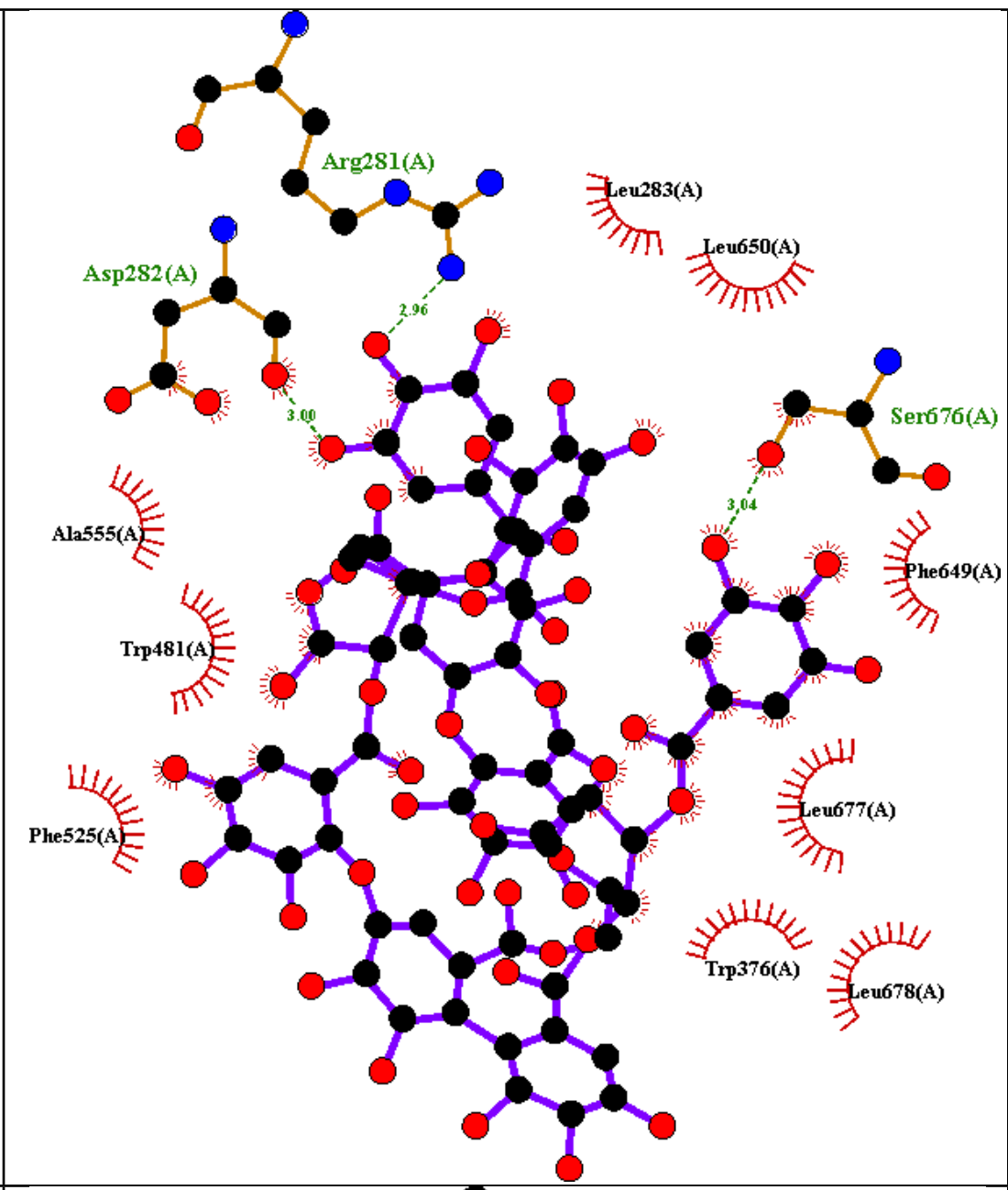	R281 D282 L283 A284 W376 R411 W481 M519 S523 N524 F525 A555 D616 W618 F649 L650 G651 S676 L677 L678 S679	-11.3
5	Vascalagin	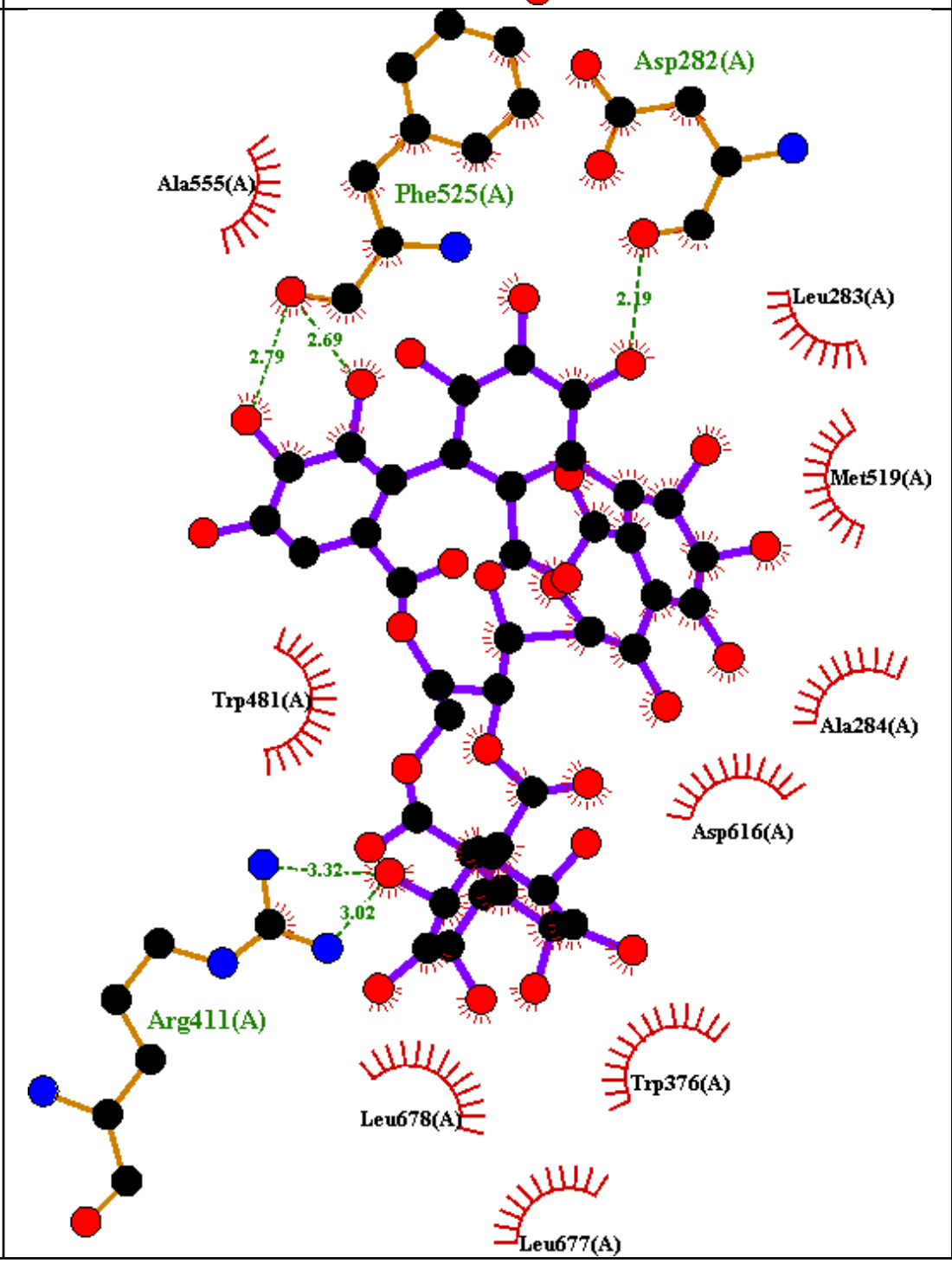	R281 D282 L283 A284 Y292 W376 R411 W481 M519 S523 N524 F525 I526 A555 R600 D616 W618 F649 L650 G651 S676 L677 L678 S679	-4.9

5	Vescalagin	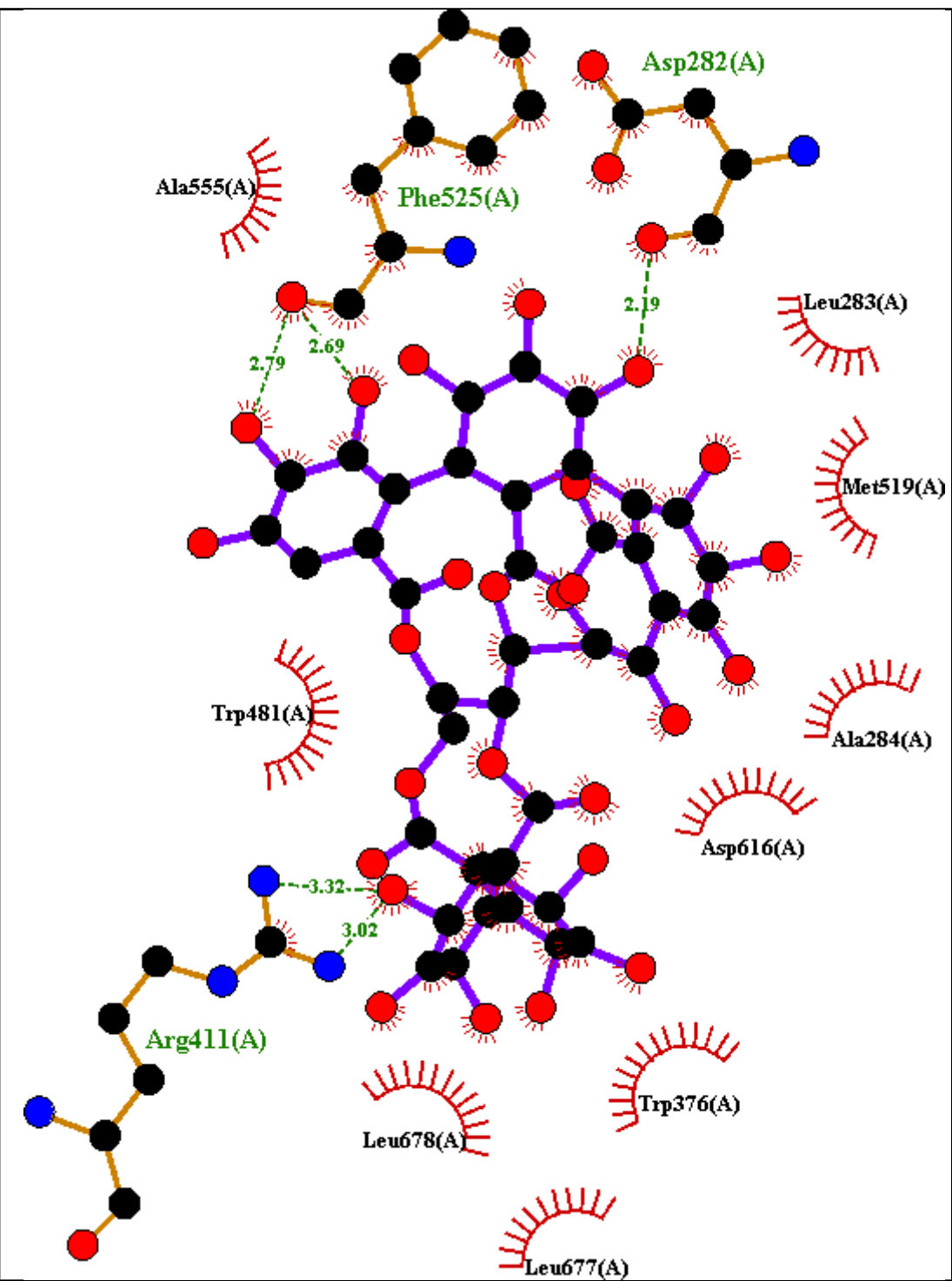	R281 D282 L283 A284 Y292 W376 R411 W481 M519 S523 N524 F525 I526 A555 R600 D616 W618 F649 L650 G651 S676 L677 L678 S679	-4.9
6	Castalagin	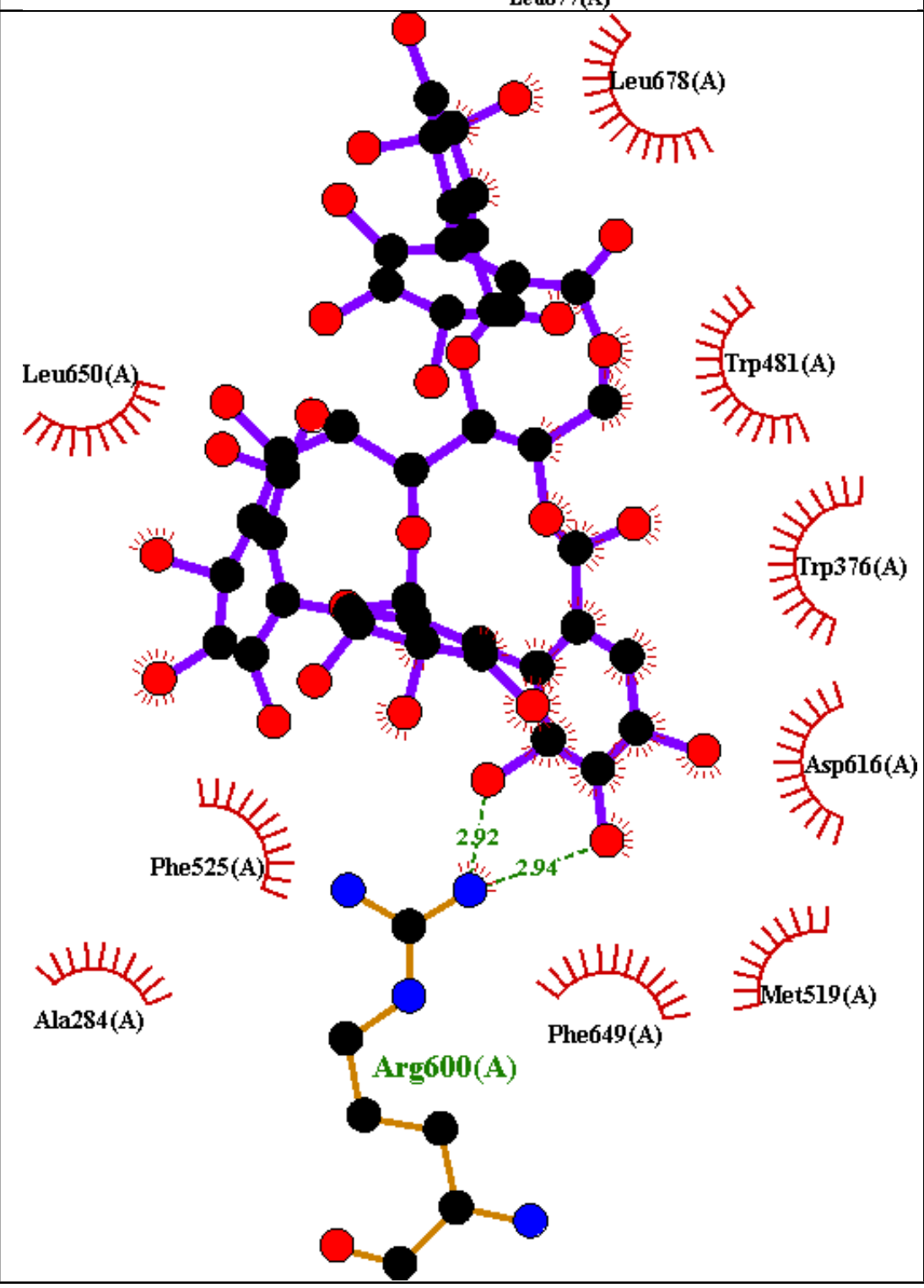	R281 D282 L283 A284 Y292 W376 D404 L405 R411 W481 W516 D518 M519 S523 N524 F525 I526 A555 R600 W613 D616 W618 F649 L650 H674 S676 L677 L678	-1.8

7	Rugosin E	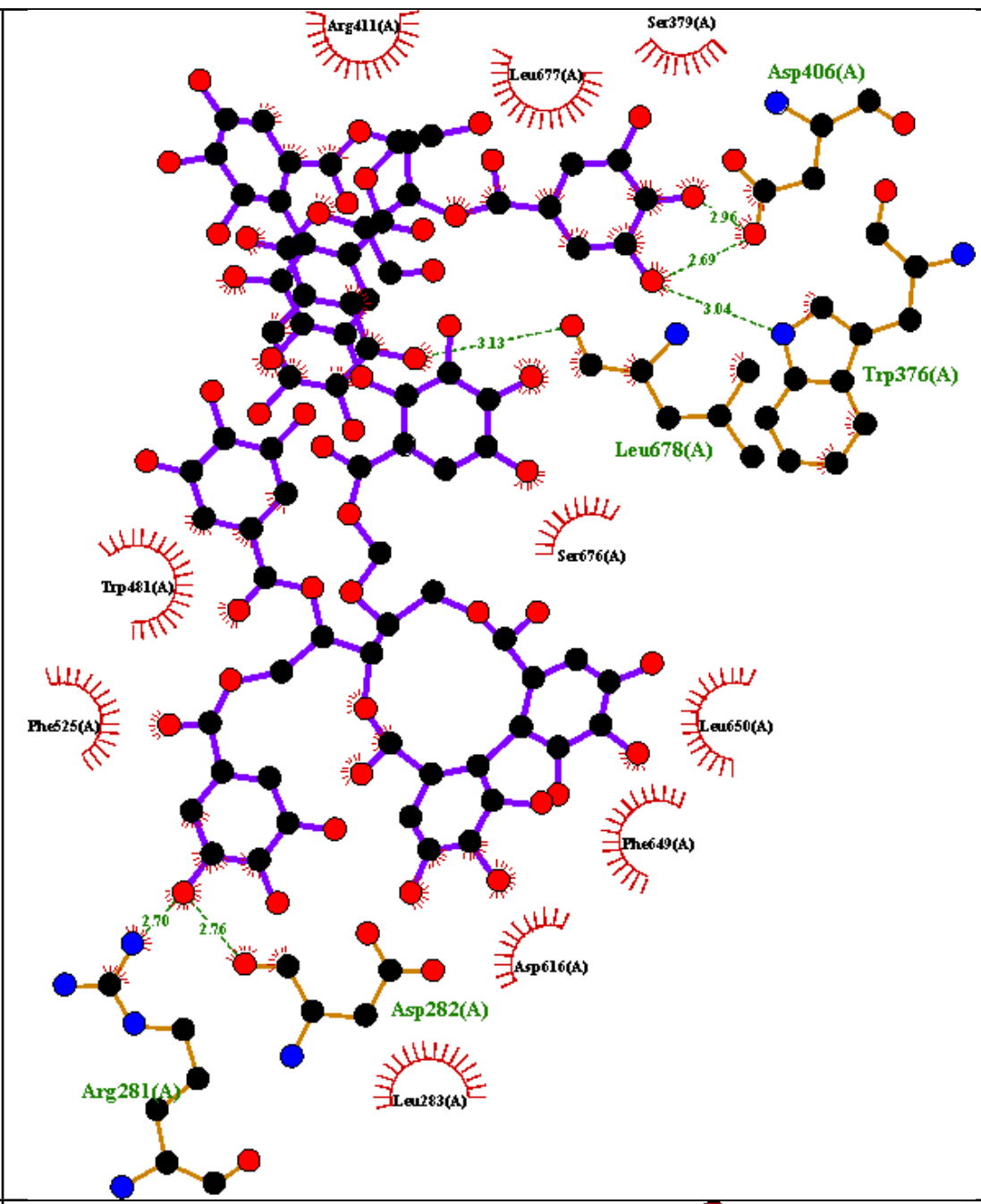	R281 D282 L283 A284 P285 T286 R375 W376 G377 Y378 S379 D404 L405 D406 S410 R411 K479 V480 W481 P482 G483 D518 M519 S523 N524 F525 I526 V548 A554 A555 R600 D616 W618 F649 L650 G651 N652 S676 L677 L678 S679	-7.6
8	Tellimagrandin II	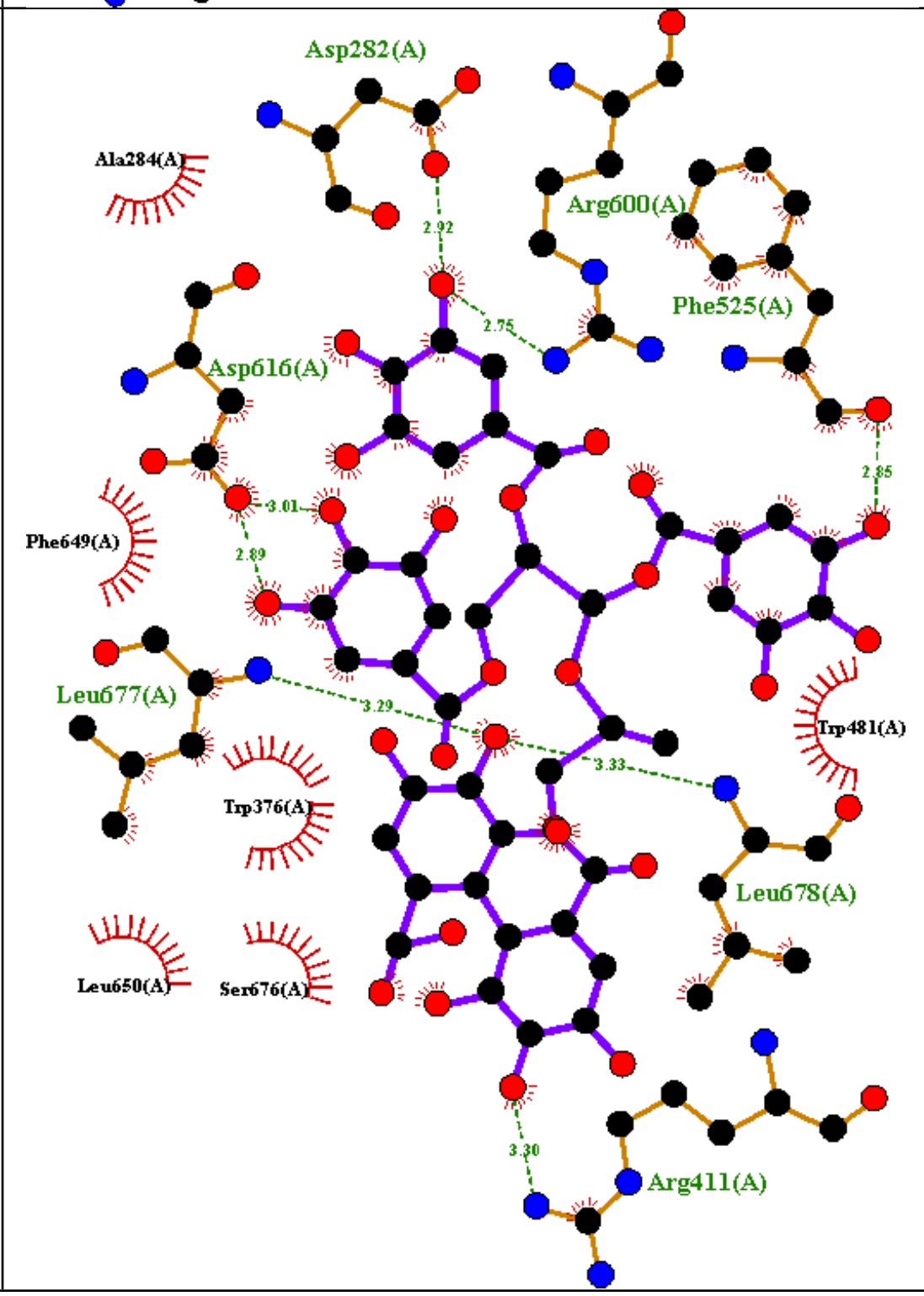	R281 D282 L283 A284 Y292 W376 D404 L405 R411 I441 D443 V480 W481 W516 D518 M519 S523 N524 F525 I526 A554 A555 R600 W613 D616 W618 D645 F649 L650 G651 H674 S676 L677 L678 S679	-7.3

9	Galloylglucose	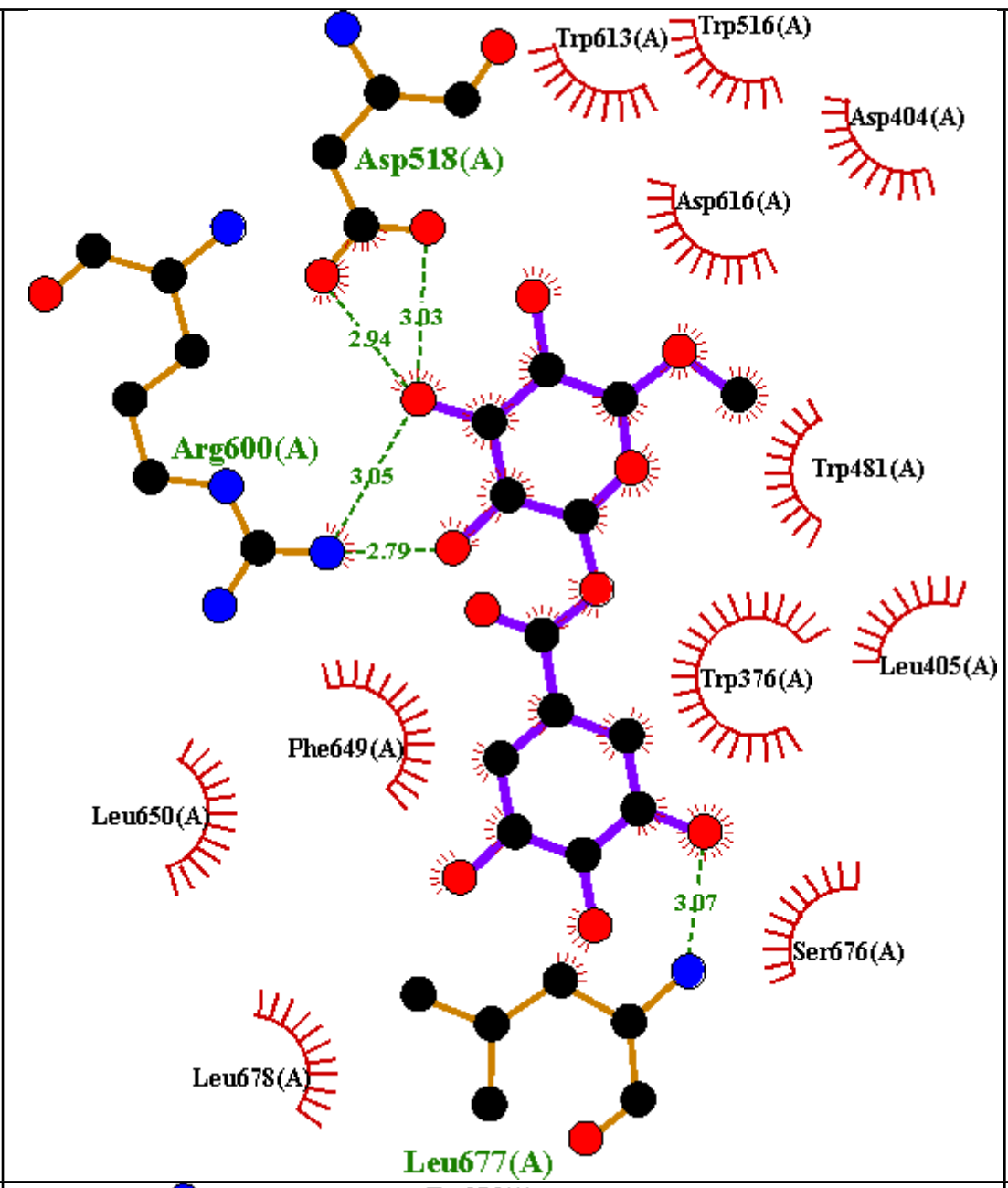	D282 L283 A284 W376 D404 L405 I441 W481 W516 D518 M519 S523 F525 R600 W613 G615 D616 D645 F649 L650 G651 R672 H674 S676 L677 L678 S679	-7.0
10	Vescavaloninic acid	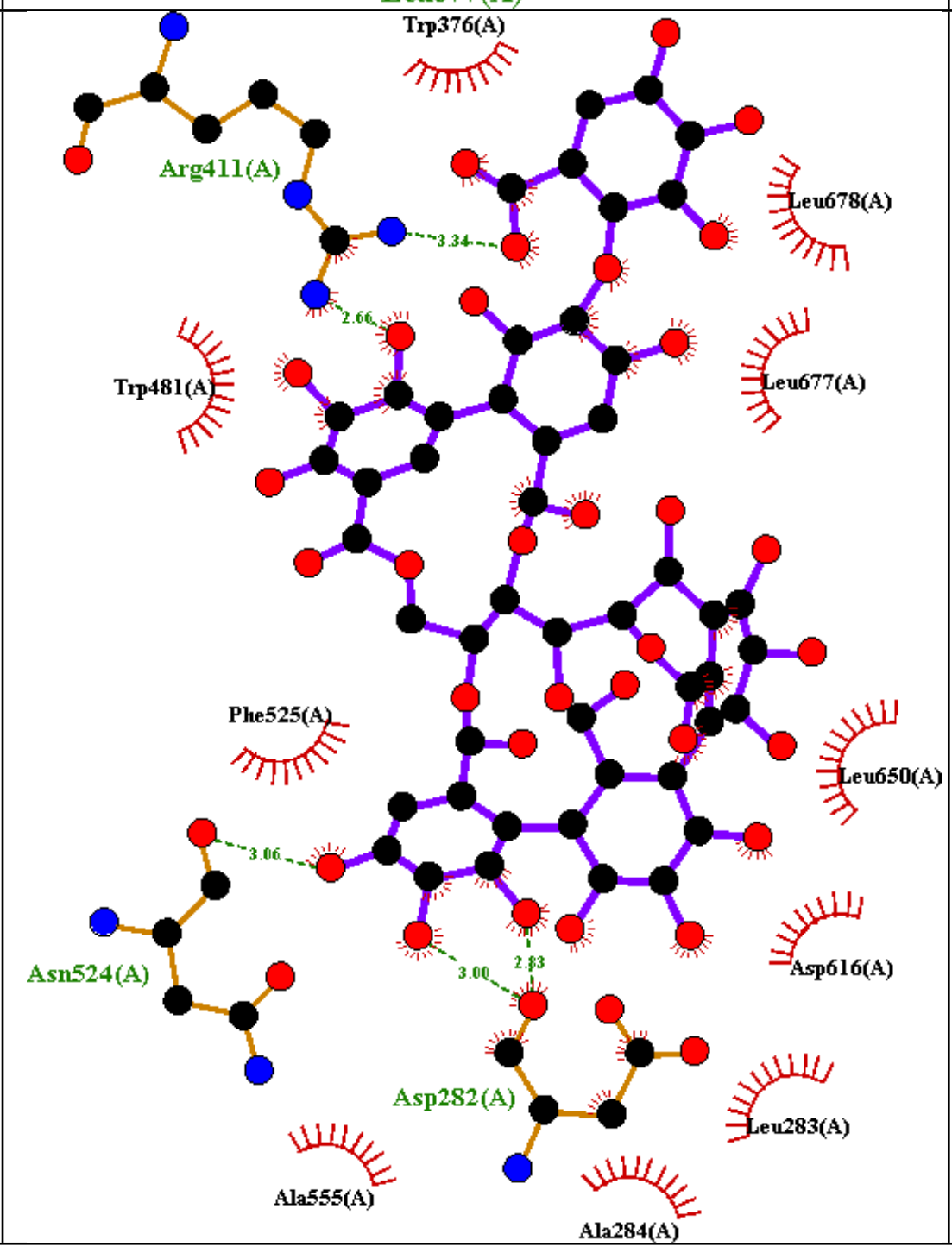	R281 D282 L283 A284 Y292 W376 R411 W481 M519 S523 N524 F525 I526 A555 R600 D616 W618 F649 L650 G651 N652 S676 L677 L678 S679 L680	-8.1

11	Pentagalloylglucose	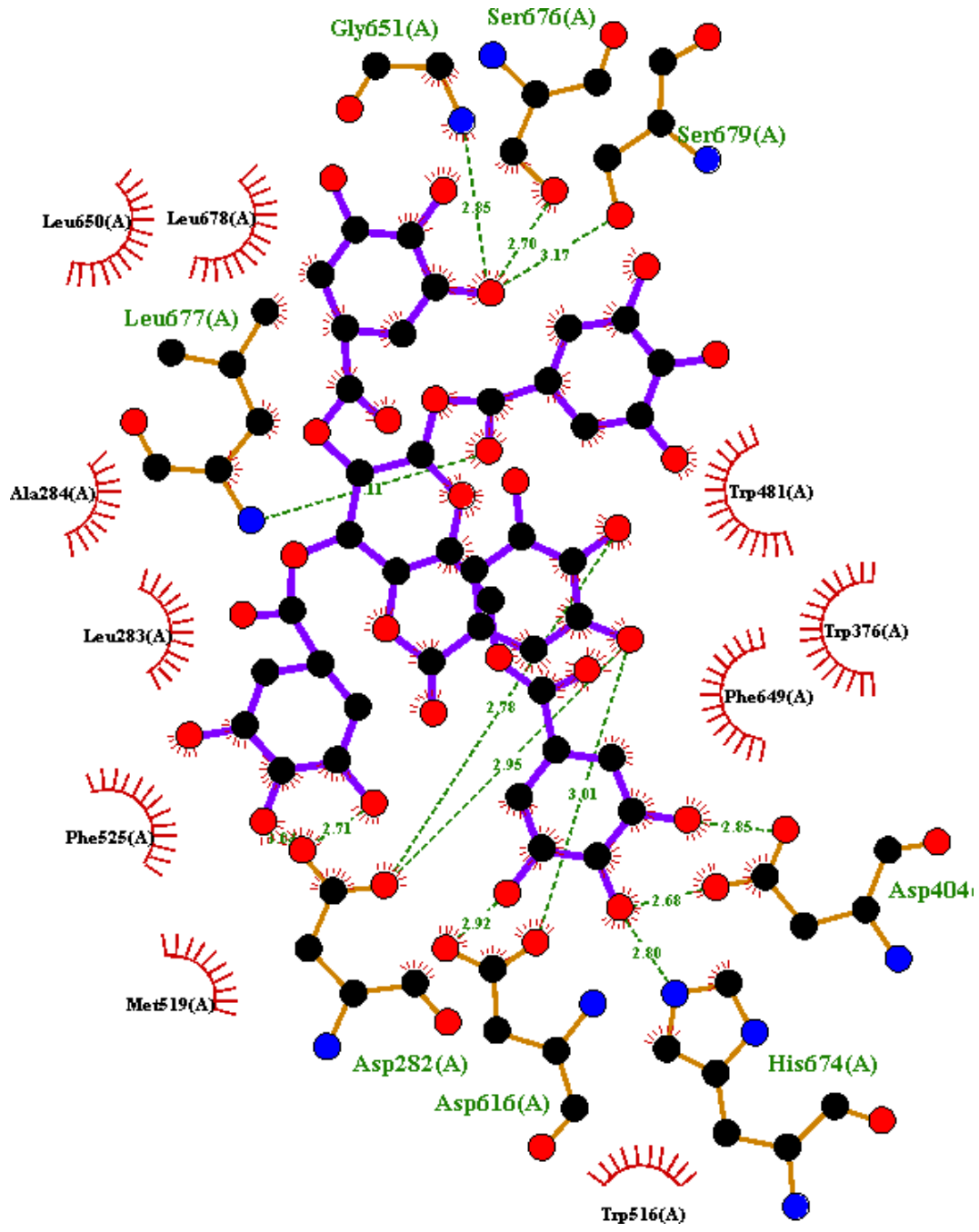	ARG281<br>ASP282<br>LEU283<br>ALA284<br>PRO285<br>TYR292<br>TRP376<br>ASP404<br>LEU405<br>ARG411<br>ILE441<br>ASP443<br>VAL480<br>TRP481<br>TRP516<br>ASP518<br>MET519<br>SER523<br>ASN524<br>PHE525<br>ILE526<br>ALA554<br>ALA555<br>ARG600<br>TRP613<br>GLY615<br>ASP616<br>TRP618<br>ASP645<br>PHE649<br>LEU650<br>GLY651<br>ASN652<br>ARG672<br>HIS674<br>SER676<br>LEU677<br>LEU678<br>SER679	-9.1

12	Acarbose (control)	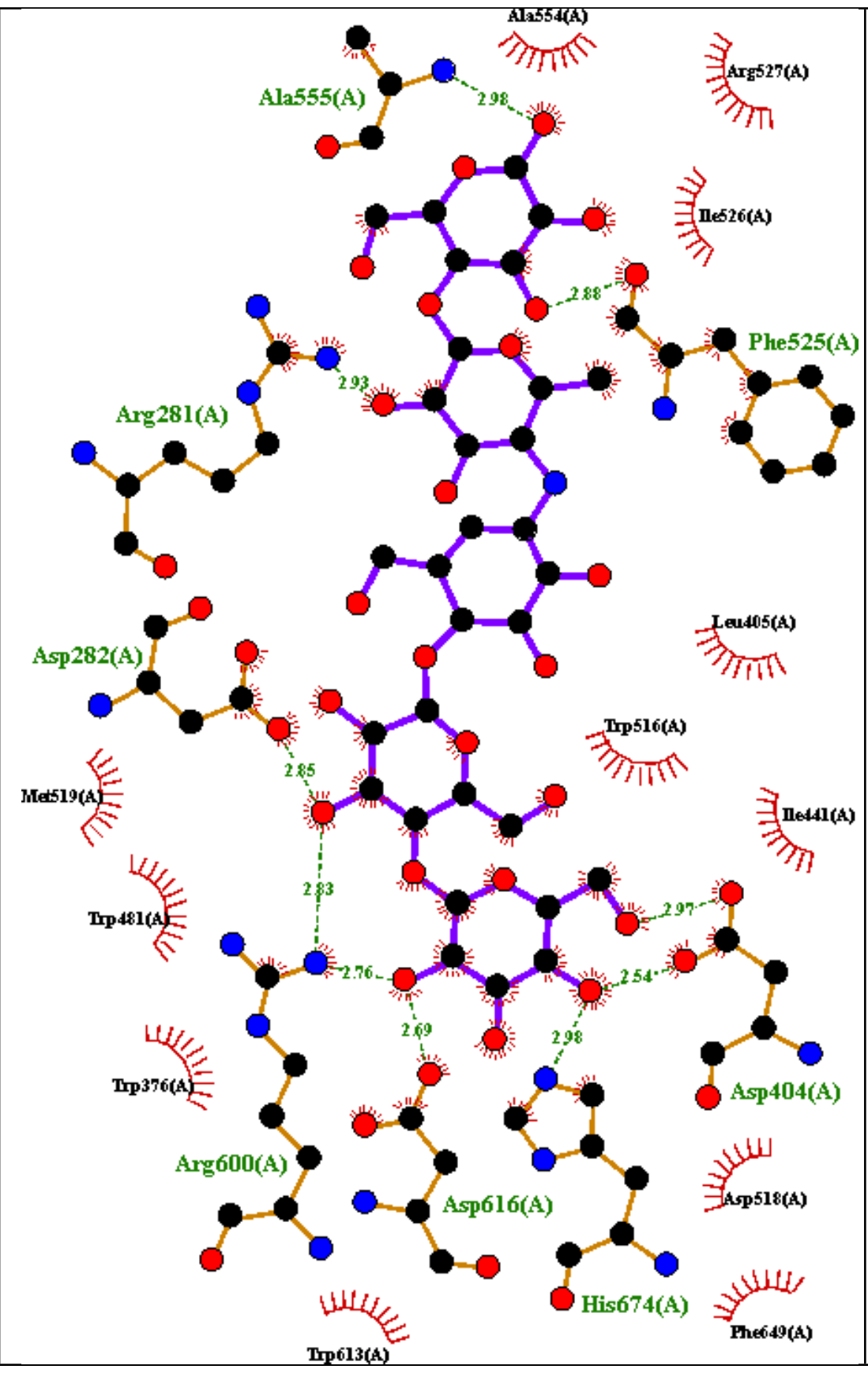	R281 D282<br>L283 A284<br>Y292 W376<br>D404 L405<br>R411 I441<br>D443 K479<br>V480 W481<br>P482 G483<br>W516 D518<br>M519 S523<br>N524 F525<br>I526 R527<br>V548 Q553<br>A554 A555<br>T556 R600<br>W613 G615<br>D616 W618<br>F649 L650<br>G651 N652<br>R672 H674<br>S676 L677<br>L678 S679	-7.4

Table S2 *In silico* pharmacokinetic properties and toxicity profiles of selected active compounds found in *Q. rubra* and *Q. robur* acorns

Compound	Absorption		Distribution		Metabolism		Excretion	Toxicity			
	Caco2 permeability (log Papp in 10 cm/s)	Intestinal absorption (human) (%)	BBB permeability (log BB)	CNS permeability (log PS)	CYP3A4 substrate (Yes/No)	CYP3A4 inhibitor (Yes/No)	Total Clearance (log ml/min/kg)	Max. tolerated dose (human) (log mg/kg/day)	Oral Toxicity Predicted LD_{50} (mg/kg)	Oral Rat Chronic Toxicity LOAEL (log mg/kg bw/day)	Minnow toxicity (log mM)
Gallic acid	-0.467	50.311	-0.930	-2.816	No	No	0.550	1.404	2,000	1.499	2.918
Ellagic acid	0.273	80.032	-1.214	-3.144	No	No	0.539	0.777	2,991	1.947	2.585
Galloylglucose	-0.598	39.335	-1.475	-4.357	No	No	0.546	0.828	2,260	1.702	4.282
Digalloylglucose	-1.650	15.702	-2.422	-4.677	No	No	0.433	0.461	2,190	4.552	7.440
Trigalloylglucose	-1.156	24.113	-2.880	-4.892	No	No	0.505	0.546	2,260	2.660	3.510
Tetragalloylglucose	-1.435	16.502	-3.582	-5.160	No	No	0.511	0.423	2,260	3.139	3.124
Pentagalloylglucose	-1.714	8.891	-4.285	-5.427	No	No	0.524	0.391	2,260	3.618	2.738
Castalagin/ Vescalagin	-1.586	24.852	-3.931	-5.149	No	No	0.114	0.308	2,170	2.222	3.636
Casuarictin	-1.635	20.027	-4.018	-5.078	No	No	0.235	0.318	2,260	3.660	3.248
Oenothein B	-3.127	88.955	-6.903	-8.438	No	No	-7.945	0.438	2,260	14.457	23.625
Punicalagin	-1.719	14.434	-4.330	-5.216	No	No	0.477	0.403	3,700	4.145	3.548
Rugosin E	-4.003	29.832	-8.275	-9.097	No	No	-8.335	0.438	2,260	15.935	23.096
Tellimagrandin II	-1.674	14.496	-4.137	-5.229	No	No	0.368	0.347	2,260	3.645	3.017
Vescavaloninic/ acid	-0.917	76.635	-6.060	-7.563	No	No	0.508	0.438	2,170	19.47	25.27
Cretanin	-0.779	32.307	-1.994	-4.568	No	No	0.489	0.715	5,000	2.203	3.792

pkCSM and ProTox-3.0 predictions were performed on 23 September 2026 using default settings.
